# Synthesis, Antibacterial and Antiribosomal Activity of the 3*C*-Aminoalkyl Modification in the Ribofuranosyl Ring of Apralogs (5-*O*-Ribofuranosyl Apramycins)

**DOI:** 10.3390/antibiotics12010025

**Published:** 2022-12-24

**Authors:** Dmitrijs Lubriks, Klara Haldimann, Sven N. Hobbie, Andrea Vasella, Edgars Suna, David Crich

**Affiliations:** 1 Latvian Institute of Organic Synthesis, Aizkraukles 21, LV-1006 Riga, Latvia; 2 Institute of Medical Microbiology, University of Zurich, Gloriastrasse 30, 8006 Zürich, Switzerland; 3 Organic Chemistry Laboratory, ETH Zürich, Vladimir-Prelog-Weg 1-5/10, 8093 Zürich, Switzerland; 4 Department of Pharmaceutical and Biomedical Sciences, University of Georgia, 250 West Green Street, Athens, GA 30602, USA; 5 Complex Carbohydrate Research Center, University of Georgia, 315 Riverbend Road, Athens, GA 30602, USA; 6 Department of Chemistry, University of Georgia, 302 East Campus Road, Athens, GA 30602, USA

**Keywords:** aminoglycoside modifying enzymes, ribosomal methyltransferases, antibacterials, antiribosomal activity, aminoglycoside antibiotics

## Abstract

The synthesis and antiribosomal and antibacterial activity of both anomers of a novel apralog, 5-*O*-(5-amino-3-*C*-dimethylaminopropyl-D-ribofuranosyl)apramycin, are reported. Both anomers show excellent activity for the inhibition of bacterial ribosomes and that of MRSA and various wild-type Gram negative pathogens. The new compounds retain activity in the presence of the aminoglycoside phosphoryltransferase aminoglycoside modifying enzymes that act on the primary hydroxy group of typical 4,5-(2-deoxystreptamine)-type aminoglycoside and related apramycin derivatives. Unexpectedly, the two anomers have comparable activity both for the inhibition of bacterial ribosomes and of the various bacterial strains tested.

## 1. Introduction

**Figure 1:**
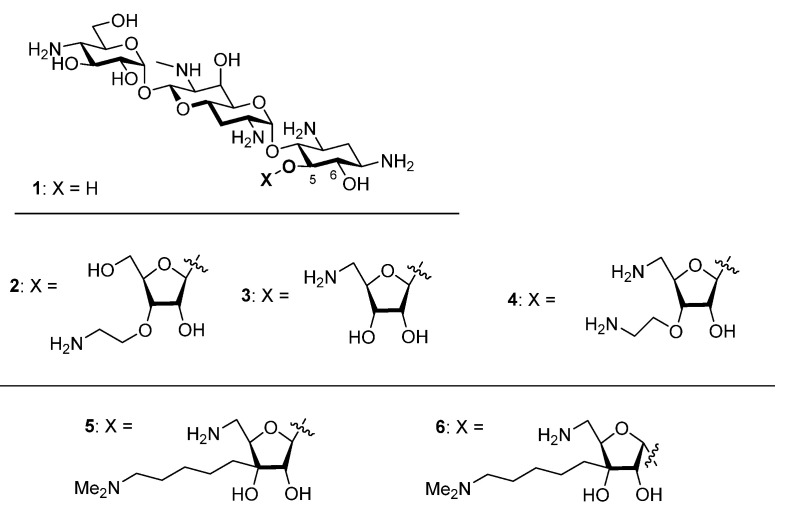
Apramycin **1**, Apralogs **2–4**, and the Target Compounds **5** and **6**.

In our quest to improve the antibacterial activity of apramycin **1**, an atypical 2-deoxystreptamine-type aminoglycoside antibiotic with reduced toxicity, minimal susceptibility to aminoglycoside modifying enzymes [1,2,3,4] (AMEs) and ribosomal methyltransferases (RMTs), and strong activity against a broad spectrum of ESKAPE pathogens [5,6,7,8,9,10,11,12,13,14,15,16,17,18,19], we have developed the 5-*O*-furanosyl apramycins, or apralogs [20,21,22,23,24]. The present optimal apralogs carry aminoalkyl substituents at the 3-position of the furanosyl ring, eg, **2**, and/or aminodeoxy substitution at the 5-position of the furanose ring as in **3** and **4**, and have increased levels of activity against ESKAPE pathogens while retaining the outstanding toxicity profile and minimal susceptibility to resistance mechanisms that characterize apramycin itself. In our continuing quest to further improve the apralogs we designed and report here on the synthesis and evaluation of the new apralogs **5** and **6**. Like the previous apralogs **2** and **4**, these novel derivatives carry activity-enhancing aminoalkyl substituents at the ribose 3-position, but now appended via a carbon-carbon bond as opposed to the previous ether linkages. This modification allows the retention of a hydroxy group at the ribose 3-position with the potential to engage in adventitious hydrogen bonding interactions in the hydrated binding site and the consequent potential to further increase activity and selectivity (Figure 1). Ultimately, we find that **5** and **6** have essentially identical activity and ribosomal selectivity, indicating that the modifications introduced override the importance of anomeric configuration in the ribofuranosyl bond that characterized the early apralogs.

## 2. Results

### 2.1. Synthesis

**Scheme 1:**
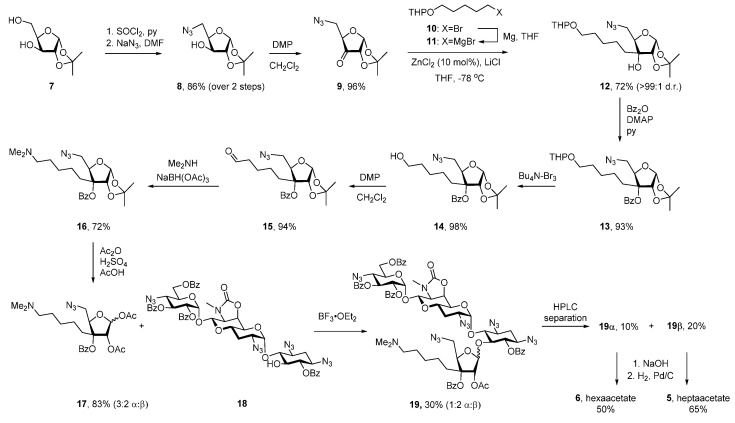
Synthesis of Apralogs **5** and **6**.

Apralogs **5** and **6** were synthesized by glycosylation of selectively protected apramycin derivative **18** with glycosyl donor **17** as the key step (Scheme 1). Alcohol **18** was accessed from apramycin **1** in four steps as described previously [25,26], whereas 3C-aminoalkyl ribofuranose **17** was synthesized from protected xylofuranose **7** (Scheme 1). Thus, treatment of **7** with thionyl chloride in pyridine furnished cyclic sulfite [27] that was further reacted with NaN_3_ to afford **8** in 86% yield. Subsequent oxidation with Dess-Martin periodinane (DMP) delivered ketone **9** that underwent highly stereoselective addition of Grignard reagent **11**, prepared from THP-protected bromopentanol **10** [28] and metallic magnesium. Subsequent benzoyl protection of the resulting tertiary alcohol was followed by THP cleavage with Bu_4_N-Br_3_ and oxidation of the so-formed primary alcohol to aldehyde **15** in 86% yield over three steps from **12**. The desired *N,N*-dimethylamino moiety was installed by reductive amination of **15** in 72% yield, after which a swap of the acetonide protection for the corresponding 2,3-diacetate delivered glycosyl donor **17** in 83% yield as a 3:2 mixture of α:β-anomers.

Glycosidic bond formation between glycosyl donor **17** and alcohol **18** was a non-trivial task. Initial attempts using excess (6 equiv) of BF_3_ OEt_2_, TMS-OTf and TES-OTf as acidic promoters in the presence of 3Å MS (type A zeolite, 400 mg per mmol of **17**) as water scavenger only led to 5% conversion of alcohol **18**, perhaps because of the alkaline nature of zeolite sieves [29]. While pretreatment of the molecular sieves with acid resulted in a slight improvement of the BF_3_ OEt_2_-promoted glycosylation (10%), a preparative useful 30% yield of the desired **19** was eventually obtained in the absence of molecular sieves. Under these conditions glycoside **19** was formed as 1:2 mixture of α:β-anomers that were obtained as individual isomers after straightforward separation from unreacted **18** and hydrolyzed glycosyl donor **17** by preparative HPLC. Each of epimeric glycosides **19β** and **19α** was deprotected by a sequence of saponification, followed by hydrogenolysis of azides (Scheme 1), with final purification achieved by preparative HPLC, followed by treatment with acetic acid and trituration with MeCN to give apralogs **5** and **6** in the form of their peracetate salts.

### 2.2. Activity and Selectivity at the Drug Target

**Figure 2:**
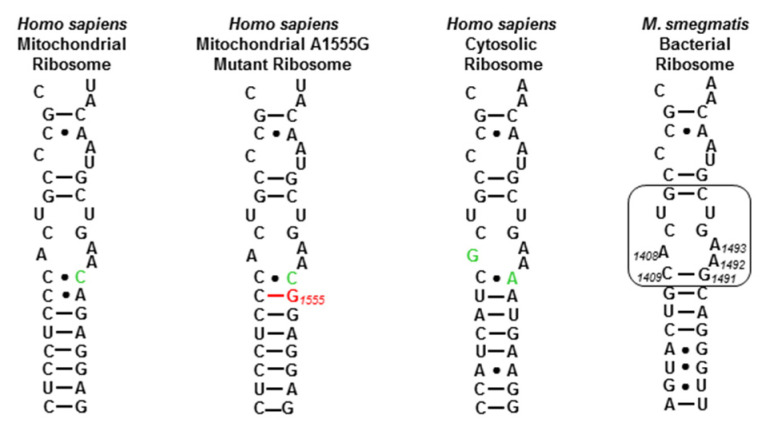
Decoding A sites of prokaryotic and eukaryotic ribosomes. The bacterial AGA binding pocket is boxed. The bacterial numbering scheme is illustrated for the AGA binding pocket. Changes from the bacterial ribosome binding pocket are colored green. The A1555G mutant conferring hypersusceptibility to AGA ototoxicity is colored red.

We first checked the activity of the new apralogs for activity at the target level, the ribosomal decoding A site [30,31,32,33,34,35,36], through their ability to disrupt bacterial protein synthesis in cell-free translation assays [37], with apramycin **1** and the apralogs **2**, **3** and **4** as comparators (Table 1). We also screened for inhibition of protein synthesis by a set of humanized bacterial ribosomes in which the complete bacterial decoding A site has been replaced by that of the human mitochondrial (Mit13) or A1555G mutant mitochondrial ribosome (A1555G) (Figure 2) [38], as AGA binding to the cognate decoding A sites of the human mitochondrial and especially the A1555G mutant mitochondrial ribosomes in the cochlea is considered to be one of the main causes of AGA-induced ototoxicity [30,39,40,41,42,43,44,45]. Finally, we screened for inhibition of protein synthesis by similarly engineered bacterial ribosomes carrying the human cytosolic decoding A site (Cyt14) to assess the possibility of broader systemic toxicity (Figure 2).

Compounds **5** and **6** show very similar levels of activity for the inhibition of the wild-type bacterial ribosome and for that of the hybrid ribosomes carrying the eukaryotic decoding A sites, indicating that the anomeric configuration of the ribofuranosyl ring is of no consequence in this pair of isomers. The activity of **5** and **6** against the wild-type bacterial ribosome is comparable to that of **2**, 2-fold better than of apramycin itself and the apralog **3** and 2–3-fold-less than that of apralog **4**. In terms of selectivity for the bacterial ribosome over the three eukaryotic hybrid ribosomes, the two novel compounds retain the overall favorable profile of apramycin and the apralogs in general (Table 1).

### 2.3. Antibacterial Activity against Wild-Type Bacterial Strains

All newly prepared compounds and the comparators were tested for activity against a series of ESKAPE pathogens made up of a Gram-positive methicillin-resistant *Staphylococcus aureus* (MRSA) strain, and a panel of wild-type Gram negative pathogens (*Escherichia coli*, *Klebsiella pneumoniae*, *Enterobacter cloacae*, *Acinetobacter baumannii*, *Pseudomonas aeruginosa*) (Table 2).

Consonant with their inhibition of the wild-type bacterial ribosomes, compounds **5** and **6** have very similar antibacterial activity against MRSA and the wild-type Gram negative pathogens screened (Table 2). Again in agreement with the antiribosomal activities, the two compounds display comparable activity to apramycin itself and to the apralogs **2** and **3**, and 2-fold less activity than **4** against all pathogens tested, with the exceptions of *Acinetobacter baumannii* and *Pseudomonas aeruginosa*, where they showed 4–8 fold less activity.

### 2.4. Antibacterial Activity against Resistant Bacterial Strains

To gauge the ability of the new apralogs to overcome resistance due to the presence of AMEs, they were screened against a panel of engineered *E. coli* each member of which carries a specific resistance determinant (Table 3). Four APH isoforms were included in this survey, together with one bearing the AAC(3)-IV AME known to be problematic in the apramycin series [20,21], and two carrying G1405-acting RMTs (ArmA and RmtB), which strongly mitigate the activity of all DOS-type AGAs currently used in the clinic (Table 3).

As indicated in Table 3, **5** and **6** retained excellent activity against *E. coli* strains bearing four different APH(3′) isoforms and in particular against the APH(3′,5′′)-Ia isoform [46], which has the ability to phosphorylate at the ribose 5-position and so abrogate the activity of the 4,5-DOS AGAs in general and of apralogs such as **2** that retain the hydroxy group in the ribose side chain. Notably, like other apralogs, **5** and **6** afford a significant measure of protection against the action of the AAC(3)-IV isozyme, the only AME with the ability to modify and reduce the activity of apramycin itself [47]. Finally, the novel modification in **5** and **6** does not lead to resistance arising from the presence of ribosomal methyltransferases acting on G1405 [48].

### 2.5. Discussion

**Figure 3:**
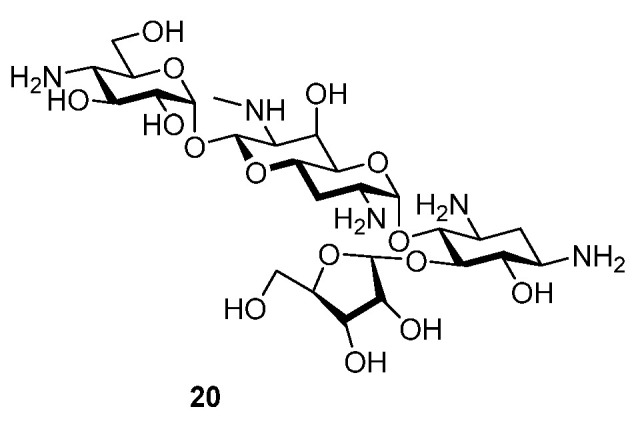
Structure of Apralog **20**.

Compounds **5** and **6** retain excellent levels of activity for inhibition of the bacterial ribosome and correspondingly strong levels of antibacterial activity against MRSA and wild-type Gram negative pathogens. Compounds **5** and **6** show comparable selectivity for inhibition of the bacterial ribosome over the eukaryotic ribosomes to other apralogs and a similar profile to other 5′′-amino-5′′-deoxy apralogs when challenged with *E. coli* carrying the APH(3′)-Ia and AAC(3)-IV AMEs. As such the novel 3-*C*-(aminoalkyl)-3-hydroxy modification in the ribose ring of the apralogs is a viable modification, but based on the present data does not offer any particular advantages over the existing series of compounds and in particular the advanced apralog **4**. It is, however, noteworthy, that the antiribosomal and antibacterial activities of the two compounds are essentially identical, indicating that the anomeric configuration in the ribofuranosyl ring is of no consequence in this series. This observation differs significantly from that previously reported for **2** and its α-ribofuranosyl epimer **20** (Figure 3), where the β-isomer was some 400 times more active for inhibition of the bacterial ribosome, and between 2- and 8-fold more active in MIC assays against wild-type Gram negative organisms [20]. As the β-ribofuranosyl configuration is usually necessary to position the primary side chain hydroxyl group of the ribose moiety for a critical hydrogen bonding interaction with both N2′ in ring I and with G1491 in the drug binding pocket, this result suggests that the N2′-OH/NH_2_5′’-G1491 hydrogen bond is not critical in the present molecules. This is presumably because of the presence of six basic amines, which we have previously shown surmounts the importance of this hydrogen bond [22].

## 3. Conclusions

The synthesis of the α- and β-anomers of a novel 5-*O*-(3*C*-aminoalkyl-5-aminoribofuranosyl)apramycin is described. The new modification affords strong activity for the inhibition of protein synthesis by the bacterial ribosome, and for the inhibition of MRSA and typical Gram-negative pathogens. Consistent with other apralogs carrying the 5-amino-5-deoxy modification in the ribofuranosyl ring, the new compounds are not susceptible to deactivation by the APH(3′,5′′)-Ia type AME. Unexpectedly, both anomers of the new compound show essentially identical activity.

## 4. Materials and Methods

### 4.1. General Experimental

All reagents and solvents were purchased from commercial suppliers and were used without further purification unless otherwise specified. All experiments were carried out under a dry argon atmosphere unless otherwise specified. Unless noted otherwise, progress of reactions was monitored by thin-layer chromatography on pre-coated aluminum-backed silica gel plates (Merck Kieselgel 60F_254_, Merck, Darmstadt, Germany) and were visualized by UV light (254 nm) and by charring with sulfuric acid in ethanol (20:80, *v*/*v*), or potassium permanganate solution [preparation: 1.5 g of KMnO_4_, 10 g of K_2_CO_3_, 1.25 mL of 10% sol. of NaOH in 200 mL of H_2_O], or vanillin solution [preparation: 15 g of vanillin in 250 mL of ethanol and 2.5 mL of conc. H_2_SO_4_]. Flash column chromatography was performed using an Isolera^TM^ automated flash purification system (Biotage AB, Uppsala, Sweden) equipped with KP-Sil 10–100 g flash cartridges (Biotage AB, Uppsala, Sweden) for normal phase separations and C_18_ 25 μm flash cartridges (Biotage AB, Uppsala, Sweden) for reverse phase separations. Optical rotations were measured at 589 nm and 20 °C on a digital polarimeter with a path length of 10 cm. ^1^H and ^13^C NMR spectra of all compounds were recorded using at 400 MHz and 600 MHz instruments unless otherwise specified and assignments made with the help of COSY, HMBC, and HSQC spectra. ESI-HRMS were recorded using a time-of-flight mass spectrometer fitted with an electrospray source. Copies of ^1^H and ^13^C NMR spectra for all new compounds are provided in the Supplementary Material.

### 4.2. 5-Azido-5-deoxy-1,2-O-isopropylidene-α-D-xylofuranose (8)

The title compound was prepared according to literature procedure [49]. Accordingly, a stirred solution of 1,2-*O*-isopropylidine-*α*-D-xylofuranose **7** (5 g, 26.29 mmol, 1 equiv) in anhydrous dichloromethane (100 mL) was cooled to 0 °C (crushed ice bath) and treated with anhydrous pyridine (4.89 mL, 60.46 mmol, 2.3 equiv) under argon atmosphere. Then, a solution of SOCl_2_ (2.19 mL, 30.23 mmol, 1.15 equiv) in anhydrous dichloromethane (20 mL) was added dropwise at 0 °C over a period of 20 min. The resulting yellowish solution was stirred at 0 °C for 2 h, and the reaction progress was monitored by GC-MS assay. Upon completion of the reaction, a solution was transferred to a separatory funnel and washed with water (3 × 50 mL). The DCM layer was dried over Na_2_SO_4_, filtered off concentrated under reduced pressure keeping the water bath temperature below 30 °C to avoid product decomposition. The yellow residue was dissolved in anhydrous DMF (50 mL) and NaN_3_ (5.12 g, 78.9 mmol, 3 equiv) was added. The resulting brown suspension was heated at 110 °C with stirring for 18 h, then it was cooled to ambient temperature and all volatiles were removed *in vacuo*. The residue was dissolved in EtOAc (100 mL) and washed with water (100 mL). The water layer was back-extracted with Et_2_O (3 × 100 mL). The combined EtOAc and Et_2_O extracts were washed with water (100 mL) to remove residual DMF and inorganic salts, then dried over Na_2_SO_4_, filtered and concentrated under reduced pressure. The resulting yellow oily residue was purified on Biotage SNAP KP-Sil 50 g silica cartridge (gradient elution from 100% petroleum ether (PE) to 45% EtOAc/PE) to give **8** (4.85 g, 86%) as a colorless sticky mass. **^1^H NMR** (400 MHz, CDCl_3_, ppm) δ 5.95 (d, *J =* 3.7 Hz, 1H), 4.52 (d, *J =* 3.7 Hz, 1H), 4.31–4.23 (m, 2H), 3.66–3.57 (m, 2H), 2.22 (d, *J =* 5.2 Hz, 1H), 1.50 (s, 3H), 1.32 (d, *J =* 0.8 Hz, 3H). The ^1^H NMR spectrum was in agreement with that reported in the literature [50].

### 4.3. 5-Azido-5-deoxy-1,2-O-isopropylidene-α-D-erythro-pentofuranos-3-ulose (9)

A stirred colorless solution of xylofuranose **8** (0.80 g, 3.72 mmol) in anhydrous dichloromethane (10 mL) was cooled to 0 °C (crushed ice bath), and Dess-Martin periodinane (2.05 g, 4.83 mmol, 1.3 equiv) was added under argon atmosphere. After stirring at 0 °C for 20 min, the white suspension was warmed to ambient temperature and stirred for additional 2 h. The reaction progress was monitored by GC-MS assay. Upon completion of the reaction, the yellowish suspension was diluted with 10% aqueous sodium thiosulfate solution (30 mL) and transferred to a separation funnel. Layers were separated and the organic layer was washed with saturated aqueous NaHCO_3_ solution (50 mL), brine, dried over Na_2_SO_4_ and filtered. Concentration under reduced pressure afforded yellowish residue that was purified on Biotage SNAP KP-Sil 25 g silica cartridge (gradient elution from 100% PE to 40% EtOAc/PE) to give **9** (0.76 g, 96%) as a colorless oil. **^1^H NMR** (400 MHz, CDCl_3_, ppm) δ 6.15 (d, *J =* 4.4 Hz 1H), 4.50 (td, *J =* 3.3, 1.1 Hz, 1H), 4.38 (dd, *J =* 4.4, 1.1 Hz, 1H), 3.68 (dd, *J =* 13.2, 3.3 Hz, 1H), 3.54 (dd, *J =* 13.2, 3.3 Hz, 1H), 1.49 (s, 3H), 1.43 (s, 3H). The ^1^H NMR spectrum was in agreement with that reported in the literature [51].

### 4.4. 2-(5-Bromopentyloxy)-tetrahydro-2H-pyran (10)

To a stirred solution of 5-bromo-1-pentanol (7.0 mL, 57.83 mmol) in anhydrous DCM (75 mL) was added *p*-toluenesulfonic acid hydrate (1.10 g, 5.78 mmol, 0.1 equiv) under argon atmosphere. The resulting clear solution was cooled to 0 °C (crushed ice bath) and 3,4-dihydro-2H-pyran (7.9 mL, 86.74 mmol, 1.5 equiv) was added dropwise over a period of 20 min. The resulting colorless solution was warmed to ambient temperature and stirred for 18 h. The reaction progress was monitored by GC-MS assay. After complete conversion, the reaction mixture was diluted with water (100 mL), layers were separated and the aqueous layer was back-extracted with DCM (3 × 50 mL). The combined DCM extracts were washed with brine (100 mL), dried over Na_2_SO_4_, filtered and concentrated under reduced pressure. The yellowish oily residue was purified on Biotage SNAP KP-Sil 100 g silica cartridge (gradient elution from 100% PE to 5% EtOAc/PE) to give **10** (12.74 g, 88%) as a colorless oil. **^1^H NMR** (400 MHz, CDCl_3_, ppm) δ 4.64–4.52 (m, 1H), 3.91–3.83 (m, 1H), 3.79–3.71 (m, 1H), 3.53–3.47 (m, 1H), 3.45–3.36 (m, 3H), 1.93–1.79 (m, 3H), 1.76–1.47 (m, 9H). The ^1^H NMR spectrum was in agreement with that reported in the literature [28].

### 4.5. (5-((Tetrahydro-2H-pyran-2-yl)oxy)pentyl)magnesium bromide (11)

An oven-dried round-bottom two neck flask equipped with magnetic stir-bar was cooled to ambient temperature under an argon atmosphere. Magnesium turnings (2.66 g, 101.13 mmol, 2 equiv) were placed in the flask and activated by intensive stirring for 12 h under argon atmosphere at ambient temperature. Anhydrous THF (5 mL) was then added, a reflux condenser was mounted and the slurry was heated at 60 °C (water bath) under an argon atmosphere. 1,2-Dibromomethane (435 µL, 0.1 equiv) was added dropwise under argon, and after gas evolution ceased, a solution of bromide **10** (12.74 g, 50.57 mmol, 1 equiv) in anhydrous THF (50 mL) was added dropwise at 60 °C over a period of 45 min. The resulting gray suspension was stirred at ambient temperature for additional 3 h, then stirring was turned off and the suspension was left undisturbed overnight under argon atmosphere. The supernatant was carefully transferred via *cannula* to an oven-dried round-bottom flask and diluted with anhydrous THF (45 mL). Concentration of the Grignard reagent **11** was determined to be 0.38 M by titration with menthol and 1,10-phenanthroline [52].

### 4.6. 5-Azido-5-deoxy-1,2-O-isopropylidene-3-C-(6-(5-((tetrahydro-2H-pyran-2-yl)oxy)pentyl)-α-D-ribofuranose (12)

Grignard reagent **11** (0.38 M solution in THF, 34.0 mL, 13.6 mmol, 2 equiv), ZnCl_2_ (0.7 M solution in anhydrous THF, 3.9 mL, 2.7 mmol, 0.4 equiv) and LiCl (0.5 M solution in anhydrous THF, 27.2 mL, 13.6 mmol, 2 equiv) were mixed and the resulting gray solution was stirred at ambient temperature for 30 min, whereupon it was cooled to −78 °C (dry ice/acetone bath). A solution of ketone **9** (1.45 g, 6.80 mmol) in anhydrous THF (2.5 mL) was added rapidly at a rate to keep temperature below −60 °C. The resulting yellow suspension was stirred at −78 °C for 1 h, warmed to ambient temperature over a period of 30 min and quenched with saturated aqueous NH_4_Cl solution (25 mL). The yellow slurry was transferred to a separation funnel, diluted with water (100 mL), and the product was back-extracted with EtOAc (3 × 50 mL). The organic extracts were combined, dried over Na_2_SO_4_ and filtered off. The solvent was evaporated under reduced pressure and the yellow residue was purified on a KP-Sil 50 g silica cartridge (gradient elution from 10% EtOAc/PE to 50% EtOAc/PE) to give **12** (1.88 g, 72%) as a yellowish viscous oil; analytical TLC on silica gel, 1:1 EtOAc/PE, *R_f_* = 0.60. ${\left[\alpha \right]}_{D}^{20}$ +21.5 (*c* 0.40, CHCl_3_). **^1^H NMR (400 MHz, CDCl_3_, ppm)** δ 5.78 (d, *J =* 3.9 Hz, 1H), 4.59–4.55 (m, 1H), 4.31 (d, *J =* 3.9 Hz, 1H), 3.90 (dd, *J =* 7.0, 4.8 Hz, 1H), 3.85 (dt, *J =* 7.5, 3.6 Hz, 1H), 3.74 (dtd, *J =* 9.2, 7.0, 2.4 Hz, 1H), 3.53–3.46 (m, 1H), 3.45–3.35 (m, 3H), 2.61 (s, 1H), 1.88–1.78 (m, 1H), 1.76–1.69 (m, 1H), 1.64–1.48 (m, 8H), 1.58 (s, 3H), 1.45–1.36 (m, 4H), 1.37 (s, 3H). **^13^C{^1^H} NMR (101 MHz, CDCl_3_, ppm)** δ 112.7, 103.8, 99.0, 81.7, 80.5, 79.0, 67.6, 62.5, 49.7, 30.9, 30.7, 29.8, 27.0, 26.7, 26.6, 25.6, 22.9, 19.8. **HRMS** (ESI/Q-TOF) *m/z*: [M-acetone + H]^+^ Calculated C_15_H_26_N_3_O_5_: 328.3848. Found: 328.3822.

### 4.7. 5-Azido-5-deoxy-1,2-O-isopropylidene-3-C-(6-(5-((tetrahydro-2H-pyran-2-yl)oxy)pentyl)-3-O-benzoyl-α-D-ribofuranose (13)

Benzoic anhydride (2.13 g, 9.42 mmol, 3 equiv) and DMAP (192 mg, 1.57 mmol, 0.5 equiv) were added to a stirred solution of tertiary alcohol **12** (1.21 g, 3.12 mmol) in anhydrous pyridine (15 mL) at 0 °C (crushed ice bath). The resulting yellowish solution was heated at 100 °C for 18 h. After cooling to ambient temperature, volatiles were evaporated under reduced pressure. The yellow residue was diluted with EtOAc (50 mL) and washed with saturated aqueous NaHCO_3_ solution (3 × 50 mL). The organic layer was dried over Na_2_SO_4_, filtered and concentrated under reduced pressure. The yellow oily residue was purified on KP-Sil 50 g silica cartridge (gradient elution from 10% EtOAc/PE to 50% EtOAc/PE) to give **13** (1.43 g, 93%) as a yellowish sticky mass; analytical TLC on silica gel, 1:1 EtOAc/PE, *R_f_* = 0.63. ${\left[\alpha \right]}_{D}^{20}$ +22.8 (*c* 0.57, CHCl_3_).**^1^H NMR** (400 MHz, CDCl_3_, ppm) δ 8.04–7.98 (m, 2H), 7.62–7.55 (m, 1H), 7.49–7.42 (m, 2H), 5.81 (d, *J =* 3.7 Hz, 1H), 4.94 (d, *J =* 3.7 Hz, 1H), 4.55–4.49 (m, 1H), 4.37 (dd, *J =* 7.2, 4.8 Hz, 1H), 3.82 (ddd, *J =* 11.1, 7.6, 3.3 Hz, 1H), 3.74–3.65 (m, 1H), 3.63–3.57 (m, 2H), 3.47 (dt, *J =* 10.4, 5.0 Hz, 1H), 3.34 (dt, *J =* 9.5, 6.3 Hz, 1H), 2.03–1.87 (m, 2H), 1.83–1.64 (m, 2H), 1.60–1.46 (m, 9H), 1.45–1.29 (m, 7H).**^13^C{^1^H} NMR** (101 MHz, CDCl_3_, ppm) δ 165.0, 133.4, 130.1, 129.9, 128.6, 112.9, 104.0, 99.0, 85.3, 83.1, 80.0, 67.4, 62.5, 50.2, 30.9, 30.4, 29.5, 27.0, 26.9, 26.8, 25.6, 23.6, 19.8. **HRMS** (ESI/Q-TOF) *m/z*: [M + Na]^+^ Calculated C_25_H_35_N_3_O_7_Na: 512.2373. Found: 512.2394.

### 4.8. 5-Azido-5-deoxy-1,2-O-isopropylidene-3-C-(5-hydroxypentyl)-3-O-benzoyl-α-D-ribofuranose (14)

Tetrabutylammonium tribromide (80 mg, 0.17 mmol, 0.1 equiv) was added to a stirred solution of THP-protected alcohol **13** (810 mg, 1.65 mmol) in MeOH (15 mL) at ambient temperature. The resulting orange solution was stirred for 3 h, then acetone (25 mL) was added and the resulting solution was stirred for additional 15 min. After the volatiles were evaporated under reduced pressure, the orange oily residue was diluted with EtOAc (50 mL) and washed with saturated aqueous NaHCO_3_ solution (3 × 50 mL). The organic layer was dried over Na_2_SO_4_, filtered and concentrated under reduced pressure. The orange oily residue was purified on KP-Sil 50 g silica cartridge (gradient elution from 20% EtOAc/PE to 60% EtOAc/PE) to give **14** (655 mg, 98%) as a yellowish viscous oil; analytical TLC on silica gel, 1:1 EtOAc/PE, *R_f_* = 0.30. ${\left[\alpha \right]}_{D}^{20}$ +70.2 (*c* 0.38, CHCl_3_). **^1^H NMR** (400 MHz, CDCl_3_, ppm) δ 8.03–7.98 (m, 2H), 7.62–7.55 (m, 1H), 7.49–7.42 (m, 2H), 5.81 (d, *J =* 3.7 Hz, 1H), 4.94 (d, *J =* 3.7 Hz, 1H), 4.37 (dd, *J =* 7.3, 4.8 Hz, 1H), 3.63–3.56 (m, 4H), 2.03–1.88 (m, 2H), 1.58–1.51 (m, 2H), 1.49 (s, 3H), 1.43–1.29 (m, 8H). **^13^C{^1^H} NMR** (101 MHz, CDCl_3_, ppm) δ 165.0, 133.4, 130.0, 129.9, 128.6, 112.9, 104.0, 85.2, 83.1, 79.9, 62.8, 50.1, 32.4, 30.4, 26.8, 26.7, 26.3, 23.6. **HRMS** (ESI/Q-TOF) *m/z*: [M + Na]^+^ Calculated C_20_H_27_N_3_O_6_Na: 428.1798. Found: 428.1786.

### 4.9. 5-Azido-5-deoxy-1,2-O-isopropylidene-3-C-(5-oxopentyl)-3-O-benzoyl-α-D-ribofuranose (15)

A stirred solution of alcohol **14** (610 mg, 1.50 mmol) in anhydrous DCM (10 mL) was cooled to 0 °C (crushed ice bath) under argon atmosphere and treated with Dess-Martin periodinane (830 mg, 1.96 mmol, 1.3 equiv), followed by few drops of NEt_3_. After stirring at 0 °C for 20 min, the white suspension was warmed to ca. 10 °C and stirred at this temperature for additional 2–3 h. The progress of the reaction was followed by TLC and UPLC assays. After completion of the reaction, the white suspension was diluted with 10% aqueous sodium thiosulfate solution (25 mL) and layers were separated. The organic layer was washed with saturated NaHCO_3_ solution (50 mL), brine, dried over Na_2_SO_4_ and filtered. Removal of volatiles under reduced pressure afforded pale yellow oily residue that was purified on KP-Sil 10 g silica cartridge (gradient elution from 20% to 50% EtOAc/PE) to afford **15** (572 mg, 94%) as a colorless oil; analytical TLC on silica gel, 1:1 EtOAc/PE, *R_f_* = 0.51. ${\left[\alpha \right]}_{D}^{20}$ +69.1 (*c* 0.42, CHCl_3_). **^1^H NMR** (400 MHz, CDCl_3_, ppm) δ 9.73 (t, *J =* 1.4 Hz, 1H), 8.03–7.98 (m, 2H), 7.63–7.56 (m, 1H), 7.48–7.44 (m, 2H), 5.81 (d, *J =* 3.7 Hz, 1H), 4.93 (d, *J =* 3.7 Hz, 1H), 4.36 (dd, *J =* 6.9, 5.1 Hz, 1H), 3.63–3.55 (m, 2H), 2.46–2.40 (m, 2H), 2.06–1.93 (m, 2H), 1.67–1.58 (m, 2H), 1.49 (s, 3H), 1.40–1.32 (m, 5H). **^13^C{^1^H} NMR** (101 MHz, CDCl_3_, ppm) δ 201.8, 165.0, 133.5, 129.9, 129.9, 128.6, 113.0, 103.9, 85.1, 83.1, 79.8, 50.0, 43.5, 30.3, 26.8, 23.3, 22.4. **HRMS** (ESI/Q-TOF) *m/z*: [M-acetone + H]^+^ Calculated C_17_H_20_N_3_O_5_: 346.1403. Found: 346.1414.

### 4.10. 5-Azido-5-deoxy-1,2-O-isopropylidene-3-C-(5-(dimethylamino)pentyl)-3-O-benzoyl-α-D-ribofuranose (16)

To a solution of aldehyde **15** (570 mg, 1.41 mmol) in anhydrous THF (15 mL) at ambient temperature were added Me_2_NH (2 M solution in THF, 2.1 mL, 4.2 mmol, 3 equiv) and glacial acetic acid (81 µL, 1.41 mmol, 1 equiv). The resulting yellow solution was stirred for 1 h then cooled to 0 °C (crushed ice bath), and NaBH(OAc)_3_ (449 mg, 2.12 mmol, 1.5 equiv) was added in 3 portions. The yellow suspension was stirred at 0 °C for 2 h whereupon water (25 mL) and saturated aqueous NaHCO_3_ solution (25mL) were added. The resulting cloudy solution was extracted with EtOAc (3 × 30 mL), combined organic extracts were dried over Na_2_SO_4_, filtered and concentrated under reduced pressure. The yellow oily residue was purified on KP-Sil 10 g silica cartridge (gradient elution from 100% EtOAc to 2% NEt_3_ in EtOAc) to give **16** (440 mg, 72%) as a yellow oil; analytical TLC on silica gel, 30% MeOH in DCM, *R_f_* = 0.33. ${\left[\alpha \right]}_{D}^{20}$ +48.0 (*c* 0.39, CHCl_3_). **^1^H NMR** (400 MHz, CDCl_3_, ppm) δ 8.03–7.99 (m, 2H), 7.61–7.56 (m, 1H), 7.49–7.43 (m, 2H), 5.81 (d, *J =* 3.7 Hz, 1H), 4.94 (d, *J =* 3.7 Hz, 1H), 4.36 (dd, *J =* 7.4, 4.8 Hz, 1H), 3.64–3.55 (m, 2H), 2.21–2.16 (m, 8H), 2.05–1.88 (m, 2H), 1.49 (s, 3H), 1.45–1.38 (m, 2H), 1.37–1.25 (m, 7H). **^13^C{^1^H} NMR** (101 MHz, CDCl_3_, ppm) δ 165.0, 133.4, 130.1, 129.9, 128.6, 112.9, 104.0, 85.3, 83.1, 80.0, 59.7, 50.2, 45.6, 30.4, 28.0, 27.5, 26.8, 26.8, 26.7, 23.7. **HRMS** (ESI/Q-TOF) *m/z*: [M + H]^+^ Calculated C_22_H_33_N_4_O_5_: 433.2451. Found: 433.2462.

### 4.11. 1,2-Di-O-acetyl-5-azido-5-deoxy-3-C-(5-(dimethylamino)pentyl)-3-O-benzoyl-α/β-D-ribofuranose (17)

A stirred solution of 1,2-*O*-isopropylidene-protected ribofuranose **16** (1.1 g, 2.54 mmol) in glacial acetic acid (20 mL) and Ac_2_O (9.6 mL, 102 mmol, 40 equiv) was cooled to 0 °C (crushed ice bath). Concentrated H_2_SO_4_ (68 µL, 1.27 mmol, 0.5 equiv) was added dropwise at 0 °C. The yellow solution was warmed to ambient temperature and stirred for 18 h, whereupon volatiles were removed under reduced pressure keeping the water bath temperature below 30 °C. The brown oily residue was diluted with DCM (50 mL) and water (20 mL), cooled to 0 °C (crushed ice bath) and pH of aqueous layer was adjusted to the neutral by addition of saturated aqueous NaHCO_3_ solution. The mixture was transferred to a separation funnel, layers were separated and the aqueous layer was extracted with EtOAc (3 × 30 mL). The combined organic extracts were dried over Na_2_SO_4_, filtered and concentrated under reduced pressure. The resulting brown oily residue was purified on KP-Sil 25 g silica cartridge (gradient elution from 100% EtOAc to 2% NEt_3_ in EtOAc) to give **17** (1.01 g, 83%; 3:2 α:β mixture of anomers) as a yellow oil; analytical TLC on silica gel, 5% NEt_3_ in EtOAc, *R_f_* = 0.40. **^1^H NMR** (400 MHz, CDCl_3_, ppm) δ 8.09–7.94 (m, 2H, both anomers), 7.64–7.57 (m, 1H, both anomers), 7.51–7.43 (m, 2H, both anomers), 6.49 (d, *J =* 4.6 Hz, 0.6H, major anomer), 6.23 (d, *J =* 2.4 Hz, 0.4H, minor anomer), 5.61 (d, *J =* 2.4 Hz, 0.4H, minor anomer), 5.32 (d, *J =* 4.6 Hz, 0.6H, major anomer), 4.85 (dd, *J =* 4.8, 3.5 Hz, 0.6H, major anomer), 4.74 (dd, *J =* 6.6, 3.3 Hz, 0.4H, minor anomer), 3.84–3.81 (m, 0.4H, minor anomer), 3.81–3.77 (m, 0.6H, major anomer), 3.60–3.56 (m, 0.6H, major anomer), 3.56–3.53 (m, 0.4H, minor anomer), 2.63–2.45 (m, 1H, both anomers), 2.42–2.30 (m, 1H, both anomers), 2.18 (s, 6H, both anomers), 2.15 (s, 1.8H, major anomer), 2.14 (s, 1.2H, minor anomer), 2.13 (s, 1.2H, minor anomer), 2.04 (s, 1.8H, major anomer), 1.91–1.73 (m, 1H, both anomers), 1.47–1.34 (m, 3H, both anomers), 1.32–1.23 (m, 4H, both anomers). **^13^C{^1^H NMR** (101 MHz, CDCl_3_, ppm; 3:2 α:β mixture of anomers) δ 169.8, 169.7, 169.7, 169.7, 165.3, 133.8, 133.8, 130.2, 129.9, 129.9, 129.8, 129.8, 128.9, 128.8, 128.7, 98.8, 93.2, 86.8, 85.5, 84.5, 84.3, 79.3, 76.4, 58.4, 51.9, 51.0, 50.9, 43.9, 31.9, 30.5, 27.1, 25.2, 23.2, 22.8, 22.7, 21.3, 21.1, 21.0, 20.8. **HRMS** (ESI/Q-TOF) *m/z*: [M + H]^+^ Calculated C_23_H_33_N_4_O_7_: 477.2349. Found: 477.2315.

### 4.12. 5-O-[5′′′-Azido-5′′′-deoxy-2′′′-O-acetyl-3-C-(5-(dimethylamino)pentyl)-3-O-benzoyl-α/β-D-ribofuranosyl]-6,2′′,3′′,6′′-tetra-O-benzoyl-1,3,2′,4′′-tetraazido-1,3,2′,4′′-tetra(desamino)-6′,7′-oxazolidino-apramycin trifluoroacetate (19)

A mixture of ribofuranose **17** (100 mg, 0.21 mmol) and protected apramycin **18** [20] (228 mg, 0.21 mmol, 1 equiv) were co-evaporated with anhydrous toluene (5 mL) on a rotary evaporator three times, followed by overnight vacuum drying. Anhydrous DCM (5 mL) was added under an argon atmosphere, the resulting yellow solution was cooled to −10 °C (crushed ice/NaCl bath) and BF_3_•OEt_2_ (331 µL, 1.26 mmol, 6 equiv) was added. The resulting yellow solution was stirred at 0 °C for 48 h, and the reaction progress was monitored by UPLC-MS assay. Upon complete conversion of ribofuranose **17**, the reaction was quenched with NEt_3_ (0.3 mL, 2.10 mmol, 10 equiv) at 0 °C and the resultant mixture was diluted with EtOAc (20 mL). The organic phase was washed with aqueous saturated NaHCO_3_ solution (50 mL), and the aqueous layer was back-extracted with DCM (3 × 30 mL). The combined EtOAc and DCM extracts were dried over Na_2_SO_4_, filtered and concentrated under reduced pressure. The brown residue was purified by reversed-phase preparative HPLC (column: XBridge^®^ Prep C_18_ 5 μm OBD^TM^, 30 x 100 mm, Waters Corporation Ltd, Dublin, Ireland) using gradient elution from 60% MeCN in 0.1% aqueous TFA solution to 95% MeCN in 0.1% aqueous TFA solution) to give **19α** (32 mg, 10%, white powder) and **19β** (60 mg, 20%, white powder); analytical TLC on silica gel, 5% NEt_3_ in EtOAc, *R_f_* = 0.40.

**19α-anomer:**${\left[\alpha \right]}_{D}^{20}$ +72.3 (*c* 0.25, CHCl_3_).**^1^H NMR** (400 MHz, CDCl_3_, ppm) δ 12.7–11.9 (br s, 1H), 8.12–8.05 (m, 4H), 8.05–8.00 (m, 2H), 8.00–7.94 (m, 2H), 7.74–7.67 (m, 2H), 7.63–7.34 (m, 15H), 6.01 (t, *J* = 10.1 Hz, 1H), 5.73 (d, *J =* 3.1 Hz, 1H), 5.71 (d, *J =* 3.6 Hz, 1H), 5.34 (d, *J =* 1.8 Hz, 1H), 5.24 (t, *J =* 9.8 Hz, 1H), 5.19 (d, *J =* 1.8 Hz, 1H), 5.17 (dd, *J =* 10.5, 3.6 Hz, 1H), 4.78 (d, *J =* 6.0 Hz, 1H), 4.75 (dd, *J =* 6.4, 3.6 Hz, 1H), 4.67 (dd, *J =* 12.0, 2.3 Hz, 1H), 4.62 (dd, *J =* 12.2, 4.9 Hz, 1H), 4.31 (dd, *J =* 7.7, 2.0 Hz, 1H), 4.13–4.03 (m, 3H), 3.99–3.93 (m, 1H), 3.89 (t, *J =* 10.1 Hz, 1H), 3.81 (t, *J =* 9.3 Hz, 1H), 3.75 (t, *J =* 6.3 Hz, 1H), 3.63–3.55 (m, 1H), 3.55–3.47 (m, 2H), 3.47–3.38 (m, 1H), 3.08–2.66 (m, 12H), 2.43 (dt, *J =* 12.8, 4.2 Hz, 1H), 2.21–2.10 (m, 1H), 1.79–1.66 (m, 3H), 1.64–1.54 (m, 4H), 1.40–1.14 (m, 6H).**^13^C{^1^H} NMR** (101 MHz, CDCl_3_, ppm) δ 169.0, 165.9, 165.7, 165.4, 164.8, 164.5, 157.0, 133.6, 133.5, 133.4, 133.3, 129.9, 129.8, 129.6, 129.5, 129.3, 129.1, 129.1, 128.8, 128.6, 128.6, 128.5, 128.4, 128.3, 105.8, 100.7, 96.2, 95.6, 83.9, 82.7, 80.5, 78.5, 75.9, 75.2, 71.8, 71.0, 70.2, 69.7, 66.4, 65.8, 63.1, 60.7, 60.1, 59.1, 58.2, 57.4, 55.4, 51.2, 42.7, 42.5, 31.2, 30.2, 29.5, 27.9, 26.2, 23.6, 22.3, 19.9. **HRMS** (ESI/Q-TOF) *m/z*: [M + H]^+^ Calculated C_71_H_76_N_17_O_21_: 1502.5402. Found: 1502.5424.

**19β-anomer:**${\left[\alpha \right]}_{D}^{20}$ +61.3 (*c* 0.31, CHCl_3_).**^1^H NMR** (400 MHz, CDCl_3_, ppm) δ 11.2–10.9 (br s, 1H), 8.10–8.06 (m, 2H), 8.05–8.00 (m, 2H), 7.98–7.91 (m, 4H), 7.71–7.65 (m, 2H), 7.65–7.59 (m, 1H), 7.56–7.50 (m, 4H), 7.47–7.40 (m, 3H), 7.40–7.30 (m, 5H), 7.25–7.21 (m, 2H), 6.00 (t, *J =* 10.1 Hz, 1H), 5.75–5.67 (m, 2H), 5.43 (s, 1H), 5.30 (s, 1H), 5.24 (t, *J =* 9.9 Hz, 1H), 5.16 (dd, *J =* 10.4, 3.6 Hz, 1H), 4.80 (d, *J =* 5.6 Hz, 1H), 4.76 (dd, *J =* 6.9, 3.6 Hz, 1H), 4.68 (dd, *J =* 12.2, 2.4 Hz, 1H), 4.62 (dd, *J =* 12.2, 4.9 Hz, 1H), 4.22 (dd, *J =* 8.6, 2.2 Hz, 1H), 4.15–4.06 (m, 3H), 3.89 (t, *J =* 10.1 Hz, 1H), 3.84–3.79 (m, 1H), 3.78–3.73 (m, 2H), 3.57–3.49 (m, 3H), 3.45 (ddd, *J =* 12.4, 10.0, 4.8 Hz, 1H), 3.07–2.99 (m, 3H), 2.93 (s, 3H), 2.91–2.82 (m, 6H), 2.45 (dt, *J =* 13.0, 4.5 Hz, 1H), 2.10–1.99 (m, 1H), 1.79–1.64 (m, 7H), 1.64–1.53 (m, 2H), 1.38–1.24 (m, 4H).**^13^C{^1^H}** NMR (101 MHz, CDCl_3_, ppm) δ 169.1, 166.3, 166.1, 165.8, 165.3, 164.5, 157.4, 133.9, 133.8, 133.8, 133.7, 133.5, 130.1, 130.0, 129.9, 129.9, 129.7, 129.5, 129.0, 128.9, 128.9, 128.8, 128.7, 128.7, 128.6, 128.5, 128.4, 106.9, 100.5, 96.4, 95.9, 83.4, 83.3, 80.9, 79.3, 76.7, 75.3, 72.0, 71.4, 70.6, 70.0, 66.6, 66.0, 63.5, 61.1, 60.4, 59.3, 58.5, 58.1, 56.0, 51.3, 43.3, 43.2, 31.5, 30.4, 29.5, 28.5, 26.5, 24.1, 22.8, 20.9. **HRMS** (ESI/Q-TOF) *m/z*: [M + H]^+^ Calculated C_71_H_76_N_17_O_21_: 1502.5402. Found: 1502.5435.

### 4.13. 5-O-[5′′′-Amino-5′′′-deoxy-3-C-(5-(dimethylamino)pentyl)-β-D-ribofuranosyl]-apramycin heptaacetate (5)

A mixture of apramycin derivative **19β** (80 mg, 0.05 mmol) in dioxane (1 mL) and NaOH (2 M aqueous solution, 240 µL, 0.48 mmol, 9 equiv) was heated at 80 °C for 24 h. The reaction progress was monitored by UPLC-MS assay. Upon complete conversion, the colorless solution was cooled to 0 °C (crushed ice bath) and dry ice was added portion-wise until pH 8. The resulting solution was concentrated to dryness under reduced pressure. The white residue was dissolved in a mixture of dioxane/deionized water/glacial acetic acid (1:1:1, 3 mL) and 10% Pd on carbon (85 mg, 0.08 mmol, 1.5 equiv) was added. The black suspension was vigorously stirred under 3 atm hydrogen pressure at ambient temperature for 18 h, and the progress of the reaction was monitored by UPLC-MS assay. Upon complete conversion, the black suspension was filtered through the pad of Celite*^®®®^*, and the filter cake was washed with 1:1 AcOH:water mixture. Combined filtrates were evaporated under reduced pressure, and the sticky oil was purified by reversed-phase preparative HPLC (column: XBridge^®^ BEH Prep OBD^TM^ Amide, 5 μm, 30 x 100 mm, Waters Corporation Ltd, Dublin, Ireland) using gradient elution from 95:5 A:B to 10:90 A:B (eluent A: 0.1% solution of AcOH in MeCN; eluent B: 0.1% solution of AcOH in water). The product-containing fractions (identified by ESI-MS) were combined and concentrated. Glacial acetic acid was added to the sticky oily residue, and subsequent trituration with MeCN afforded the heptaacetate salt of **5** as a white amorphous solid (32 mg, 50% yield). ${\left[\alpha \right]}_{D}^{20}$ +64.4 (*c* 0.104, H_2_O). **^1^H NMR** (600 MHz, D_2_O, ppm) δ 5.72 (d, *J =* 3.9 Hz, 1H), 5.41 (d, *J =* 3.9 Hz, 1H), 5.35 (d, *J =* 4.3 Hz, 1H), 5.12 (d, *J =* 8.5 Hz, 1H), 4.49 (t, *J =* 2.8 Hz, 1H), 4.09–4.04 (m, 2H), 4.02 (d, *J =* 4.1 Hz, 1H), 3.90 (t, *J =* 9.3 Hz, 1H), 3.88–3.83 (m, 2H), 3.83 (t, *J* = 10.1 Hz, 1H), 3.79–3.72 (m, 2H), 3.69 (dd, *J =* 12.4, 4.5 Hz, 1H), 3.65–3.55 (m, 3H), 3.38 (ddd, *J =* 13.7, 9.9, 4.1 Hz, 1H), 3.28 (dd, *J* = 8.5, 2.8 Hz, 1H), 3.24 (td, *J =* 12.5, 4.2 Hz, 1H), 3.21–3.12 (m, 2H), 3.05–2.98 (m, 3H), 2.75 (s, 6H), 2.68 (s, 3H), 2.33 (dt, *J =* 12.5, 4.2 Hz, 1H), 2.27 (dt, *J =* 9.0, 4.6 Hz, 1H), 2.01–1.92 (m, 1H), 1.82 (s, 21H), 1.73 (q, *J =* 12.7 Hz, 1H), 1.66–1.54 (m, 3H), 1.49–1.35 (m, 2H), 1.33–1.22 (m, 3H). **^13^C{^1^H} NMR** (151 MHz, D_2_O, ppm) δ 180.2, 106.7, 93.8, 93.0, 92.1, 82.7, 81.6, 78.4, 77.8, 73.8, 71.1, 69.6, 69.0, 68.9, 67.7, 65.3, 62.0, 59.6, 58.7, 56.9, 51.3, 49.5, 48.1, 47.0, 41.8, 39.7, 31.0, 29.3, 27.9, 26.4, 25.4, 23.2, 22.3, 21.3. **HRMS** (ESI/Q-TOF) *m/z*: [M + H]^+^ Calculated C_33_H_66_N_7_O_14_: 784.4668. Found: 784.4665. **Anal.** Calcd for C_47_H_93_N_7_O_28_: C, 44.10; H, 7.98; N, 7.66. Found: C, 44.31; H, 7.75; N, 7.74.

### 4.14. 5-O-[5′′′-Amino-5′′′-deoxy-3-C-(5-(dimethylamino)pentyl)-α-D-ribofuranosyl]-apramycin hexaacetate (6)

A mixture of apramycin derivative **19α** (60 mg, 0.04 mmol) in dioxane (1 mL) and NaOH (2 M aqueous solution, 180 µL, 0.36 mmol, 9 equiv) was heated at 80 °C for 24 h. The reaction progress was monitored by UPLC-MS assay. Upon complete conversion, the colorless solution was cooled to 0 °C (crushed ice bath) and dry ice was added portion-wise until pH 7–8. The resulting solution was concentrated to dryness under reduced pressure. The white residue was dissolved in a mixture of dioxane/deionized water/glacial acetic acid (1:1:1, 3 mL) and 10% Pd on carbon (64 mg, 0.06 mmol, 1.5 equiv) was added at ambient temperature. The black suspension was vigorously stirred under 3 atm hydrogen pressure at ambient temperature for 18 h, and the progress of the reaction was monitored by UPLC-MS assay. Upon complete conversion, the black suspension was filtered through the pad of Celite^TM^ 545 (Thermo Fischer Scientific, Waltham, MA, USA), and the filter cake was washed with 1:1 AcOH:water mixture. Combined filtrates were evaporated under reduced pressure, and the sticky oil was purified by reversed-phase preparative HPLC (column: XBridge^®^ BEH Prep OBD^TM^ Amide, 5 μm, 30 x 100 mm, Waters Corporation Ltd, Dublin, Ireland) using gradient elution from 95:5 A:B to 10:90 A:B (eluent A—0.1% solution of AcOH in MeCN; eluent B—0.1% solution of AcOH in water). The product-containing fractions (identified by ESI-MS) were combined and concentrated. Glacial acetic acid was added to the sticky oily residue, and subsequent trituration with MeCN afforded the hexaacetate salt of **6** as a white amorphous solid (31 mg, 65% yield). ${\left[\alpha \right]}_{D}^{20}$ +76.9 (*c* 0.25, H_2_O). **^1^H NMR** (600 MHz, D_2_O, ppm) δ 5.60 (d, *J =* 3.5 Hz, 1H), 5.49 (d, *J =* 3.9 Hz, 1H), 5.45 (d, *J =* 4.6 Hz, 1H), 5.18 (d, *J =* 8.5 Hz, 1H), 4.53 (t, *J =* 2.8 Hz, 1H), 4.16 (dd, *J =* 11.4, 2.5 Hz, 1H), 4.07 (d, *J =* 4.5 Hz, 1H), 3.95–3.82 (m, 6H), 3.81–3.75 (m, 3H), 3.71 (dd, *J =* 9.8, 3.9 Hz, 1H), 3.50 (dt, *J =* 12.5, 4.2 Hz, 1H), 3.33–3.17 (m, 4H), 3.15–3.07 (m, 4H), 2.88 (s, 6H), 2.74 (s, 3H), 2.35–2.26 (m, 2H), 2.01–1.93 (m, 1H), 1.92 (s, 18H), 1.79–1.70 (m, 2H), 1.70–1.59 (m, 2H), 1.58–1.47 (m, 2H), 1.43–1.36 (m, 3H). **^13^C{^1^H} NMR** (151 MHz, D_2_O, ppm) δ 181.3, 107.5, 95.0, 94.5, 93.2, 83.4, 82.3, 78.0, 72.4, 70.6, 70.4, 69.7, 66.5, 66.3, 63.0, 60.5, 59.8, 57.6, 52.1, 50.5, 49.0, 48.1, 42.5, 40.5, 31.8, 30.5, 30.3, 28.2, 26.0, 23.8, 23.2, 22.0. **HRMS** (ESI/Q-TOF) *m/z*: [M + H]^+^ Calculated C_33_H_66_N_7_O_14_: 784.4668. Found: 784.4676. **Anal.** Calcd for C_45_H_89_N_7_O_26_: C, 44.18; H, 8.06; N, 8.01. Found: C, 44.07; H, 7.71; N, 7.69.

### 4.15. Cell-Free Luciferase Translation Assays

Cell-free in vitro translation inhibition assays were performed using luciferase mRNA and bacterial S30 extracts containing either wild-type bacterial or human hybrid ribosomes. In brief, firefly luciferase mRNA was transcribed in vitro using T7 RNA polymerase (Thermo Fisher Scientific, Waltham, MA, USA) using a plasmid as template in which the mammalian promoter in pGL4.14 has been replaced by theT7 bacteriophage promoter (Promega, USA). Test articles in aqueous solution containing 0.3% Tween20 were dispensed into white 96-well plates (Eppendorf, Germany) using the TECAN D300e digital dispenser (Tecan, Switzerland). The test article dispensing volume was balanced to a total of 1.5 µL by 0.3% Tween20 in water. The reaction volume was brought to 15 µL by addition of 13.5 µL Translation Master Mix comprised of bacterial S30 extract, 0.2 mM amino acid mix, 6 µg tRNA (Sigma-Aldrich, USA), 0.4 µg hFluc mRNA, 0.3 µL protease inhibitor (cOmplete, EDTA-free, Roche, USA), 12 U RNAse inhibitor (Ribolock, Thermo Fisher Scientific, Waltham, MA, USA), and 6 µL S30 premix without amino acids (Promega, USA). Dispensing and mixing of reagents was performed on ice prior to incubating the sealed plates at 37 °C. After 1 h of incubation, the reaction was stopped on ice and 75 µL of luciferase assay reagent (Promega, USA) was added to each well. Luminescence was recorded with a plate reader (BIO-TEK FLx800, Witec AG, Littau, Switzerland).

### 4.16. Antibacterial Inhibition Assays

The minimal inhibitory concentrations (MIC) of synthesized compounds were determined by broth microdilution assays according to CLSI reference methodology M07 [53] as described previously [6] and using strains described previously [54]. Clinical bacterial isolates were obtained from the diagnostic laboratories of the Institute of Medical Microbiology, University of Zurich.

## Figures and Tables

**Table 1:** Antiribosomal Activities and Selectivities (IC_50_, µM) ^a^.

	Antiribosomal Activity				Selectivity		
	wt	Mit13	A1555G	Cyt14	Mit13	A1555G	Cyt14
Apramycin **1**	0.15	114	105	158	760	700	1053
Apralog **2**	0.071	68	13	190	955	188	2669
Apralog **3**	0.13	121	87	111	909	652	837
Apralog **4**	0.031	46	22	51	1494	699	1653
Apralog **5**	0.077	72	39	116	934	510	1505
Apralog **6**	0.071	62	39	114	871	545	1599

^a^: All measurements were made in duplicate using twofold dilution series.

**Table 2:** Antibacterial Activities against Wild-Type *E. coli* and ESKAPE Pathogens (MIC, μg/mL) ^a^.

Species	MRSA	*E. coli*	*K. pneu.*	*Enterob.*	*A. baum.*	*P. aerug.* ^b^
Strain	AG038	ATCC 25922	AG215	AG290	AG309	AG220
Apramycin **1**	4	4	1–2	2–4	4	4
Apralog **2**	2–4	2	1–2	2	8	16–32
Apralog **3**	2	4	1–2	2	8	4–8
Apralog **4**	1–2	1–2	0.5–1	1	4	2
Apralog **5**	2–4	2–4	2	4	16	8–16
Apralog **6**	2–4	2–4	2	2–4	16	8–16

^a^: All values were determined in duplicate using twofold dilution series. ^b^: *P. aeruginosa* carries a chromosomal APH(3′) gene, which principally affects the 3′-hydroxy group.

**Table 3:** Activities against *E. coli* in the Presence of Specific Resistance Determinants (MIC, μg/mL) ^a^.

Resistance det	WT- Parental	APH(3′)-Ia	APH(3′)-IIa	APH(3′)-IIb	APH(3′)-VI	AAC(3)-IV	ArmA	RmtB
Strain	DH5α	EC122	EC123	EC125	EC127	EC118	EC102	EC103
Apramycin **1**	1–2	0.5–1	0.5–1	0.5–1	0.5–1	64	1	0.5–1
Apralog **2**	2	4–8	1–2	1	1–2	2–4	2	0.5–1
Apralog **4**	2	1–2	1	1	1–2	4	1–2	2
Apralog **5**	1–2	1–2	0.5–1	1	0.5–1	4	2–4	1
Apralog **6**	1–2	2	0.5–1	1	1	4	2–4	1–2

^a^: All values were determined in duplicate using twofold dilution series.

## Data Availability

All supporting spectral data may be found in the Supplementary Materials.

## Supplementary Materials

The following supporting information can be downloaded at: https://www.mdpi.com/article/10.3390/antibiotics12010025/s1, Copies of ^1^H and ^13^C NMR spectra for all new compounds.
