# Supplementary material for: Synthesis, Antibacterial and Antiribosomal Activity of the 3C-Aminoalkyl Modification in the Ribofuranosyl Ring of Apralogs (5-O-Ribofuranosyl Apramycins)
Source: Antibiotics (Basel). 2022 Dec 24;12(1):25. doi: 10.3390/antibiotics12010025 (PMC9854789; doi:10.3390/antibiotics12010025)

Supporting Information for

3-*C*-Aminoalkyl Modification in the Ribofuranosyl Ring of Apralogs (5-*O*-Ribofuranosyl Apramycins) for Enhanced Antibacterial Activity

Dmitrijs Lubriks,[a] Klara Haldimann,[b] Sven N. Hobbie,[b] Andrea Vasella,[c] Erik C. Böttger,[b,*] Edgars Suna,[a,*] and David Crich [d,e,f,*]

a) Latvian Institute of Organic Synthesis, Riga, Latvia, LV-1006
b) Institute of Medical Microbiology, University of Zurich, Gloriastrasse 30, 8006 Zürich, Switzerland
c) Organic Chemistry Laboratory, ETH Zürich, Vladimir-Prelog-Weg 1-5/10, 8093 Zürich, Switzerland
d) Department of Pharmaceutical and Biomedical Sciences, University of Georgia, 250 West Green Street, Athens, GA 30602, USA
e) Complex Carbohydrate Research Center, University of Georgia, 315 Riverbend Road, Athens, GA 30602, USA
f) Department of Chemistry, University of Georgia, 302 East Campus Road, Athens, GA 30602, USA

Table of Contents

	Spectra
5-Azido-5-deoxy-1,2-*O*-isopropylidene-*α*-D-xylofuranose **(8)**	S3
5-Azido-5-deoxy-1,2-*O*-isopropylidene-*α*-D-*erythro*-pentofuranos-3-ulose **(9)**	S4
2-(5-Bromopentyloxy)-tetrahydro-2H-pyran **(10)**	S5
5-Azido-5-deoxy-1,2-*O*-isopropylidene-3-*C*-(6-(5-((tetrahydro-2H-pyran-2-yl)oxy)pentyl)-*α*-D-ribofuranose **(12)**	S6
(5-Azido-5-deoxy-1,2-*O*-isopropylidene-3-*C*-(6-(5-((tetrahydro-2H-pyran-2-yl)oxy)pentyl)-3-*O*-benzoyl-*α*-D-ribofuranose **(13)**	S11
5-Azido-5-deoxy-1,2-*O*-isopropylidene-3-*C*-(5-hydroxypentyl)-3-*O*-benzoyl-*α*-D-ribofuranose **(14)**	S16
5-Azido-5-deoxy-1,2-*O*-isopropylidene-3-*C*-(5-oxopentyl)-3-*O*-benzoyl-*α*-D-ribofuranose **(15)**	S21
5-Azido-5-deoxy-1,2-*O*-isopropylidene-3-*C*-(5-(dimethylamino)pentyl)-3-*O*-benzoyl-*α*-D-ribofuranose **(16)**	S26
1,2-Di-*O*-acetyl-5-azido-5-deoxy-3-*C*-(5-(dimethylamino)pentyl)-3-*O*-benzoyl-*α/β*-D-ribofuranose **(17)**	S31
5-*O*-[5‴-Azido-5‴-deoxy-2‴-*O*-acetyl-3-*C*-(5-(dimethylamino)pentyl)-3-*O*-benzoyl-α/*β*-D-ribofuranosyl]-6,2″,3″,6″-tetra-*O*-benzoyl-1,3,2′,4″-tetraazido-1,3,2′,4″-tetra(desamino)-6′,7′-oxazolidino-apramycin **(19)**	S33 (**19α**)<br>S38 (**19β**)
5-*O*-[5‴-Amino-5‴-deoxy-3-*C*-(5-(dimethylamino)pentyl)-α-D-ribofuranosyl]-apramycin hepta hexa acetate **(5)**	S43
5-*O*-[5‴-Amino-5‴-deoxy-3-*C*-(5-(dimethylamino)pentyl)-*β*-D-ribofuranosyl]-apramycin hepta hexa acetate **(6)**	S49

5-Azido-5-deoxy-1,2-*O*-isopropylidene-*α*-D-xilofuranose (8)

[^{1}H NMR, 400 MHz, $CDCl_3$]

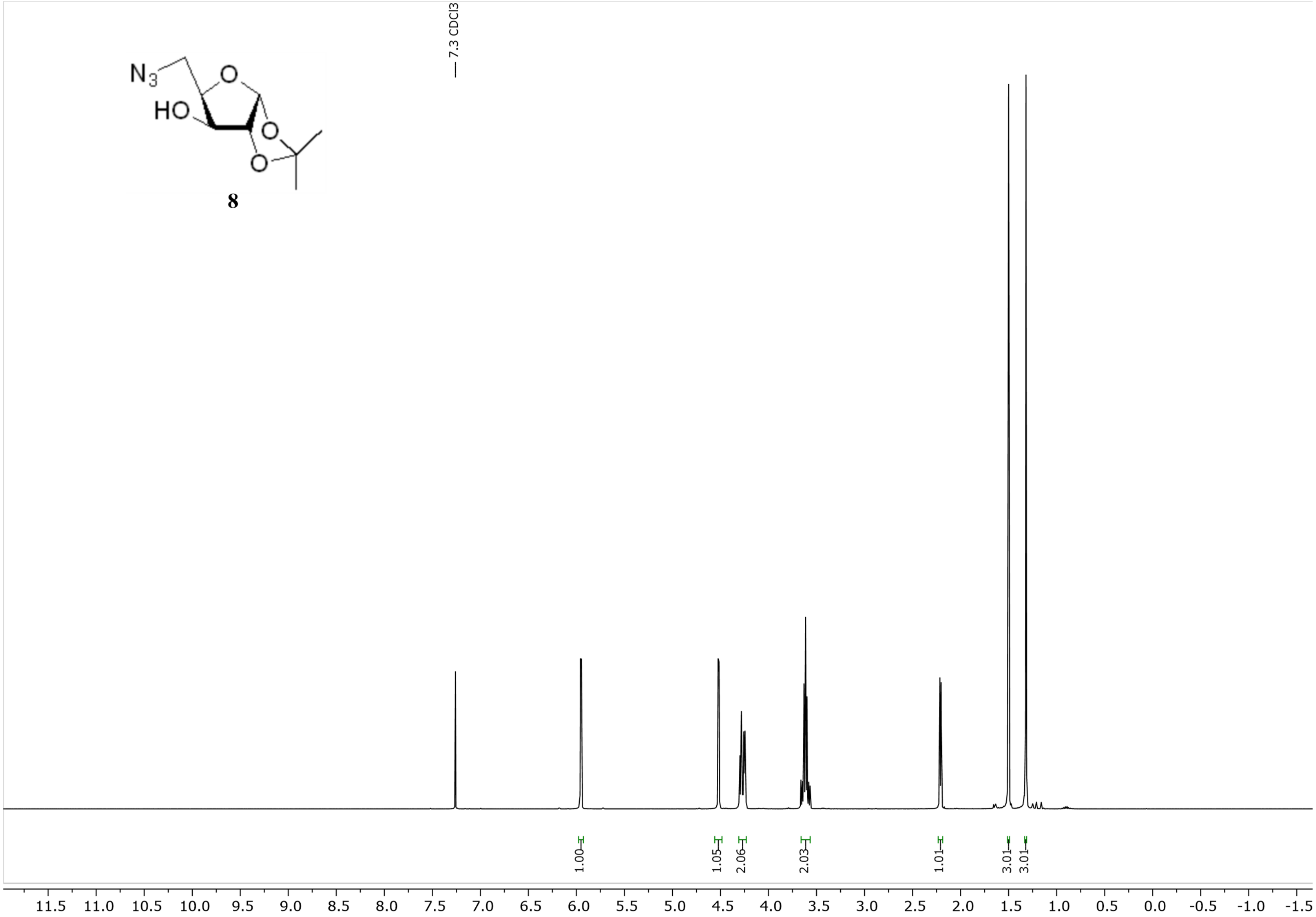

5-Azido-5-deoxy-1,2-*O*-isopropylidene-*α*-D-*erythro*-pentofuranos-3-ulose (9)

[^{1}H NMR, 400 MHz, $CDCl_3$]

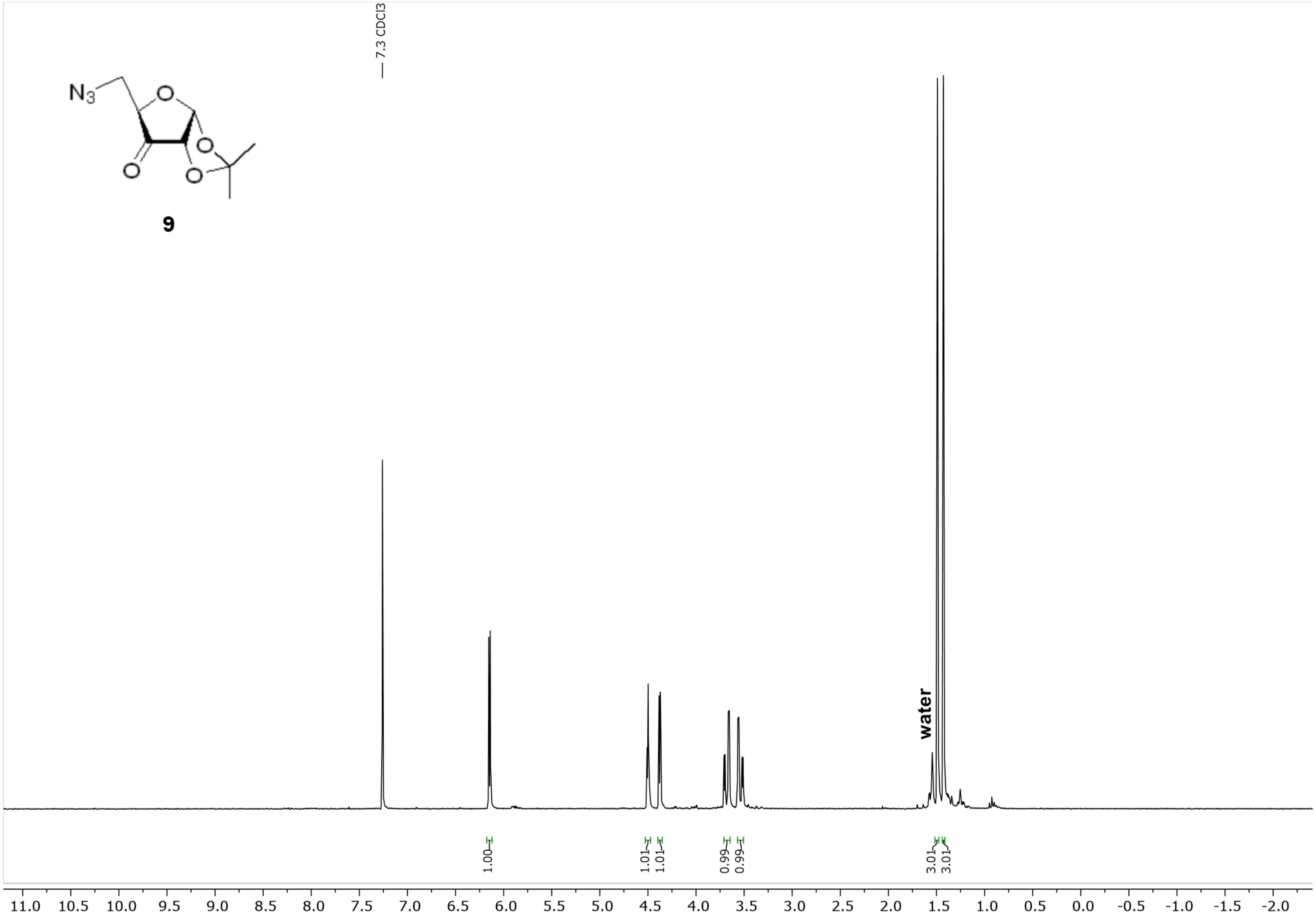

2-(5-Bromopentyloxy)-tetrahydro-2H-pyran (10)

[^{1}H NMR, 400 MHz, $CDCl_3$]

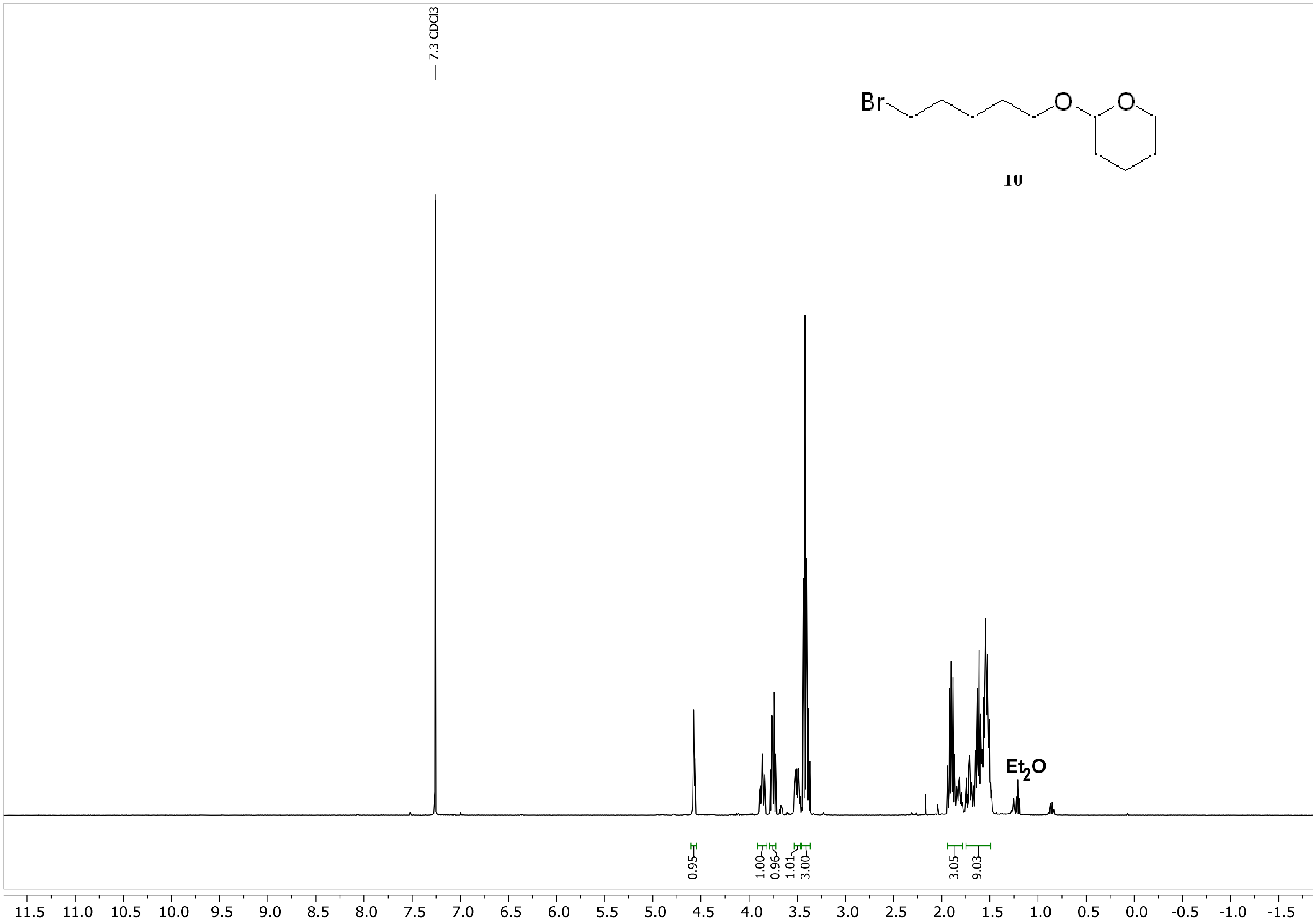

5-Azido-5-deoxy-1,2-*O*-isopropylidene-3-*C*-(6-(5-((tetrahydro-2H-pyran-2-yl)oxy)pentyl)-*α*-D-ribofuranose (12)

[^{1}H NMR, 400 MHz, $CDCl_3$]

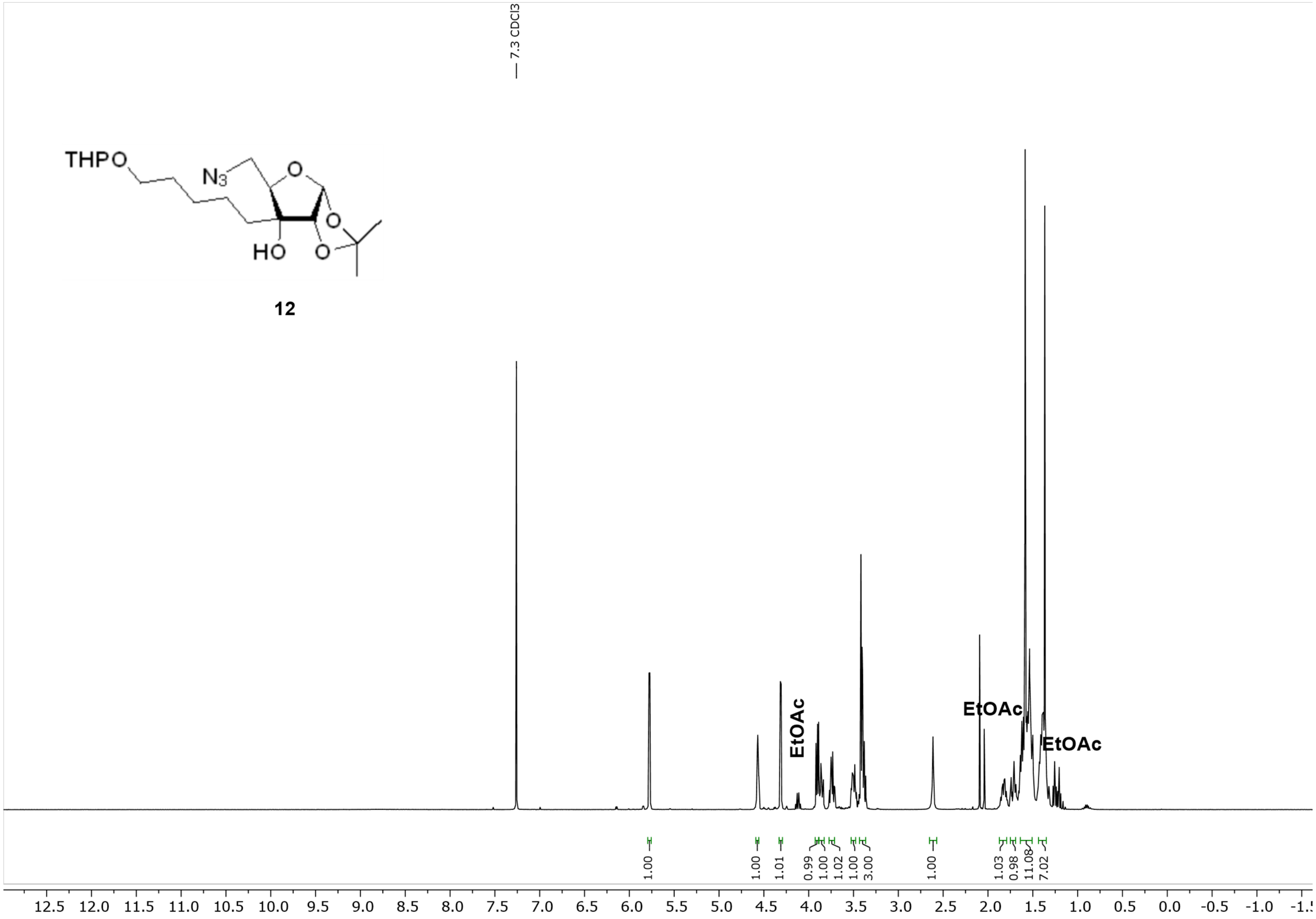

5-Azido-5-deoxy-1,2-*O*-isopropylidene-3-*C*-(6-(5-((tetrahydro-2H-pyran-2-yl)oxy)pentyl)-*α*-D-ribofuranose (12)

[^{13}C NMR, 101 MHz, $CDCl_3$]

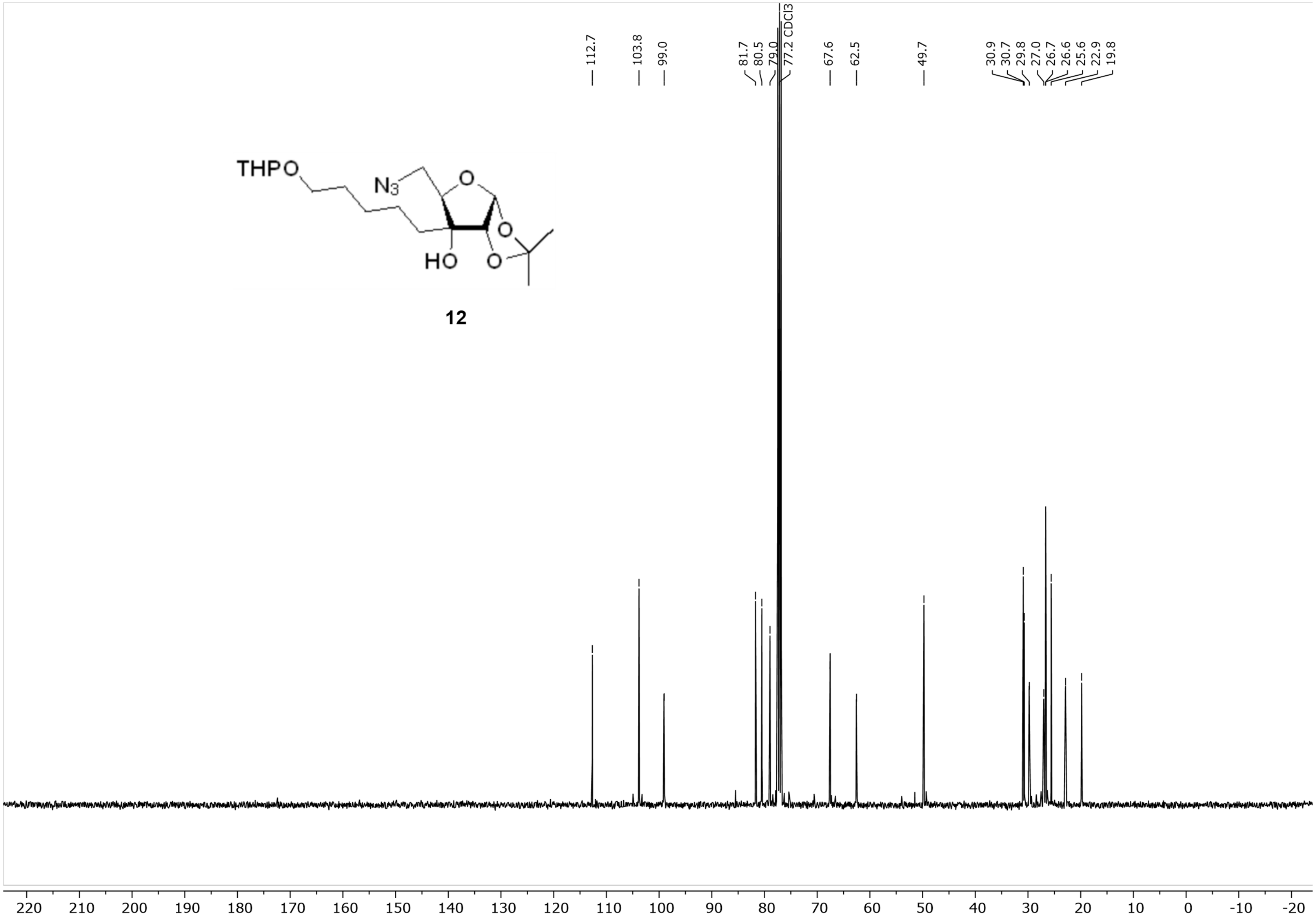

5-Azido-5-deoxy-1,2-*O*-isopropylidene-3-*C*-(6-(5-((tetrahydro-2H-pyran-2-yl)oxy)pentyl)-*α*-D-ribofuranose (12)

[HSQC, 400 MHz, $CDCl_3$]

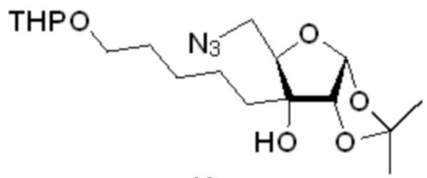

12

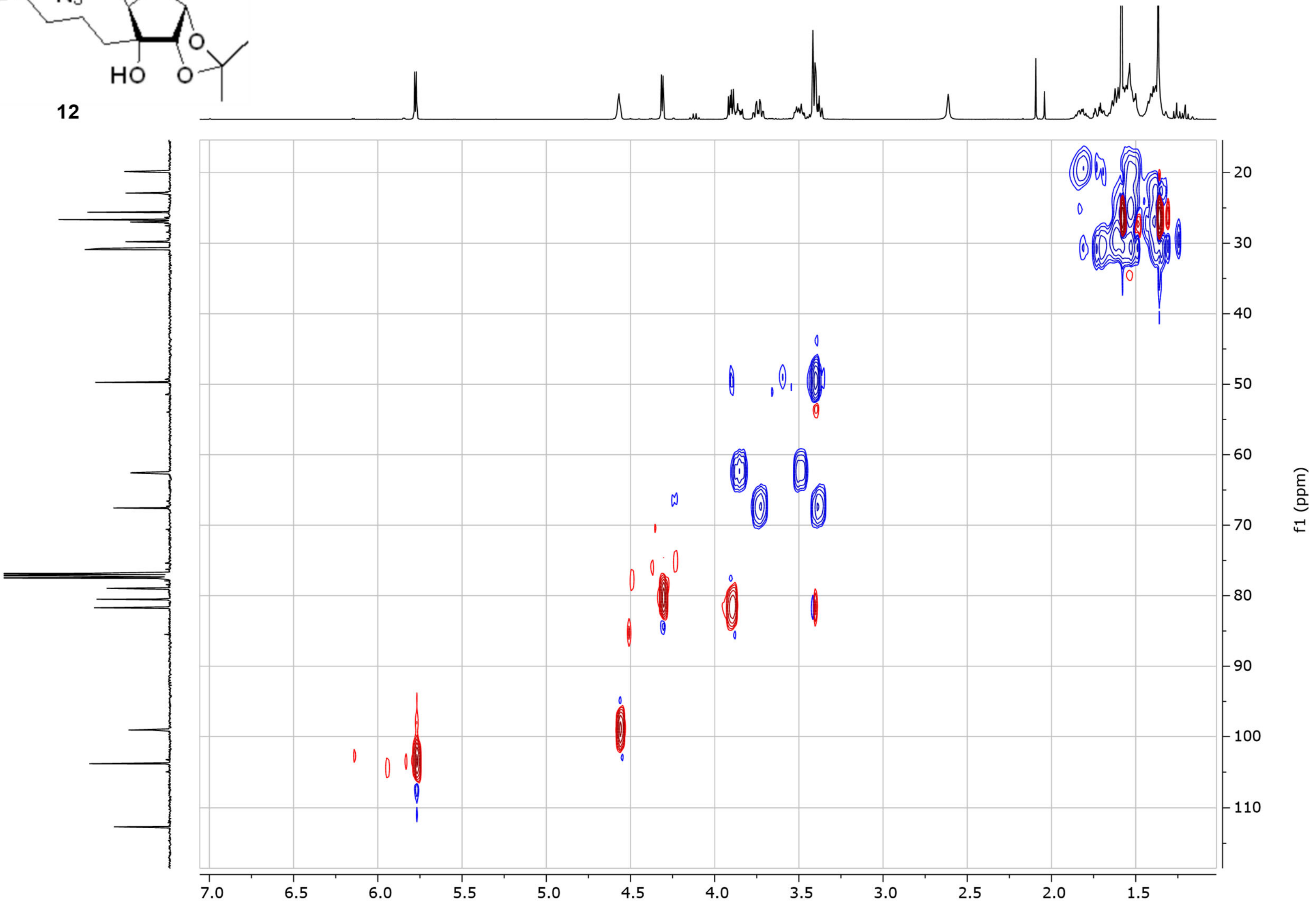

5-Azido-5-deoxy-1,2-*O*-isopropylidene-3-*C*-(6-(5-((tetrahydro-2H-pyran-2-yl)oxy)pentyl)-*α*-D-ribofuranose (12)

[HMBC, 400 MHz, $CDCl_3$]

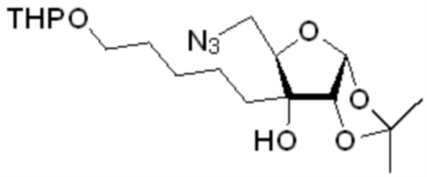

12

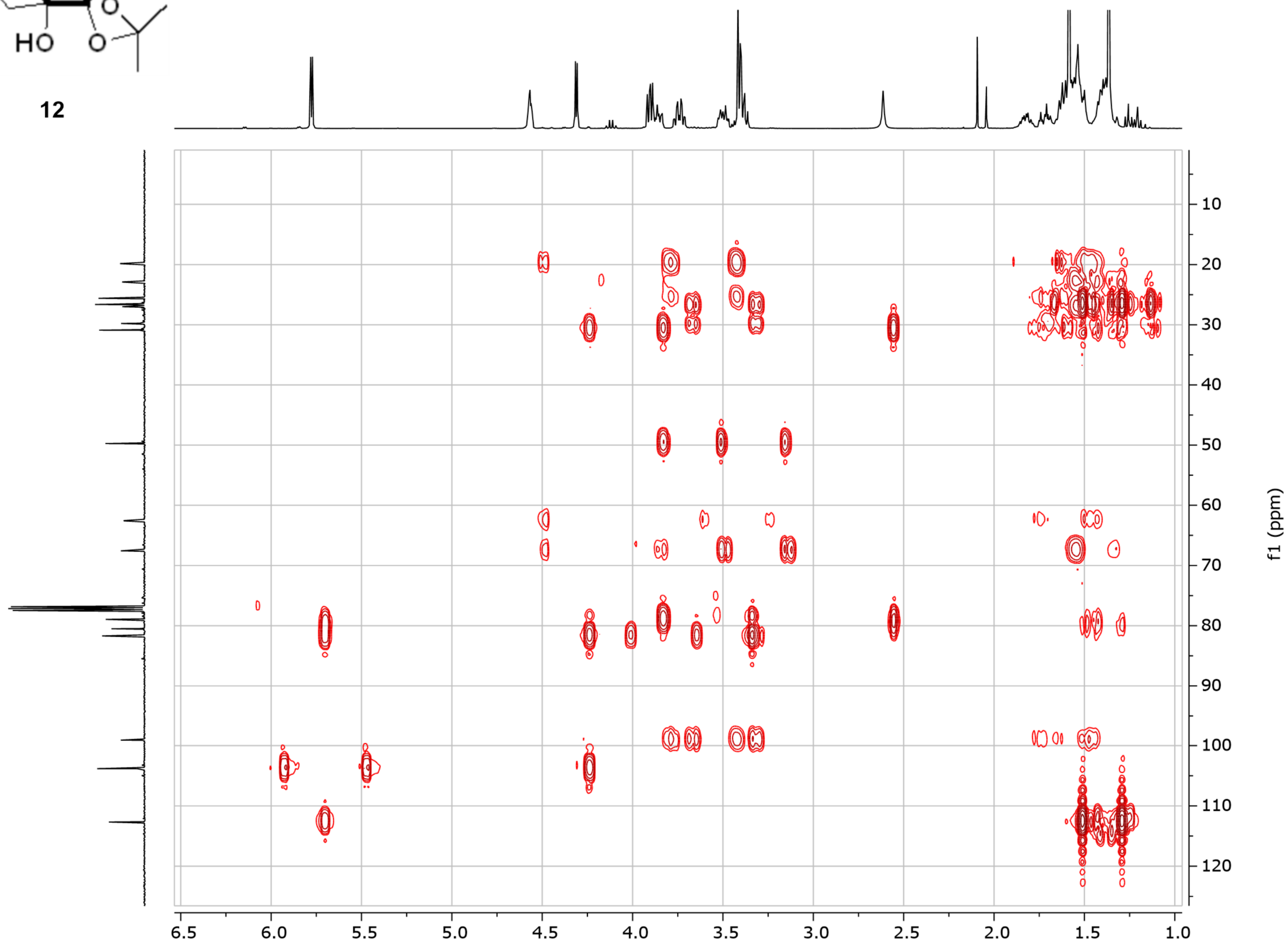

5-Azido-5-deoxy-1,2-*O*-isopropylidene-3-*C*-(6-(5-((tetrahydro-2H-pyran-2-yl)oxy)pentyl)-*α*-D-ribofuranose (12)

[COSY, 400 MHz, $CDCl_3$]

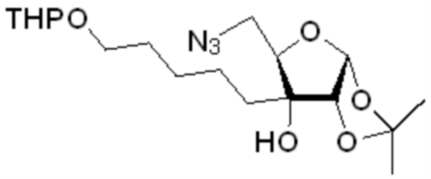

12

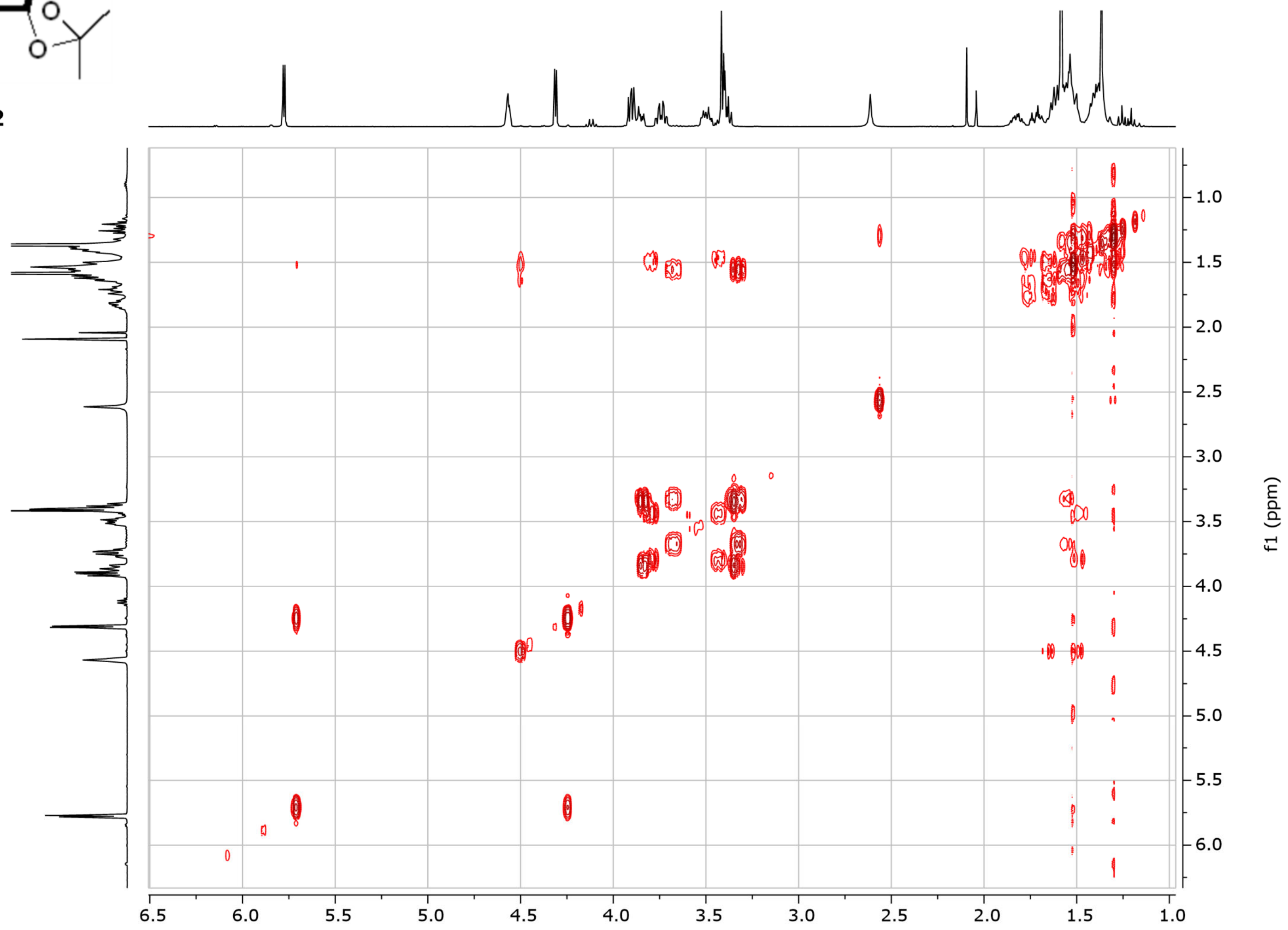

5-Azido-5-deoxy-1,2-*O*-isopropylidene-3-*C*-(6-(5-((tetrahydro-2H-pyran-2-yl)oxy)pentyl)-3-*O*-benzoyl-*α*-D-ribofuranose (13)

[^{1}H NMR, 400 MHz, $CDCl_3$]

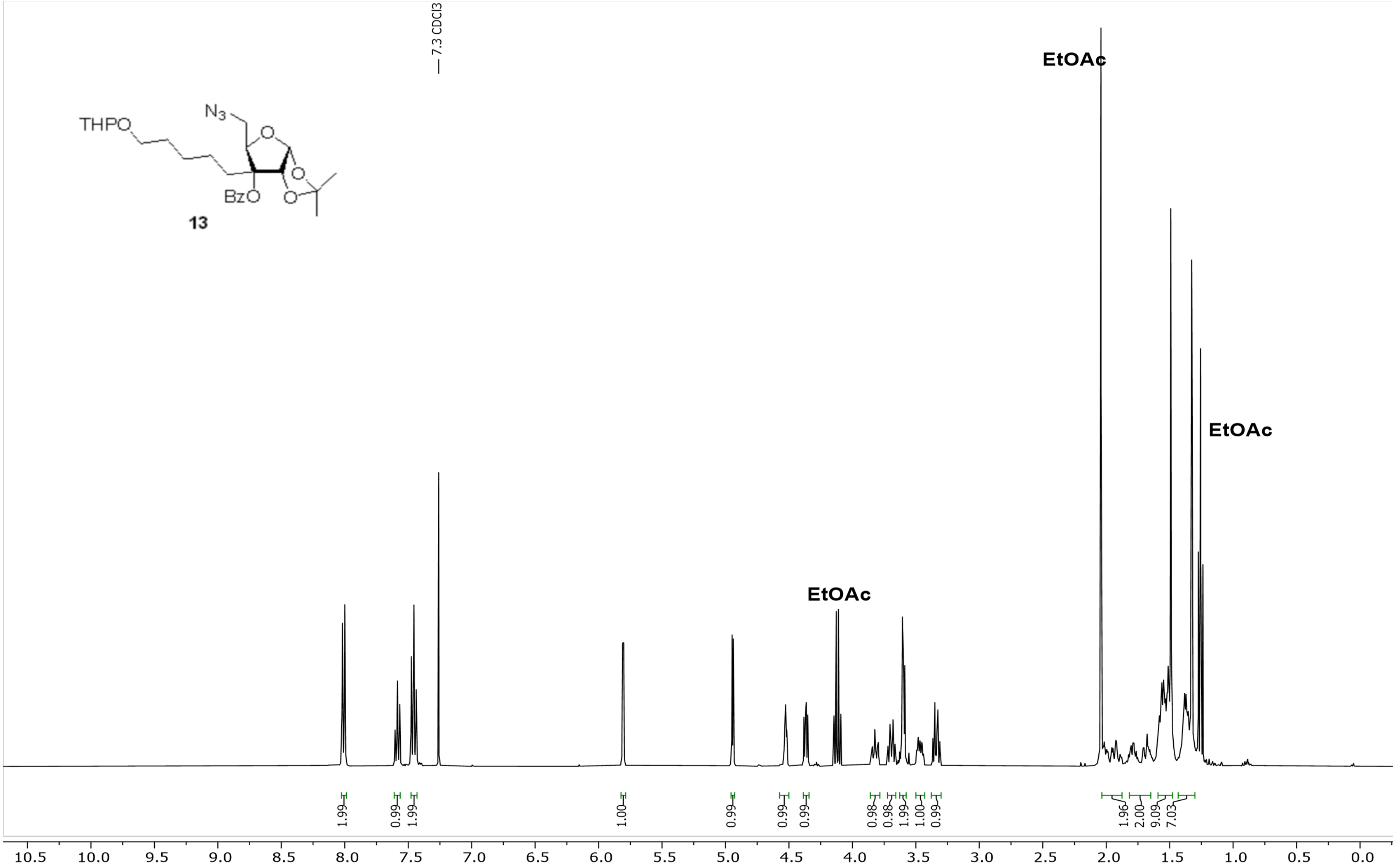

5-Azido-5-deoxy-1,2-*O*-isopropylidene-3-*C*-(6-(5-((tetrahydro-2H-pyran-2-yl)oxy)pentyl)-3-*O*-benzoyl-*α*-D-ribofuranose (13)

[^{13}C NMR, 101 MHz, $CDCl_3$]

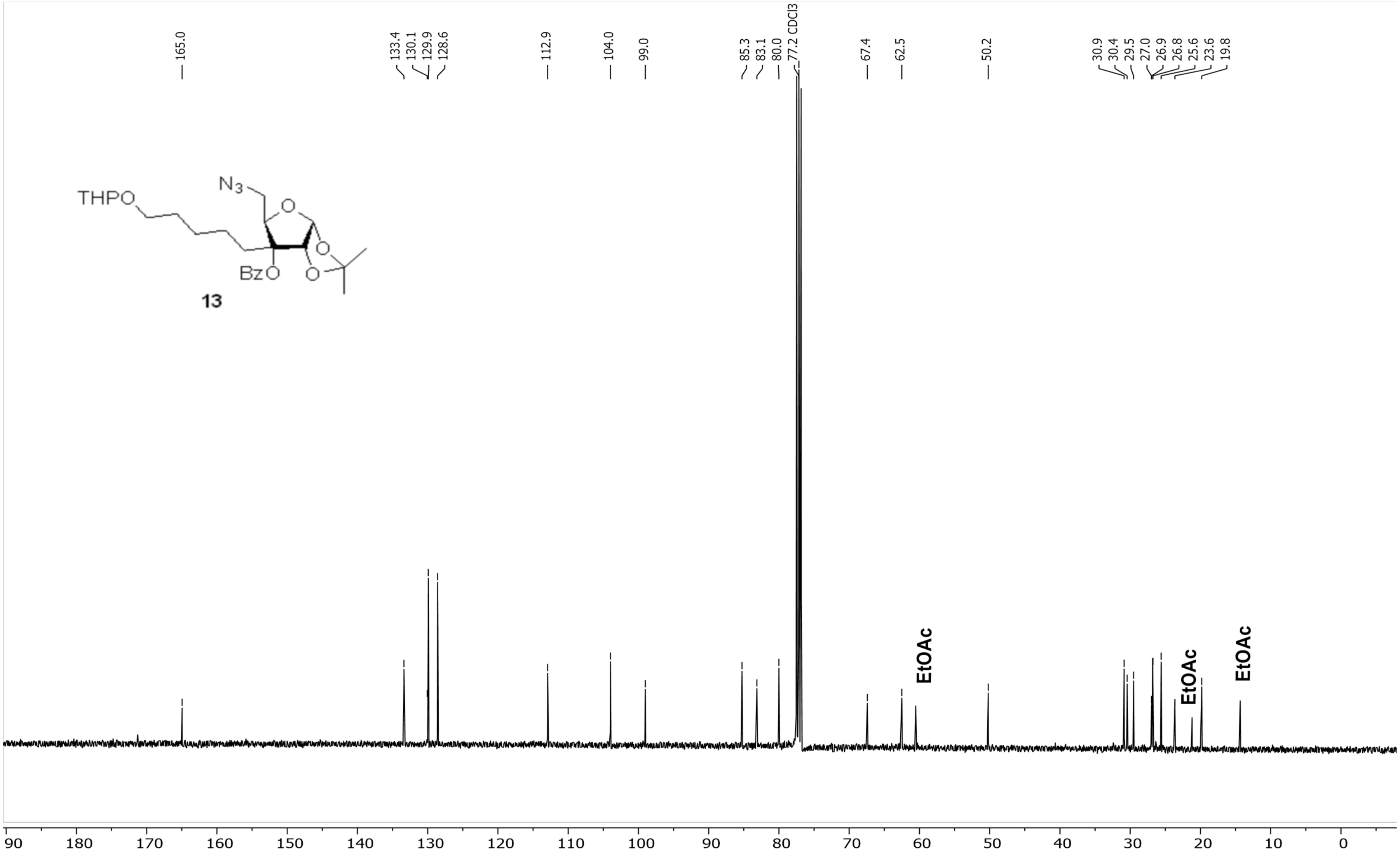

5-Azido-5-deoxy-1,2-*O*-isopropylidene-3-*C*-(6-(5-((tetrahydro-2H-pyran-2-yl)oxy)pentyl)-3-*O*-benzoyl-*α*-D-ribofuranose (13)
[HSQC, 400 MHz, $CDCl_3$]

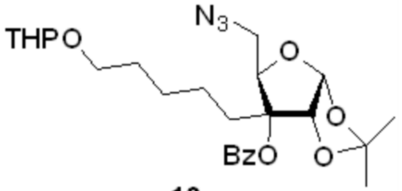

13

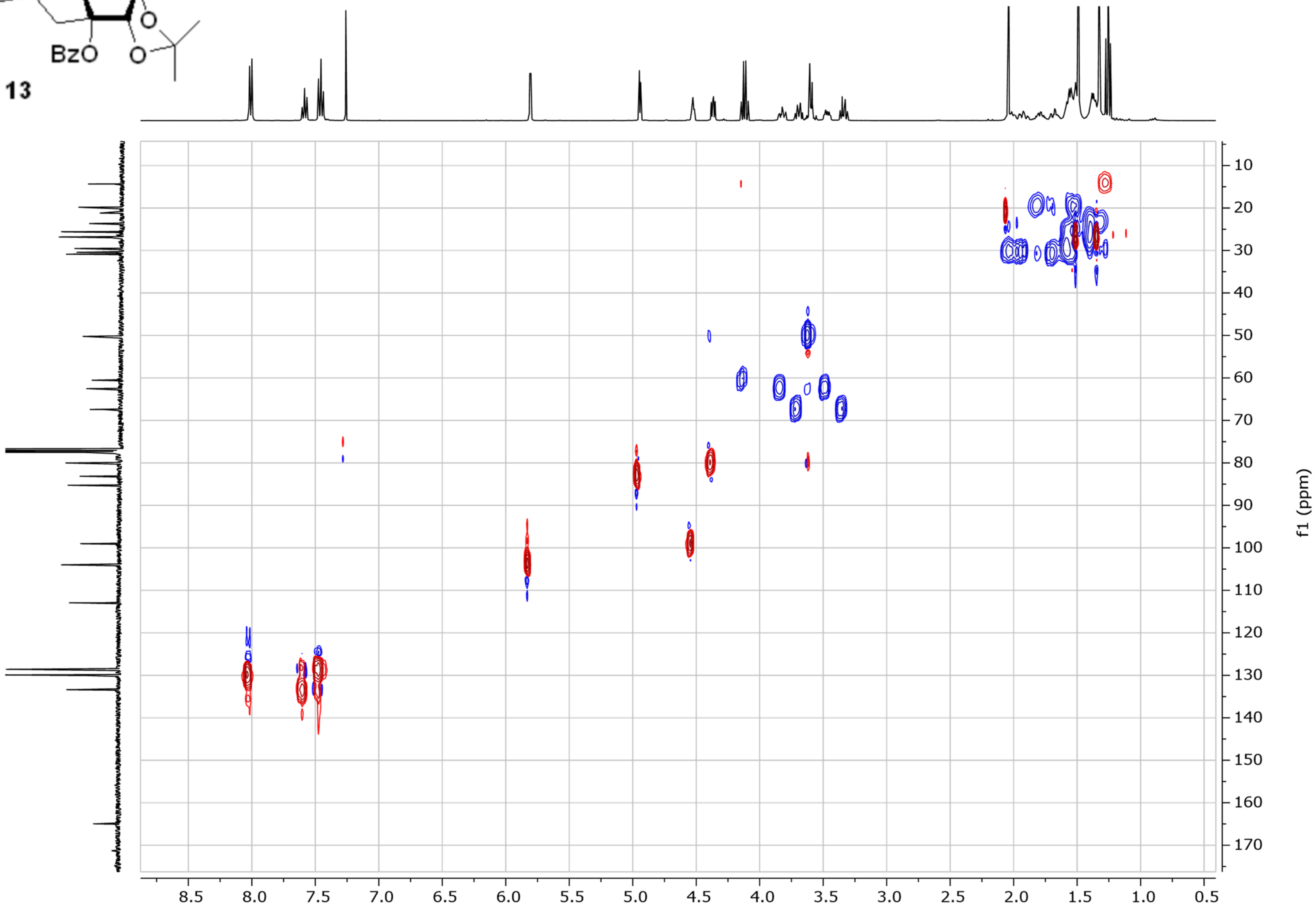

5-Azido-5-deoxy-1,2-*O*-isopropylidene-3-*C*-(6-(5-((tetrahydro-2H-pyran-2-yl)oxy)pentyl)-3-*O*-benzoyl-*α*-D-ribofuranose (13)
[HMBC, 400 MHz, $CDCl_3$]

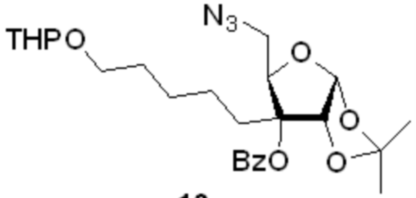

13

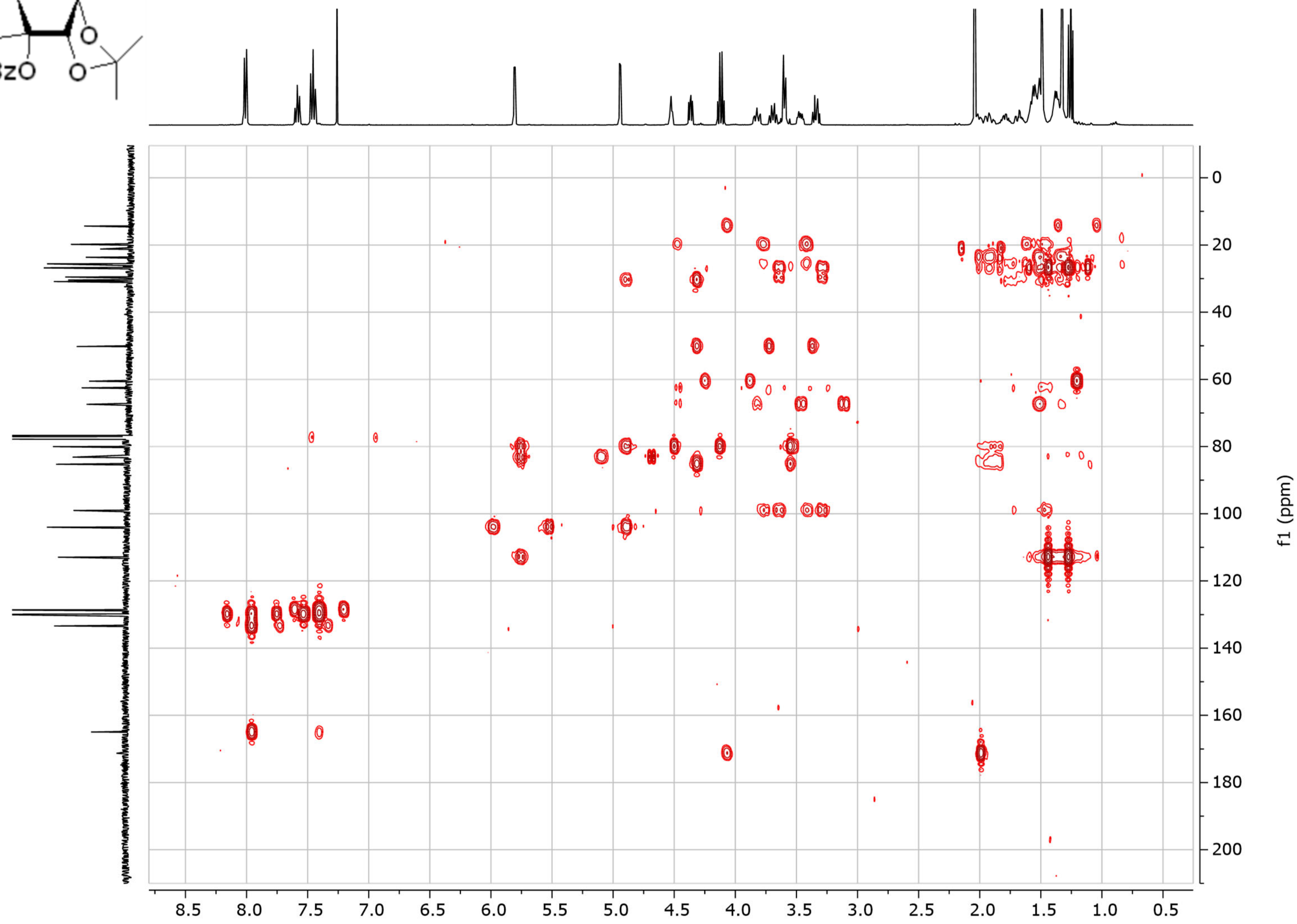

5-Azido-5-deoxy-1,2-*O*-isopropylidene-3-*C*-(6-(5-((tetrahydro-2H-pyran-2-yl)oxy)pentyl)-3-*O*-benzoyl-*α*-D-ribofuranose (13)

[COSY, 400 MHz, $CDCl_3$]

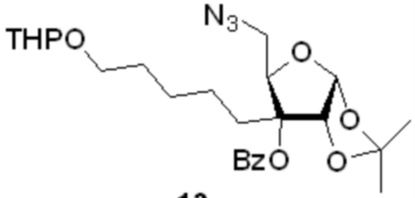

13

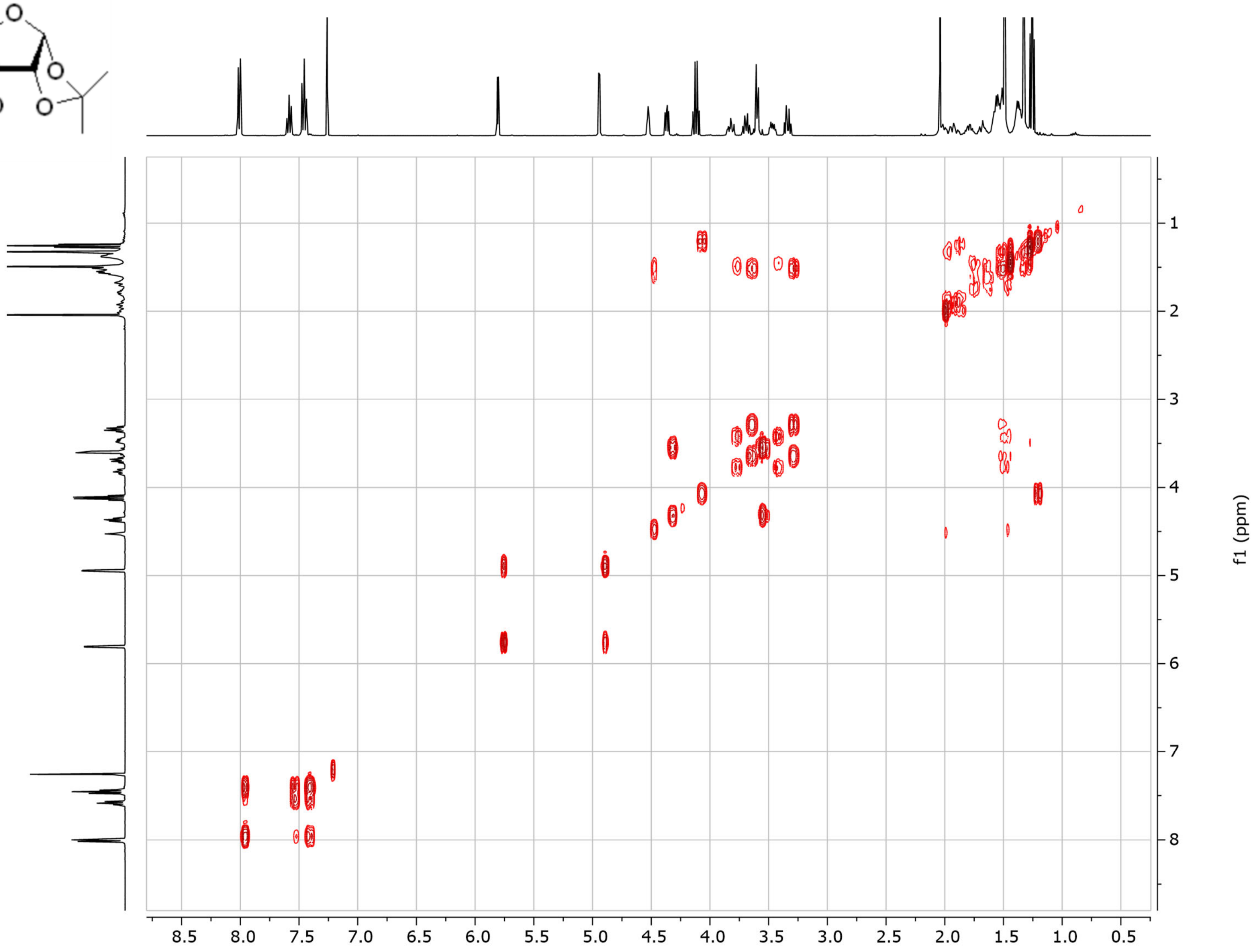

5-Azido-5-deoxy-1,2-*O*-isopropylidene-3-*C*-(5-hydroxypentyl)-3-*O*-benzoyl-*α*-D-ribofuranose (14)

[^{1}H NMR, 400 MHz, $CDCl_3$]

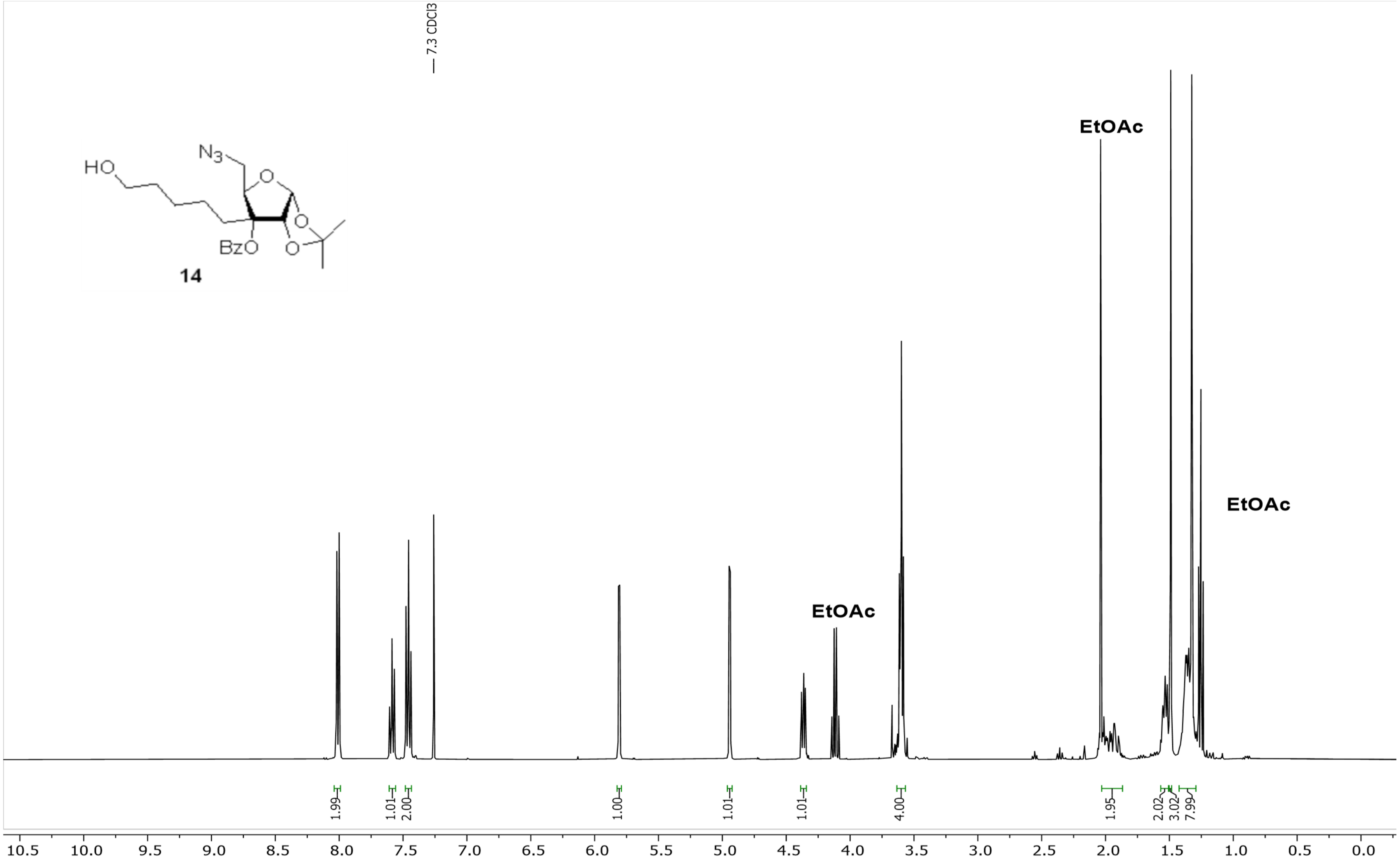

5-Azido-5-deoxy-1,2-*O*-isopropylidene-3-*C*-(5-hydroxypentyl)-3-*O*-benzoyl-*α*-D-ribofuranose (14)

[^{13}C NMR, 101 MHz, $CDCl_3$]

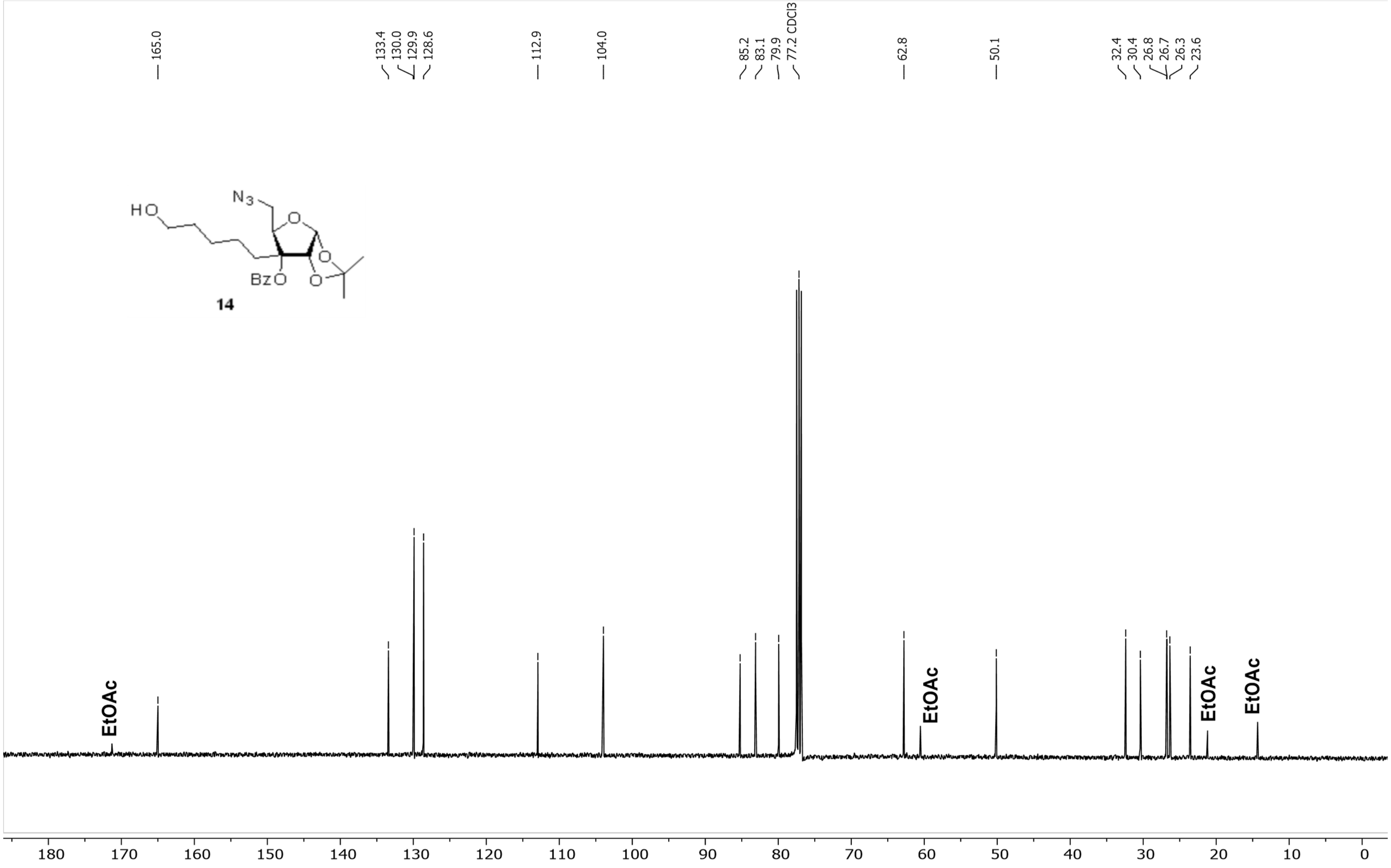

Azido-5-deoxy-1,2-*O*-isopropylidene-3-*C*-(5-hydroxypentyl)-3-*O*-benzoyl-*α*-D-ribofuranose (14)

[HSQC, 400 MHz, $CDCl_3$]

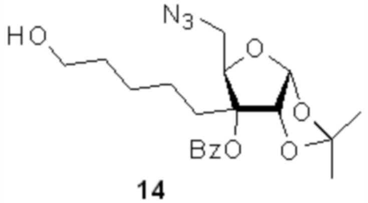

14

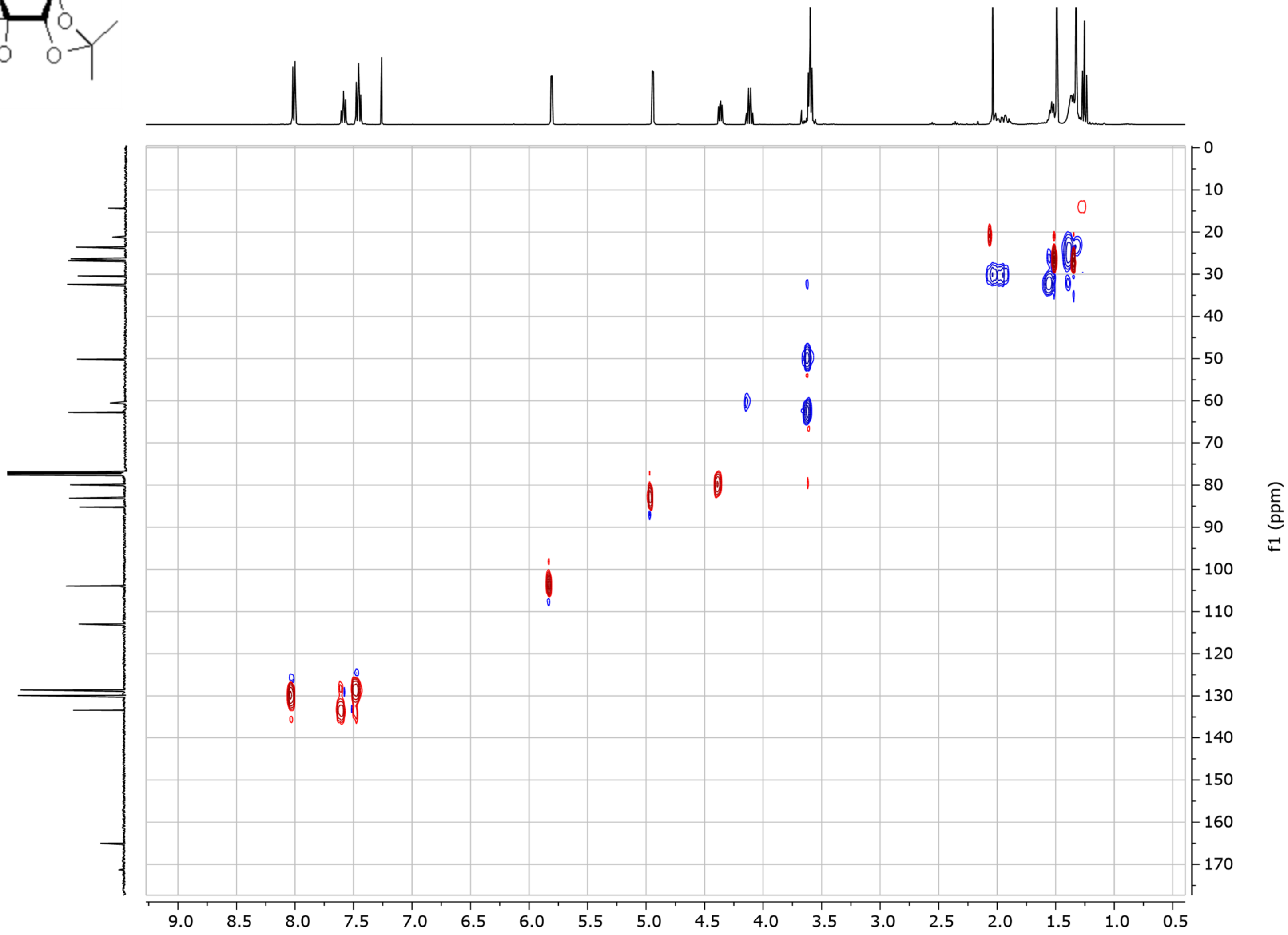

5-Azido-5-deoxy-1,2-*O*-isopropylidene-3-*C*-(5-hydroxypentyl)-3-*O*-benzoyl-*α*-D-ribofuranose (14)

[HMBC, 400 MHz, $CDCl_3$]

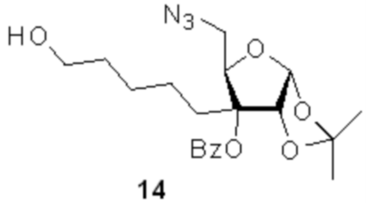

14

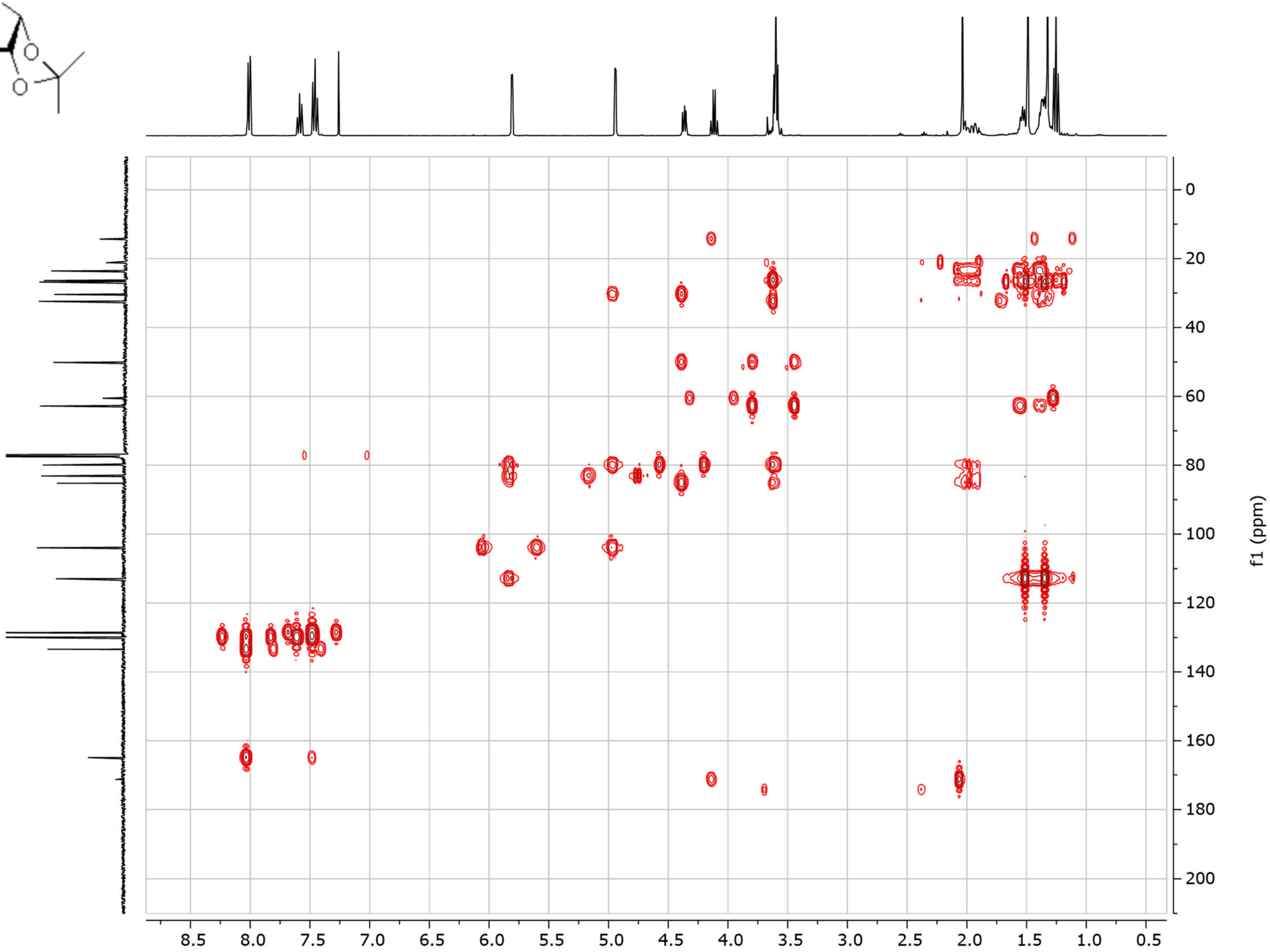

5-Azido-5-deoxy-1,2-*O*-isopropylidene-3-*C*-(5-hydroxypentyl)-3-*O*-benzoyl-*α*-D-ribofuranose (14)
[COSY, 400 MHz, $CDCl_3$]

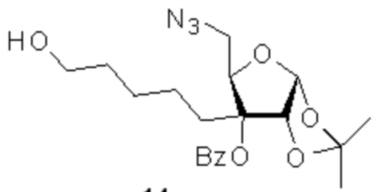

14

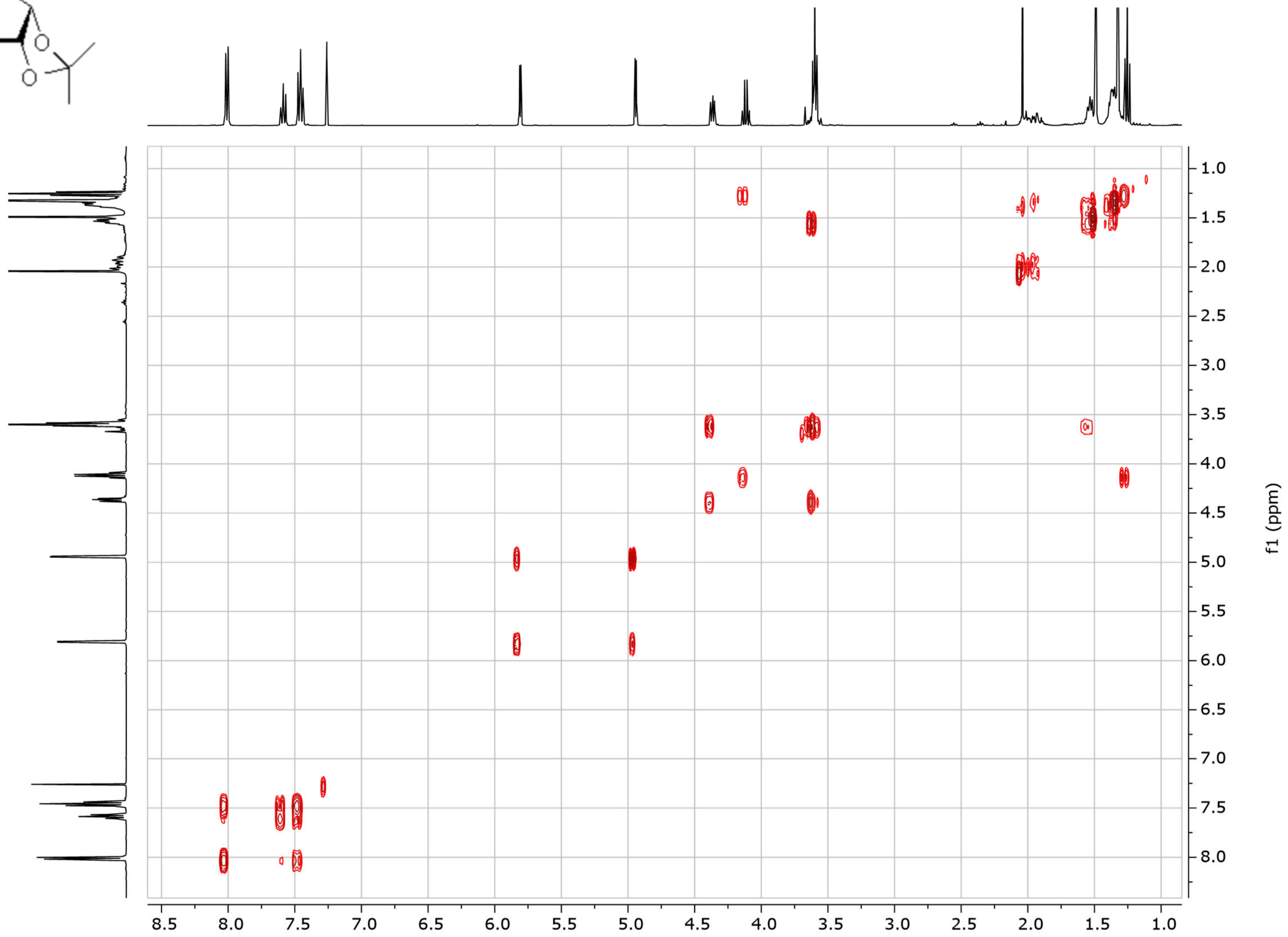

5-Azido-5-deoxy-1,2-*O*-isopropylidene-3-*C*-(5-oxopentyl)-3-*O*-benzoyl-*α*-D-ribofuranose (15)

[^{1}H NMR, 400 MHz, $CDCl_3$]

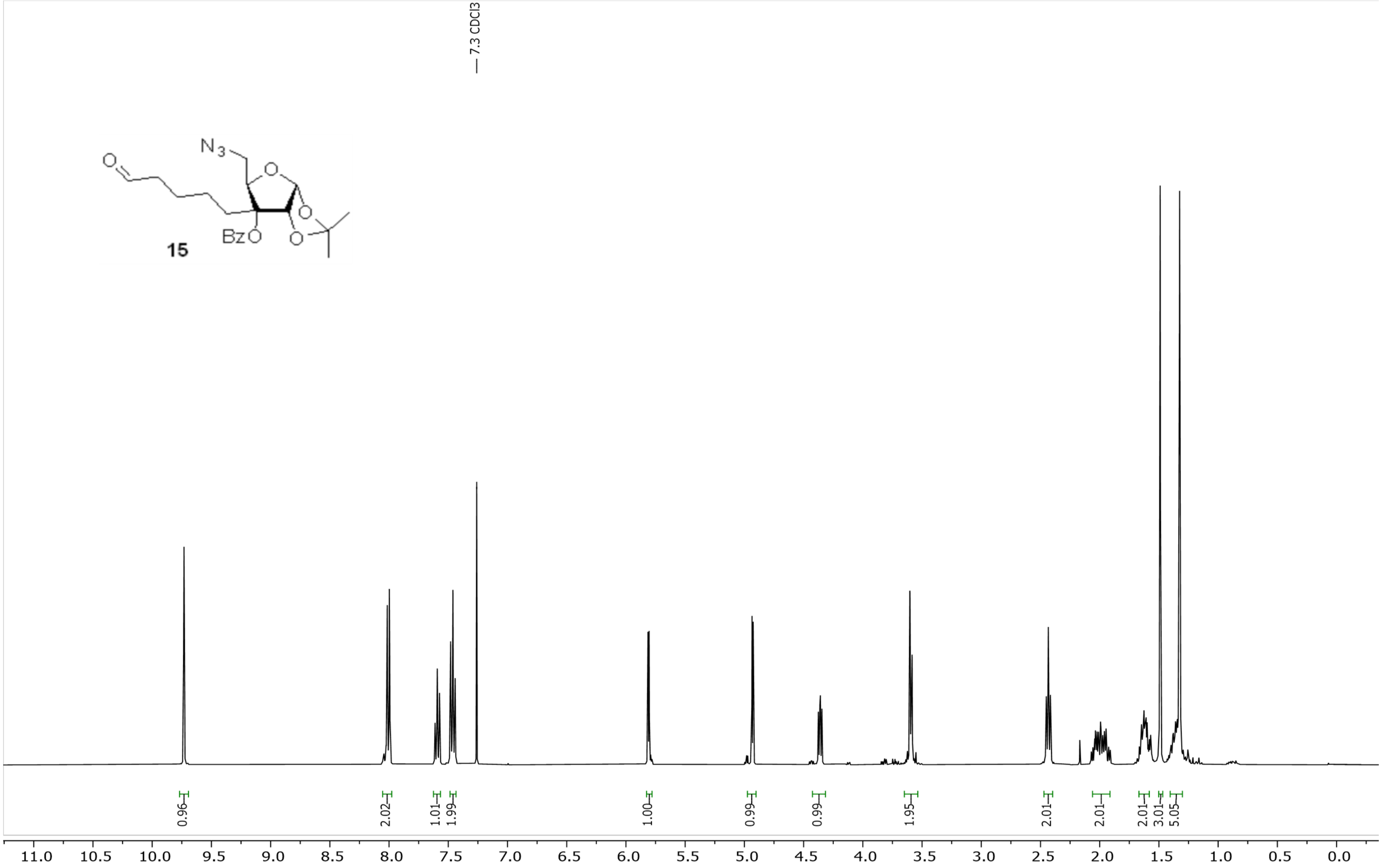

5-Azido-5-deoxy-1,2-*O*-isopropylidene-3-*C*-(5-oxopentyl)-3-*O*-benzoyl-*α*-D-ribofuranose (15)

[^{13}C NMR, 101 MHz, $CDCl_3$]

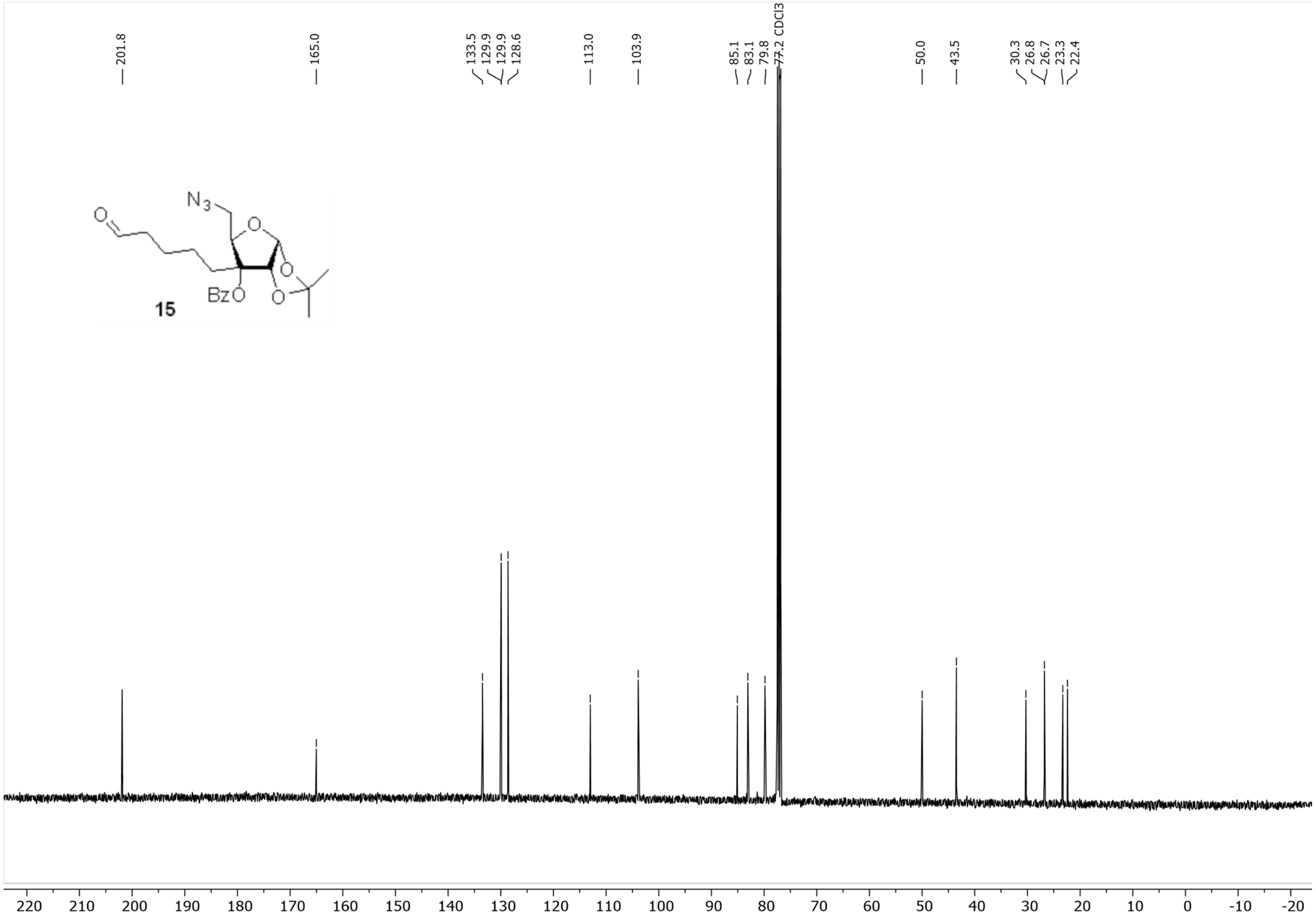

5-Azido-5-deoxy-1,2-*O*-isopropylidene-3-*C*-(5-oxopentyl)-3-*O*-benzoyl-*α*-D-ribofuranose (15)

[HSQC, 400 MHz, $CDCl_3$]

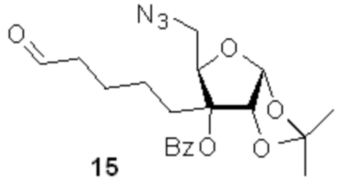

15

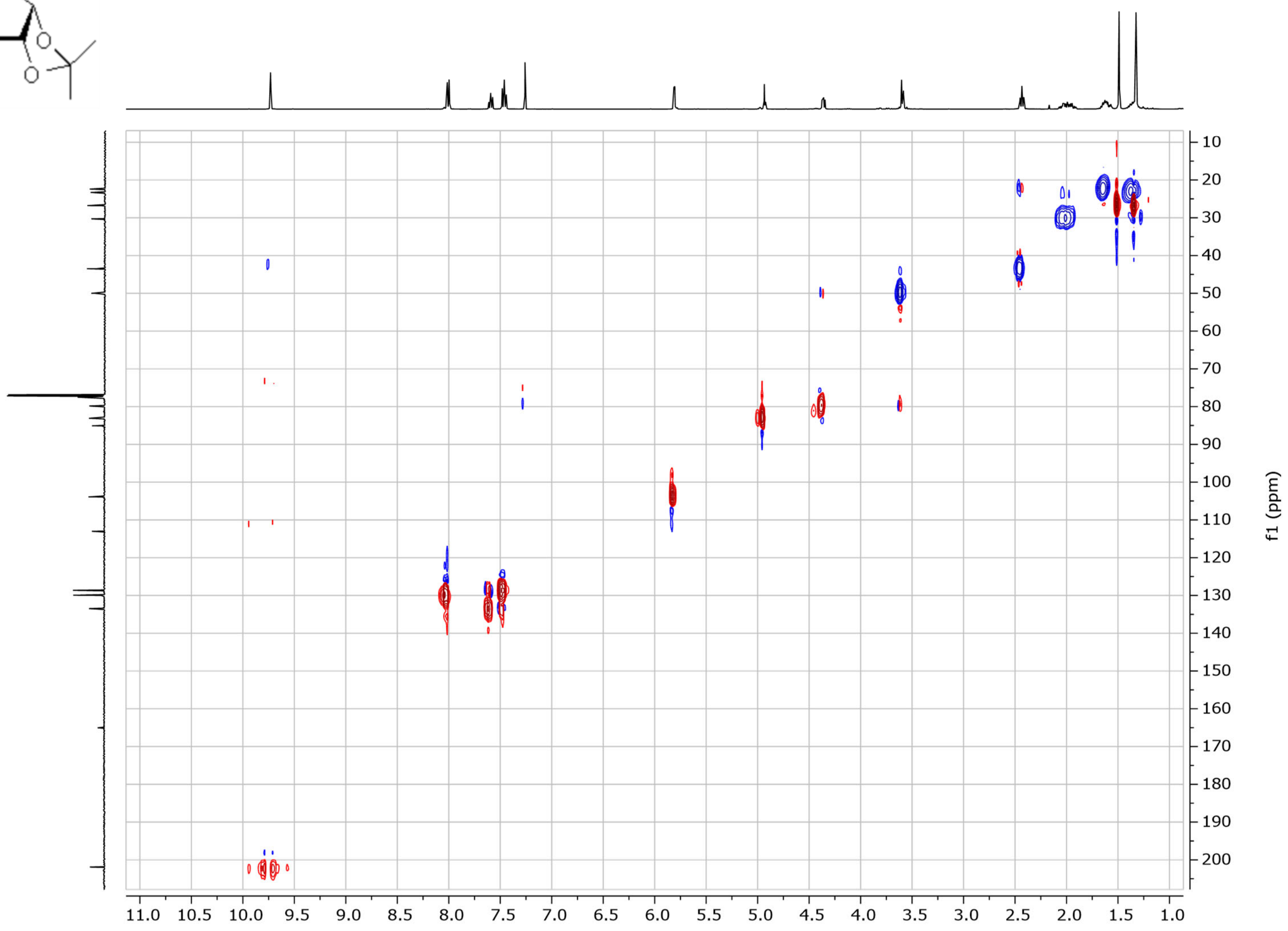

5-Azido-5-deoxy-1,2-*O*-isopropylidene-3-*C*-(5-oxopentyl)-3-*O*-benzoyl-*α*-D-ribofuranose (15)

[HMBC, 400 MHz, $CDCl_3$]

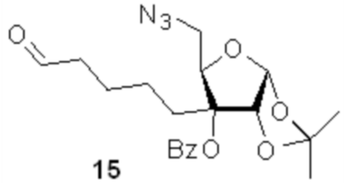

15

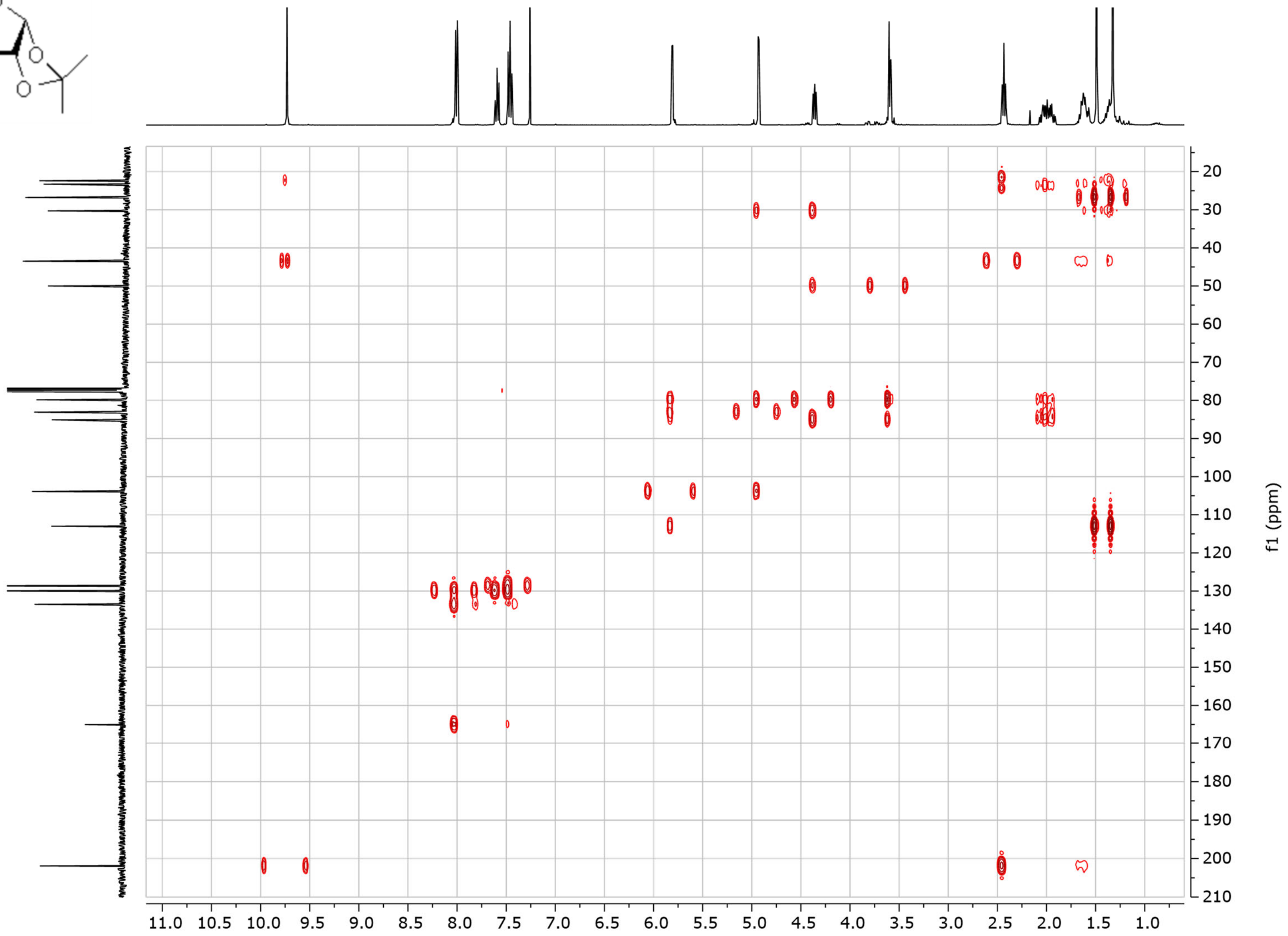

5-Azido-5-deoxy-1,2-*O*-isopropylidene-3-*C*-(5-oxopentyl)-3-*O*-benzoyl-*α*-D-ribofuranose (15)

[COSY, 400 MHz, $CDCl_3$]

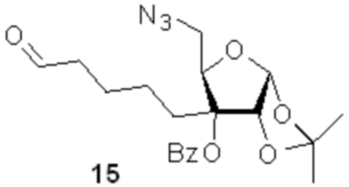

15

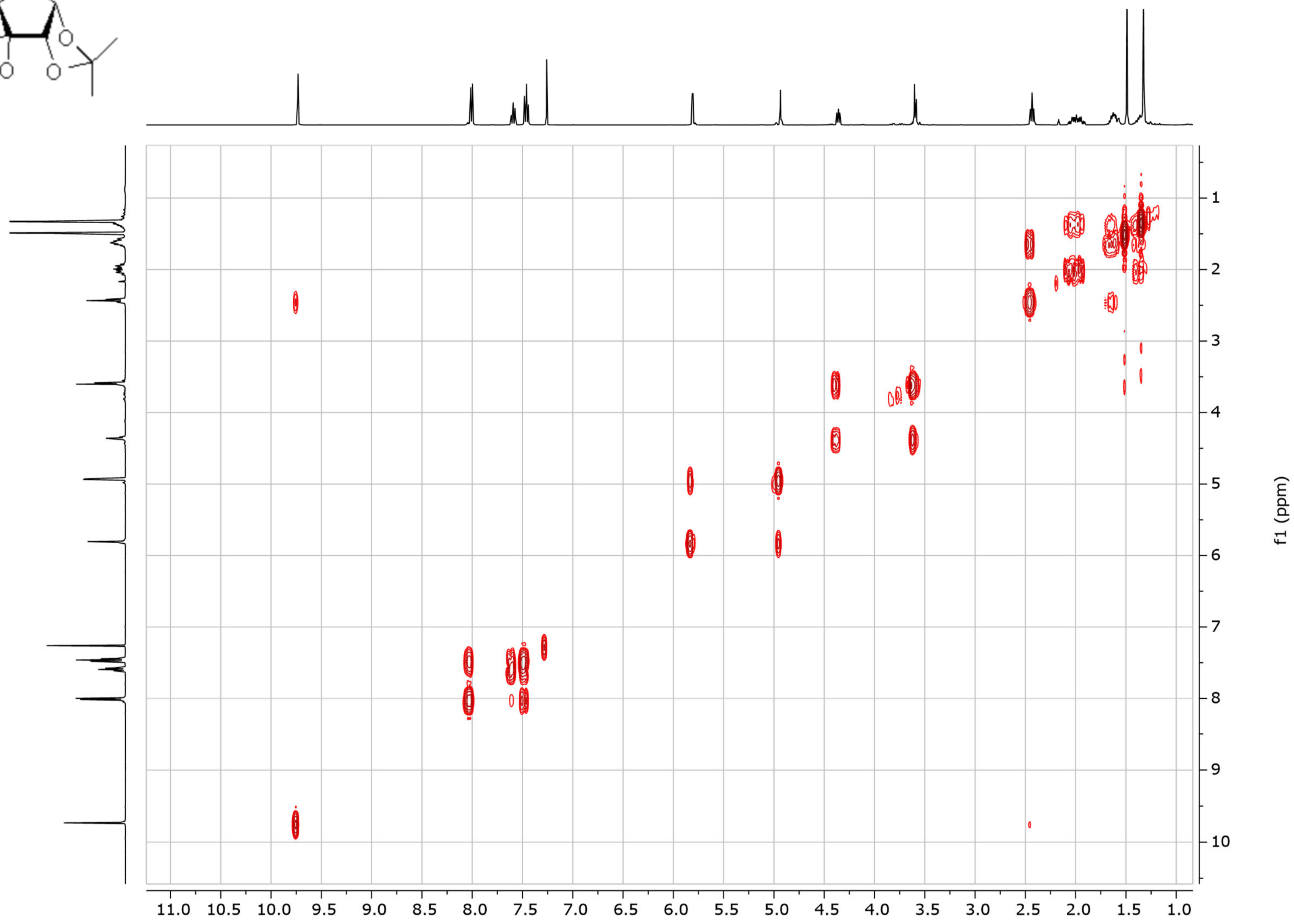

5-Azido-5-deoxy-1,2-*O*-isopropylidene-3-*C*-(5-(dimethylamino)pentyl)-3-*O*-benzoyl-*α*-D-ribofuranose (16)
[^{1}H NMR, 400 MHz, $CDCl_3$]

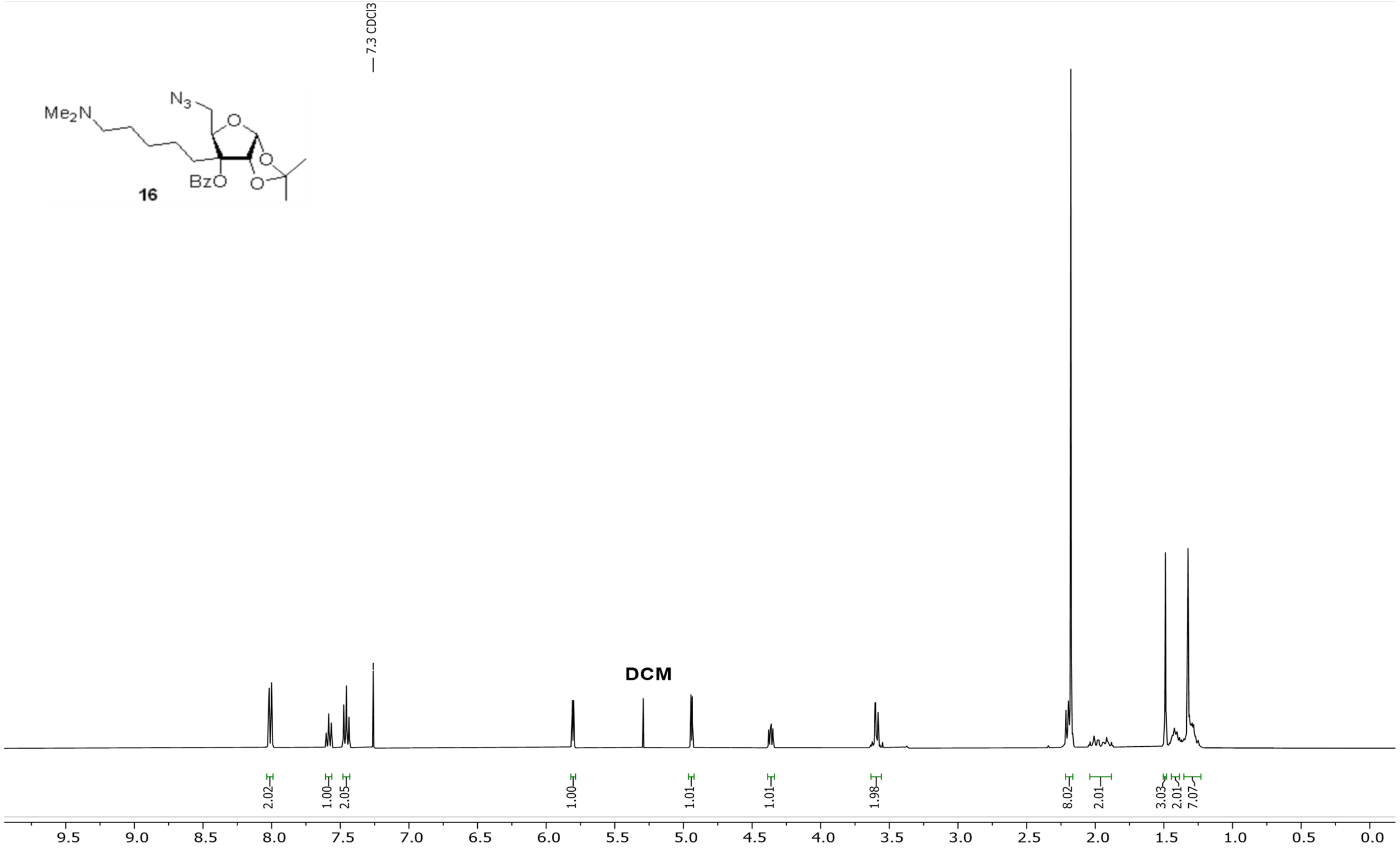

5-Azido-5-deoxy-1,2-*O*-isopropylidene-3-*C*-(5-(dimethylamino)pentyl)-3-*O*-benzoyl-*α*-D-ribofuranose (16)
[^{13}C NMR, 101 MHz, $CDCl_3$]

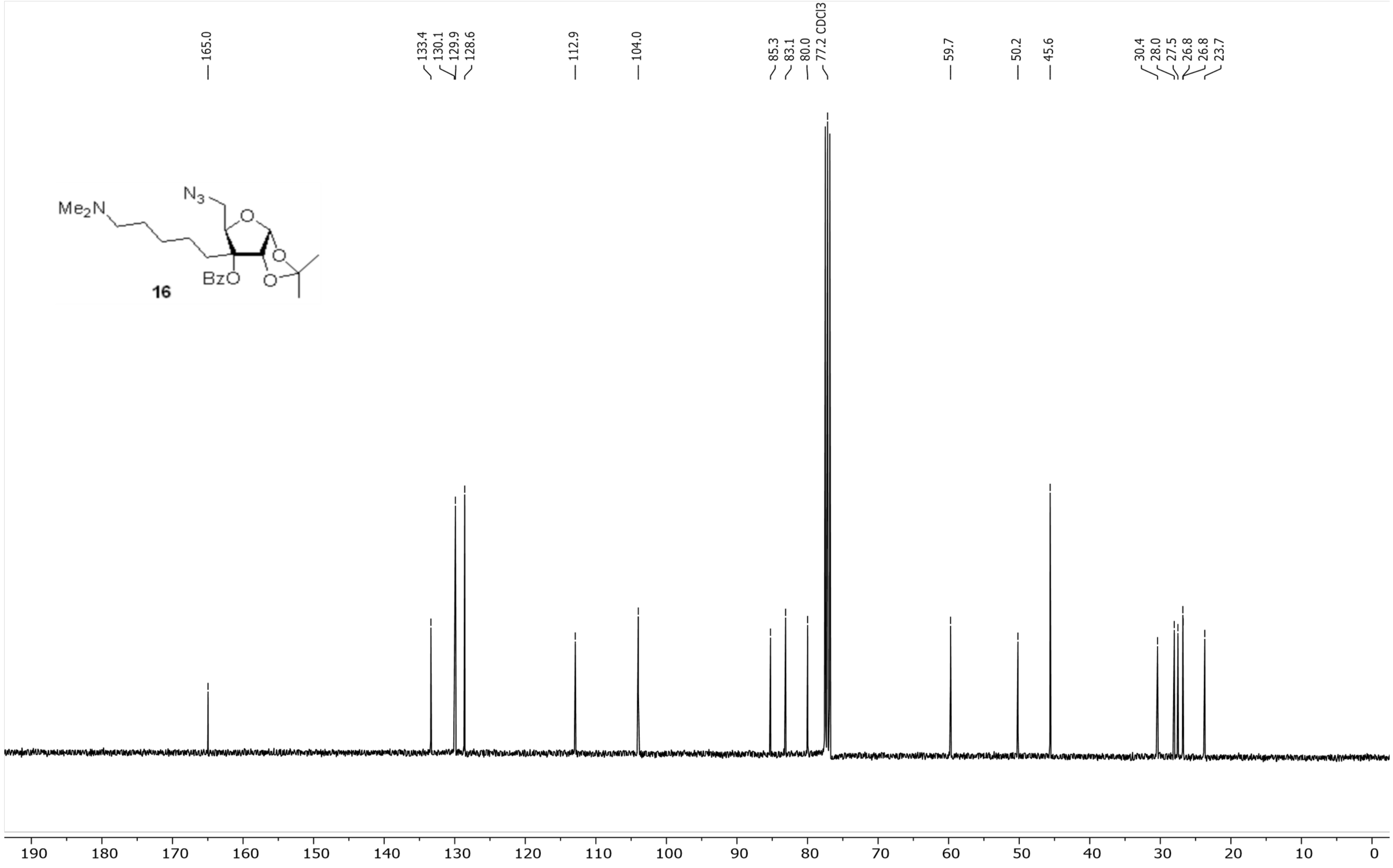

5-Azido-5-deoxy-1,2-*O*-isopropylidene-3-*C*-(5-(dimethylamino)pentyl)-3-*O*-benzoyl-*α*-D-ribofuranose (16)
[HSQC, 400 MHz, $CDCl_3$]

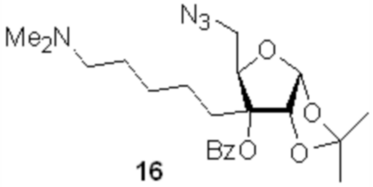

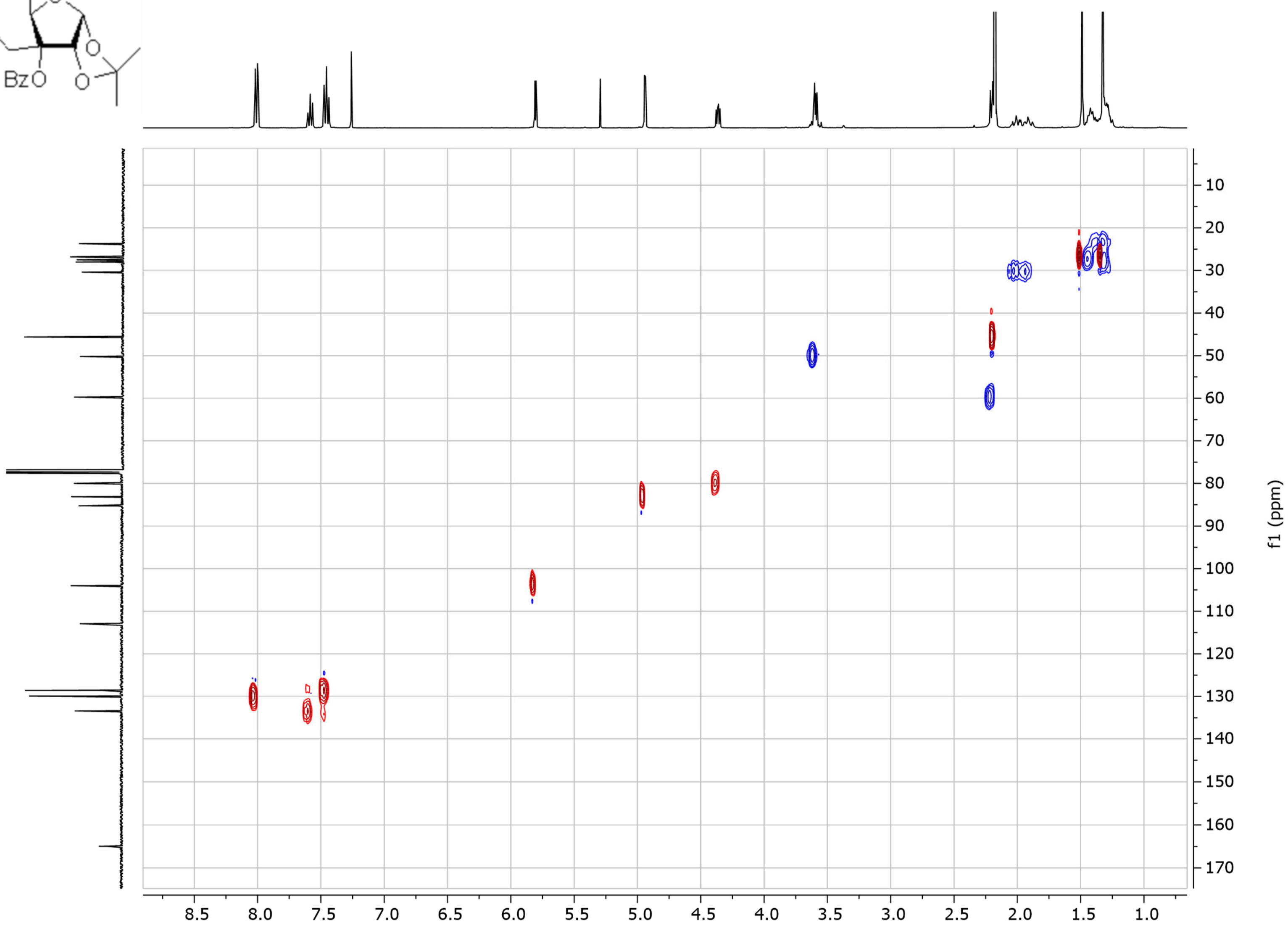

5-Azido-5-deoxy-1,2-*O*-isopropylidene-3-*C*-(5-(dimethylamino)pentyl)-3-*O*-benzoyl-*α*-D-ribofuranose (16)

[HMBC, 400 MHz, $CDCl_3$]

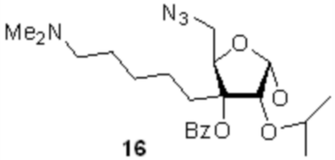

16

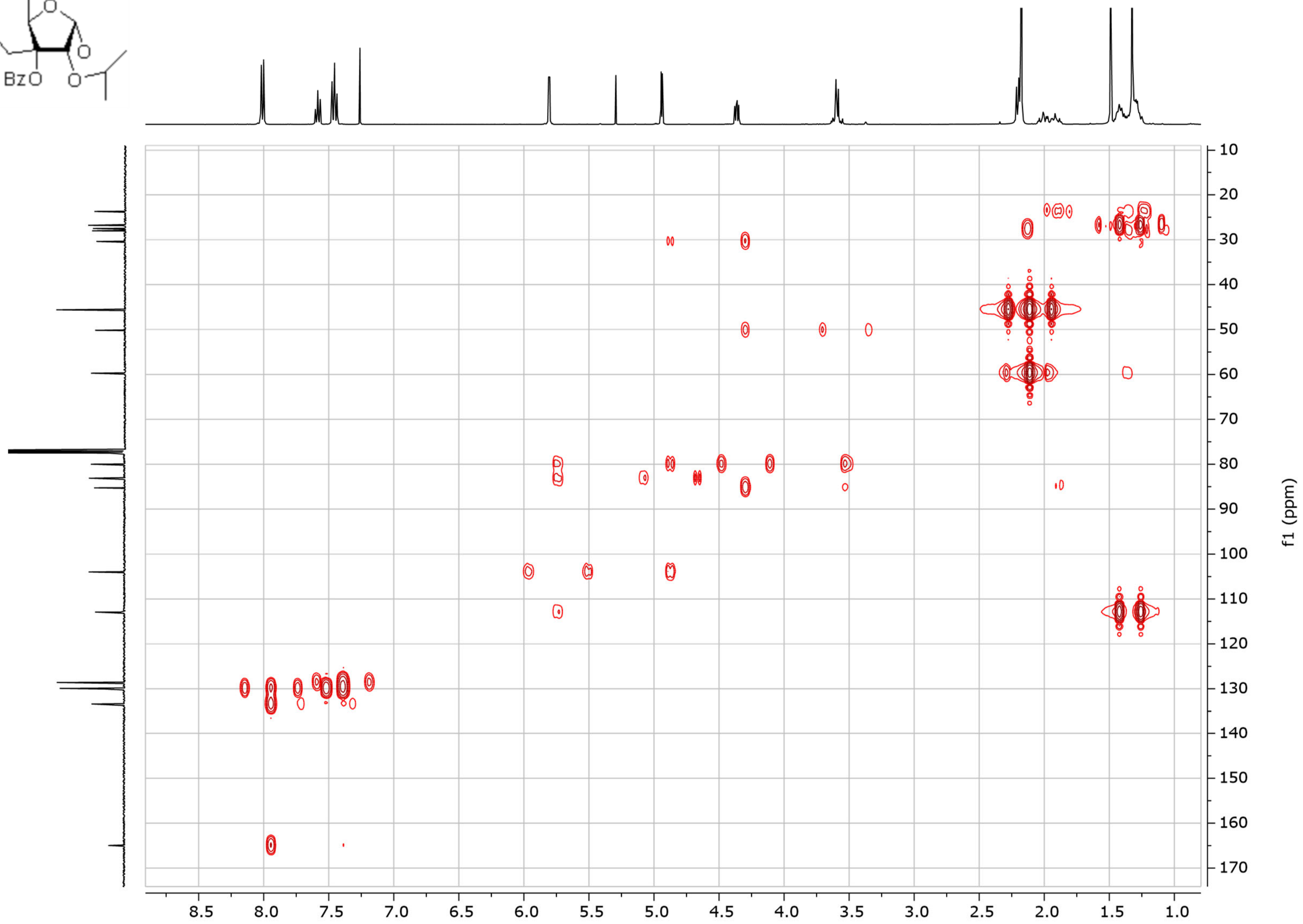

5-Azido-5-deoxy-1,2-*O*-isopropylidene-3-*C*-(5-(dimethylamino)pentyl)-3-*O*-benzoyl-*α*-D-ribofuranose (16)

[COSY, 400 MHz, $CDCl_3$]

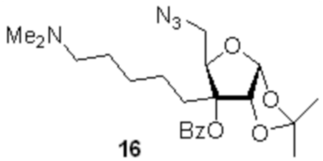

16

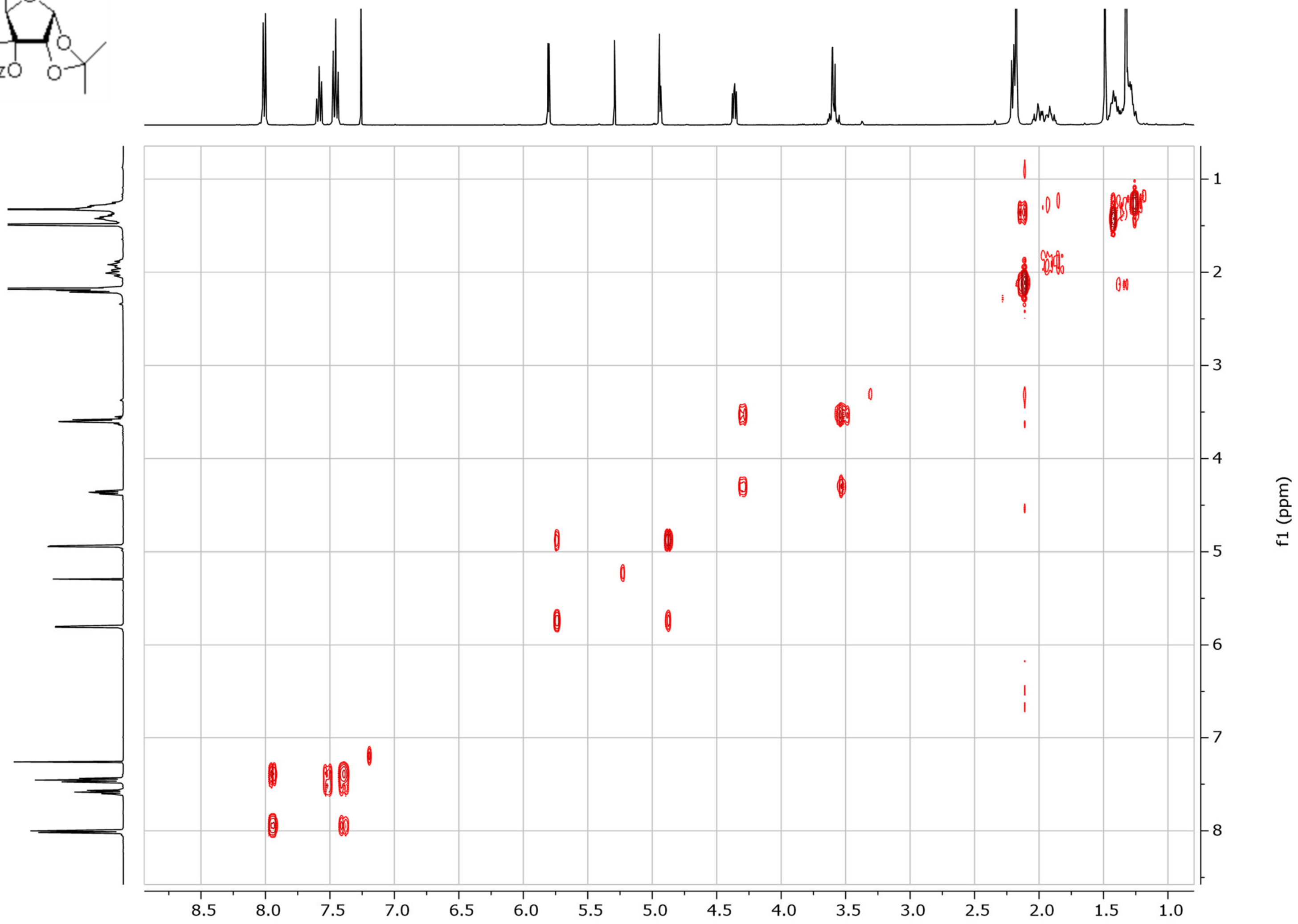

1,2-Di-*O*-acetyl-5-azido-5-deoxy-3-*C*-(5-(dimethylamino)pentyl)-3-*O*-benzoyl-*α/β*-D-ribofuranose (17)

[^{1}H NMR, 400 MHz, $CDCl_3$]

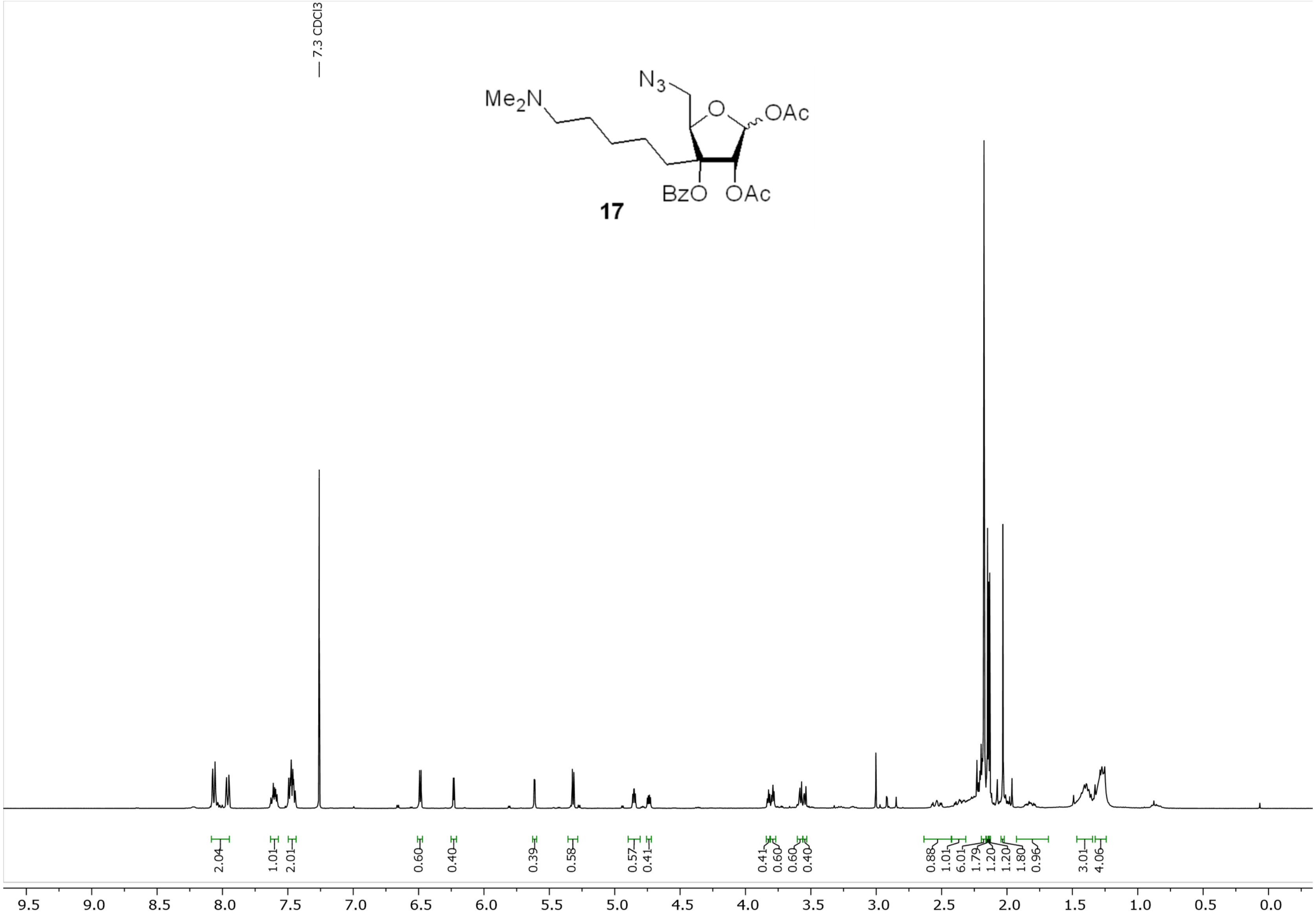

1,2-Di-*O*-acetyl-5-azido-5-deoxy-3-*C*-(5-(dimethylamino)pentyl)-3-*O*-benzoyl-*α/β*-D-ribofuranose (17)

[^{13}C NMR, 101 MHz, $CDCl_3$]

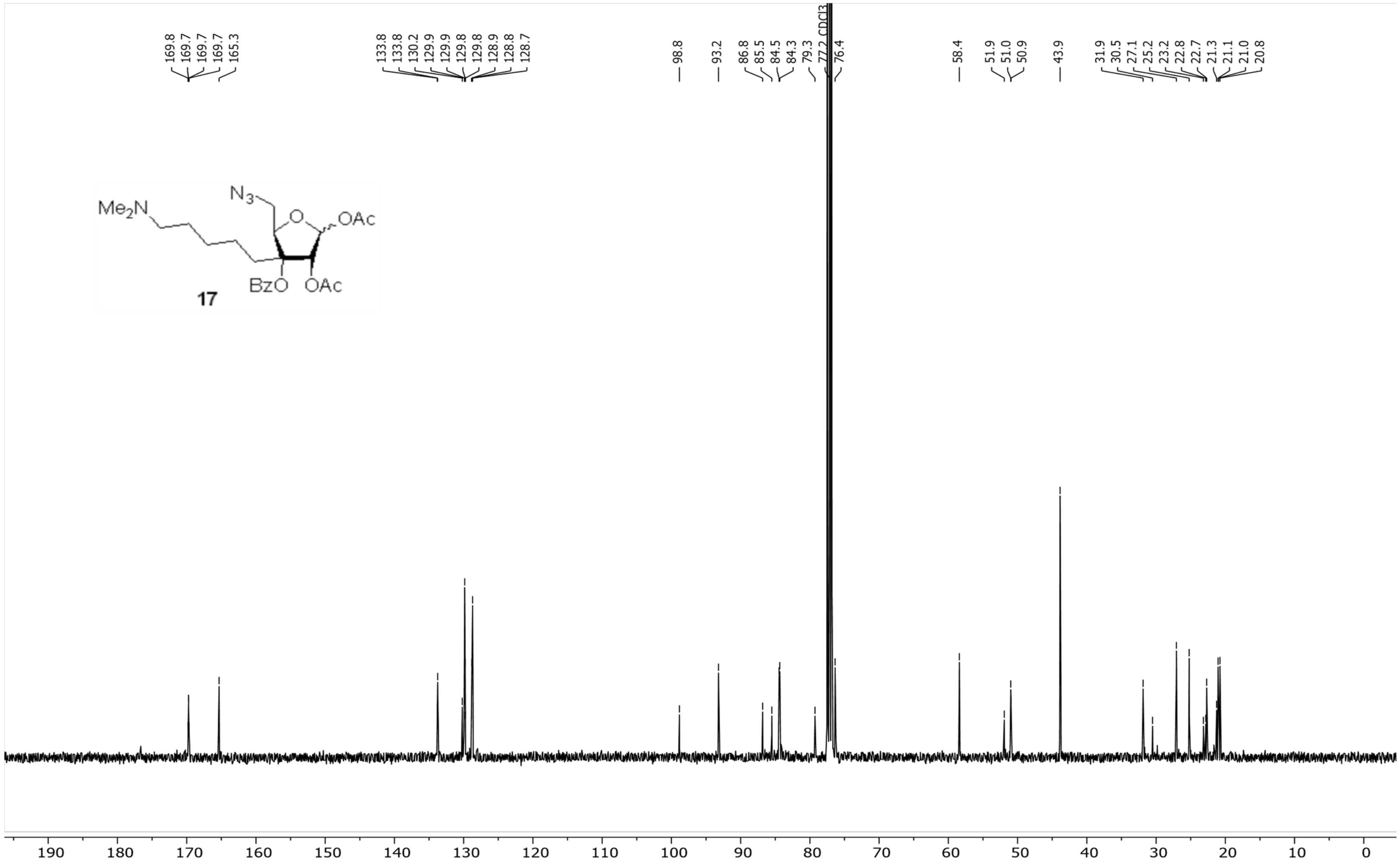

5-*O*-[5‴-Azido-5‴-deoxy-2‴-*O*-acetyl-3-*C*-(5-(dimethylamino)pentyl)-3-*O*-benzoyl-α-D-ribofuranosyl]-6,2″,3″,6″-tetra-*O*-benzoyl-1,3,2′,4″-tetraazido-1,3,2′,4″-tetra(desamino)-6′,7′-oxazolidino-apramycin trifluoroacetate (19α)

[^{1}H NMR, 400 MHz, $CDCl_3$]

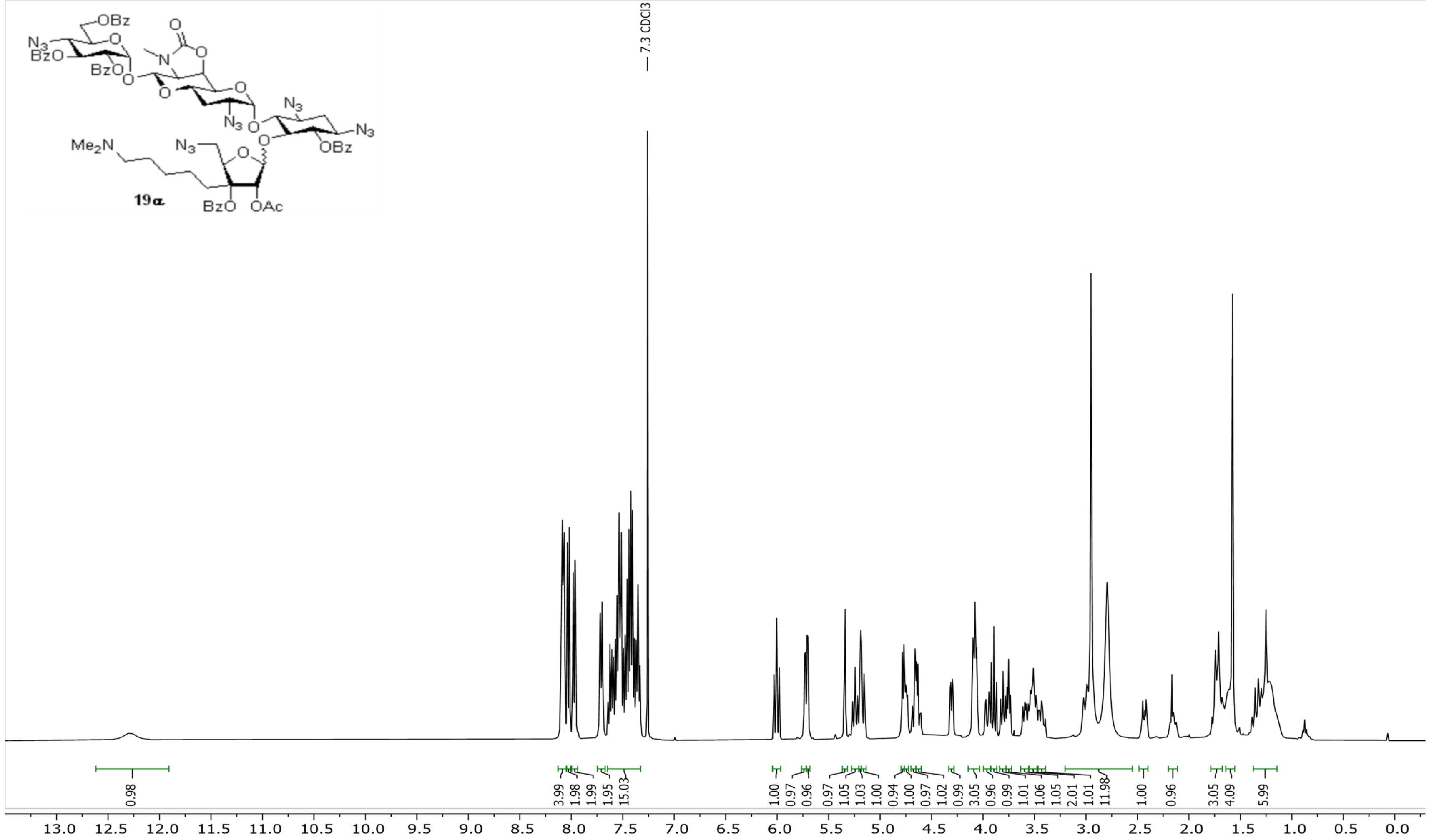

5-*O*-[5‴-Azido-5‴-deoxy-2‴-*O*-acetyl-3-*C*-(5-(dimethylamino)pentyl)-3-*O*-benzoyl-α-D-ribofuranosyl]-6,2″,3″,6″-tetra-*O*-benzoyl-1,3,2′,4″-tetraazido-1,3,2′,4″-tetra(desamino)-6′,7′-oxazolidino-apramycin trifluoroacetate (19α)

[^{13}C NMR, 101 MHz, $CDCl_3$]

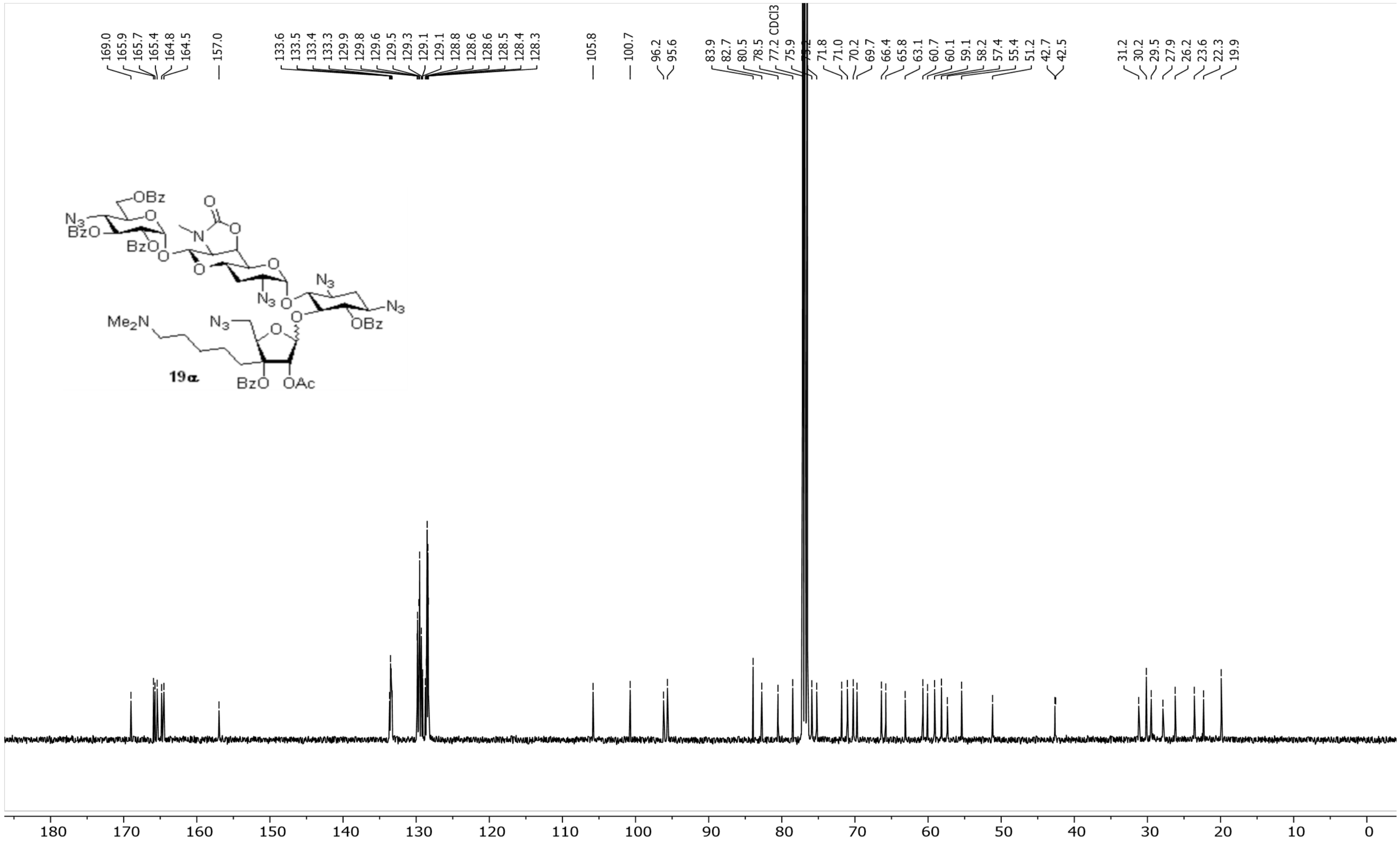

5-*O*-[5‴-Azido-5‴-deoxy-2‴-*O*-acetyl-3-*C*-(5-(dimethylamino)pentyl)-3-*O*-benzoyl-α-D-ribofuranosyl]-6,2″,3″,6″-tetra-*O*-benzoyl-1,3,2′,4″-tetraazido-1,3,2′,4″-tetra(desamino)-6′,7′-oxazolidino-apramycin trifluoroacetate (19α)
[HSQC, 400 MHz, $CDCl_3$]

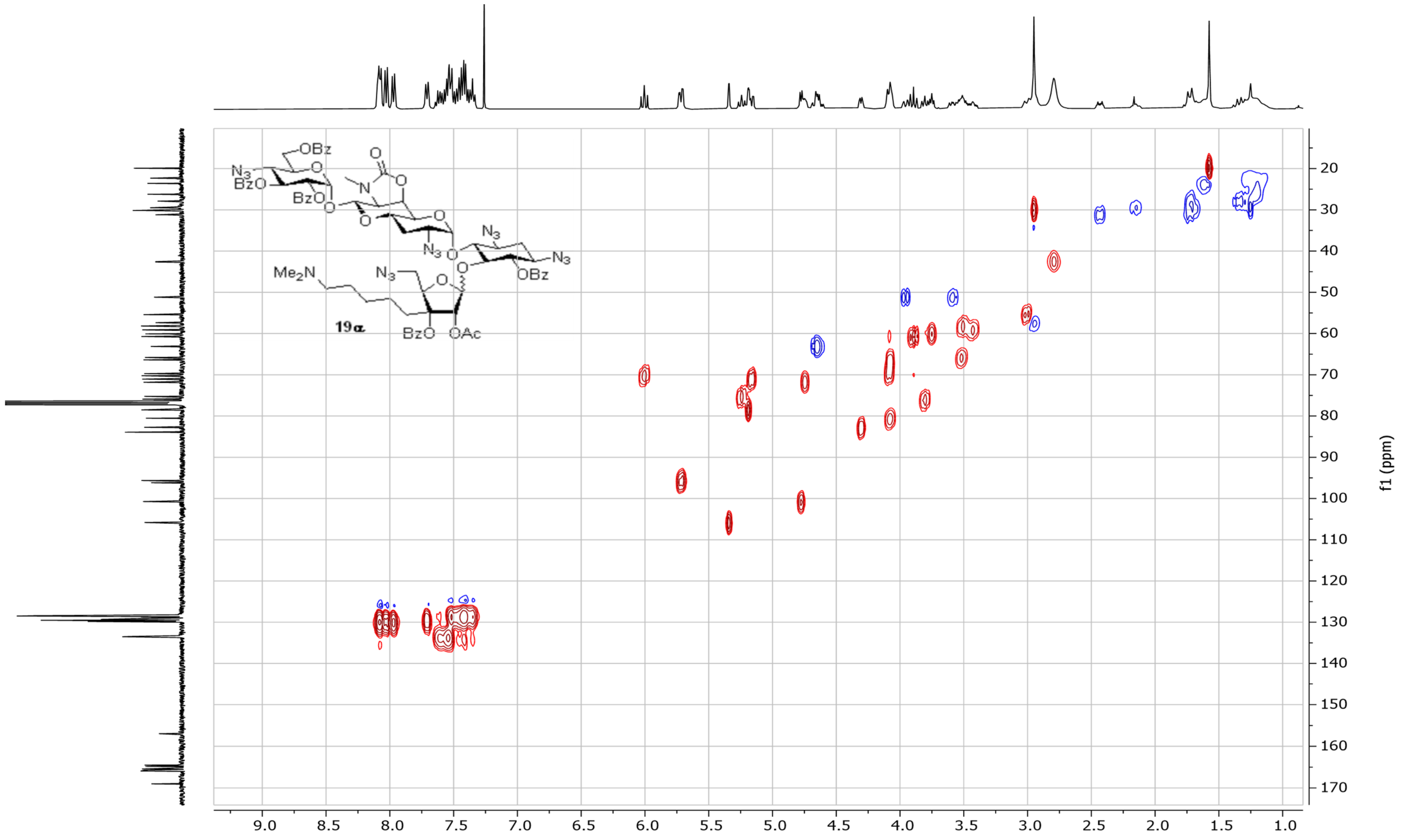

5-*O*-[5‴-Azido-5‴-deoxy-2‴-*O*-acetyl-3-*C*-(5-(dimethylamino)pentyl)-3-*O*-benzoyl-α-D-ribofuranosyl]-6,2″,3″,6″-tetra-*O*-benzoyl-1,3,2′,4″-tetraazido-1,3,2′,4″-tetra(desamino)-6′,7′-oxazolidino-apramycin trifluoroacetate (19α)
[HMBC, 400 MHz, $CDCl_3$]

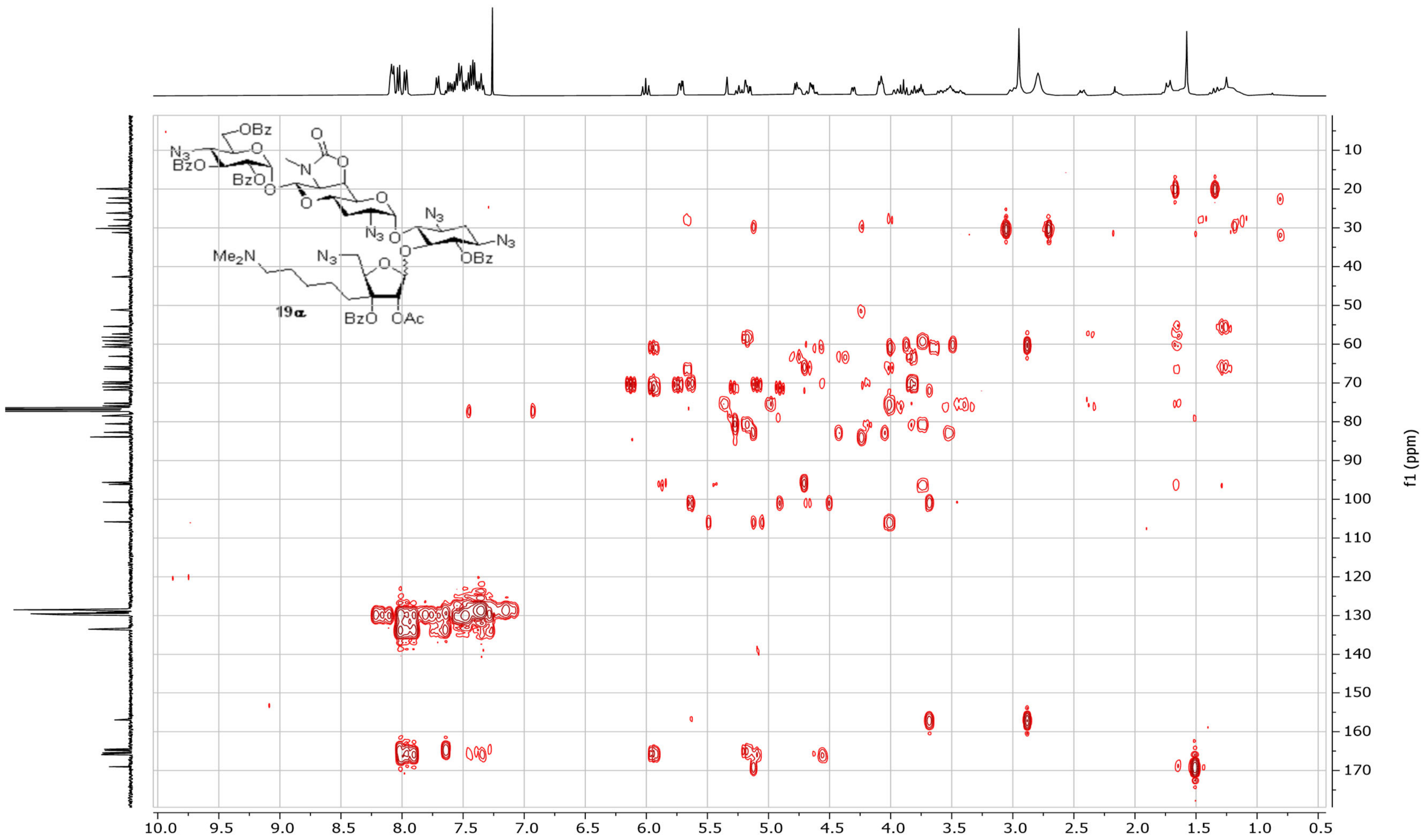

5-*O*-[5‴-Azido-5‴-deoxy-2‴-*O*-acetyl-3-*C*-(5-(dimethylamino)pentyl)-3-*O*-benzoyl-α-D-ribofuranosyl]-6,2″,3″,6″-tetra-*O*-benzoyl-1,3,2′,4″-tetraazido-1,3,2′,4″-tetra(desamino)-6′,7′-oxazolidino-apramycin trifluoroacetate (19α)

[COSY, 400 MHz, $CDCl_3$]

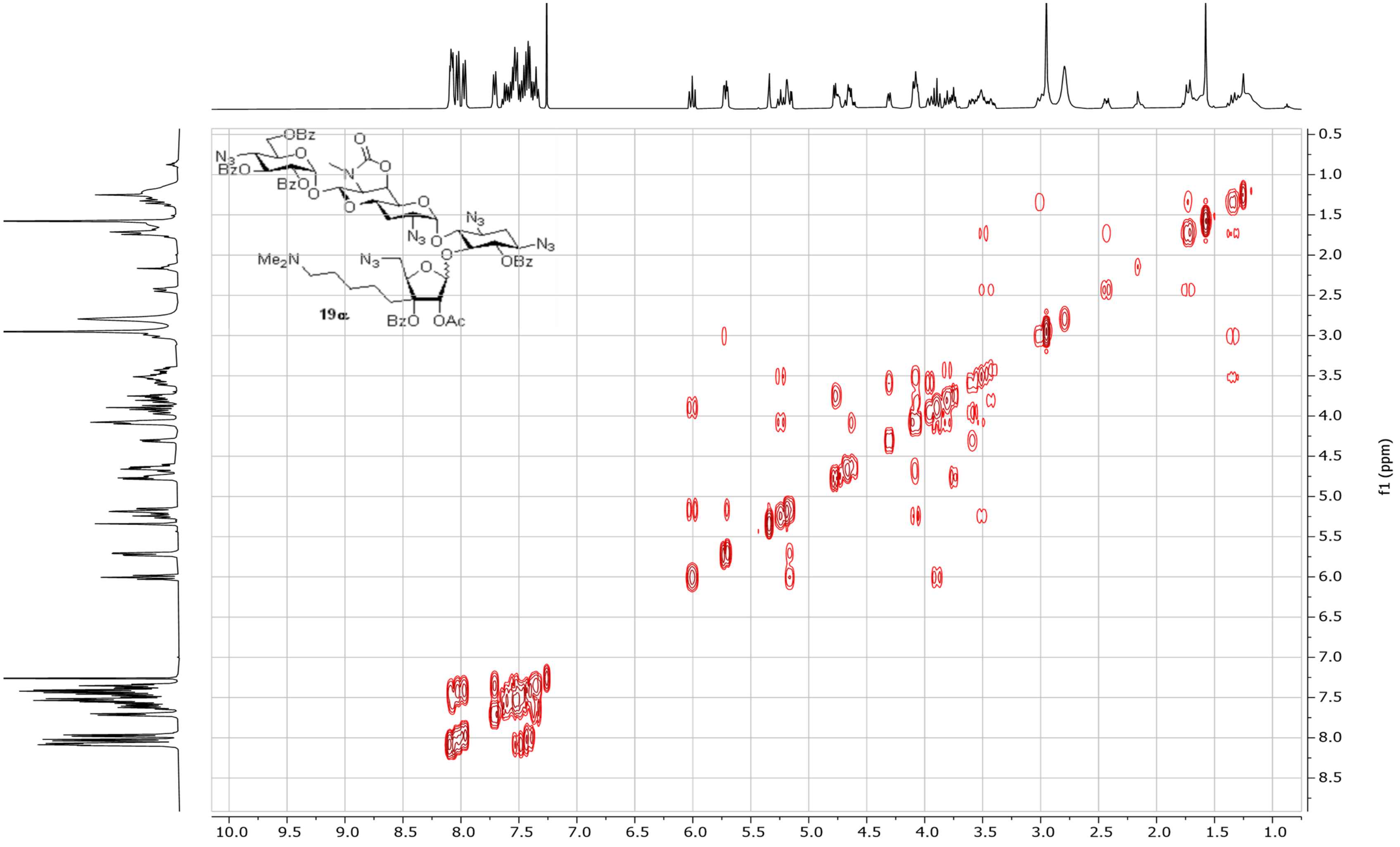

5-*O*-[5‴-Azido-5‴-deoxy-2‴-*O*-acetyl-3-*C*-(5-(dimethylamino)pentyl)-3-*O*-benzoyl-β-D-ribofuranosyl]-6,2″,3″,6″-tetra-*O*-benzoyl-1,3,2′,4″-tetraazido-1,3,2′,4″-tetra(desamino)-6′,7′-oxazolidino-apramycin trifluoroacetate (19β)

[^{1}H NMR, 400 MHz, $CDCl_3$]

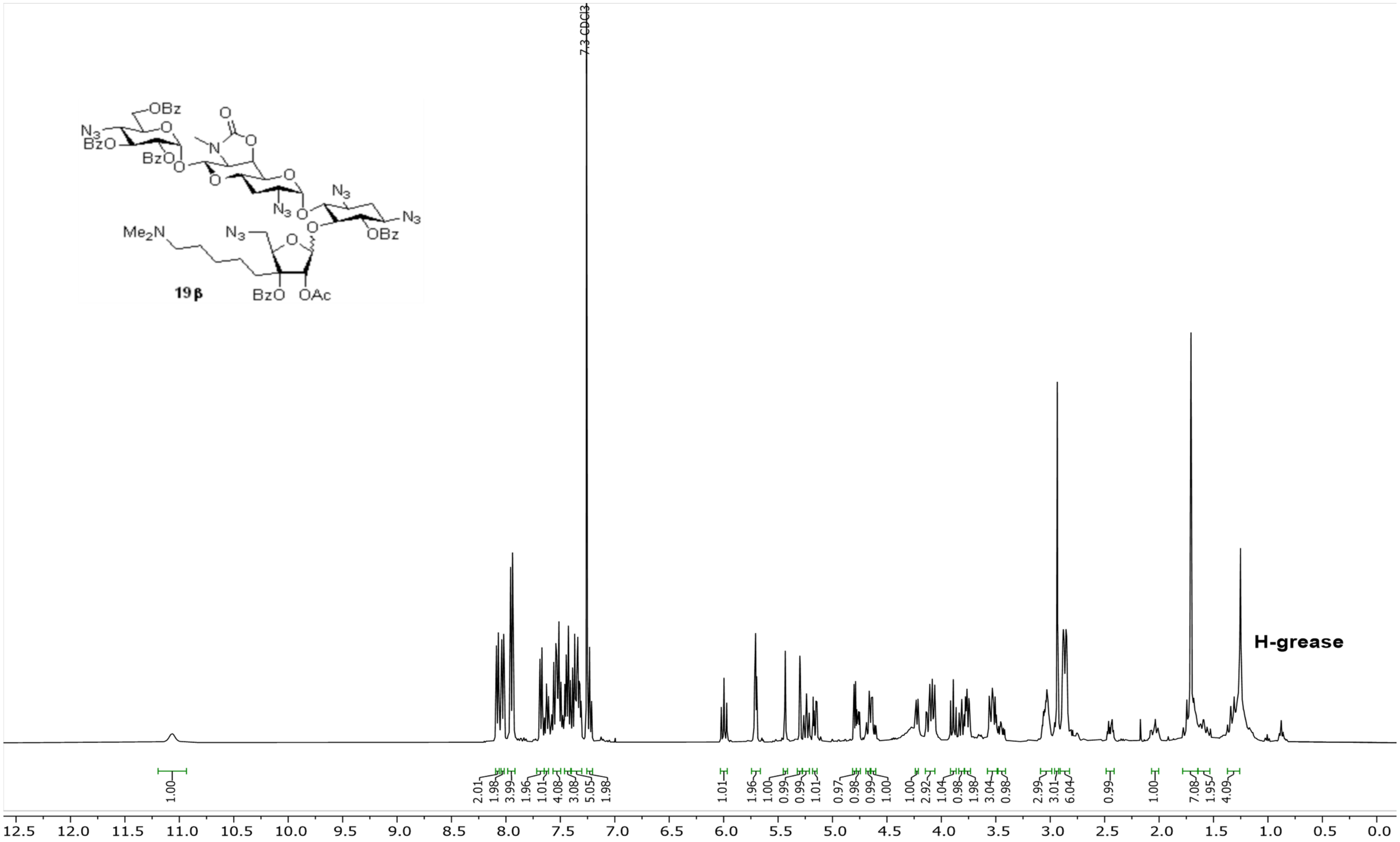

5-*O*-[5‴-Azido-5‴-deoxy-2‴-*O*-acetyl-3-*C*-(5-(dimethylamino)pentyl)-3-*O*-benzoyl-β-D-ribofuranosyl]-6,2″,3″,6″-tetra-*O*-benzoyl-1,3,2′,4″-tetraazido-1,3,2′,4″-tetra(desamino)-6′,7′-oxazolidino-apramycin trifluoroacetate (19β)

[^{13}C NMR, 101 MHz, $CDCl_3$]

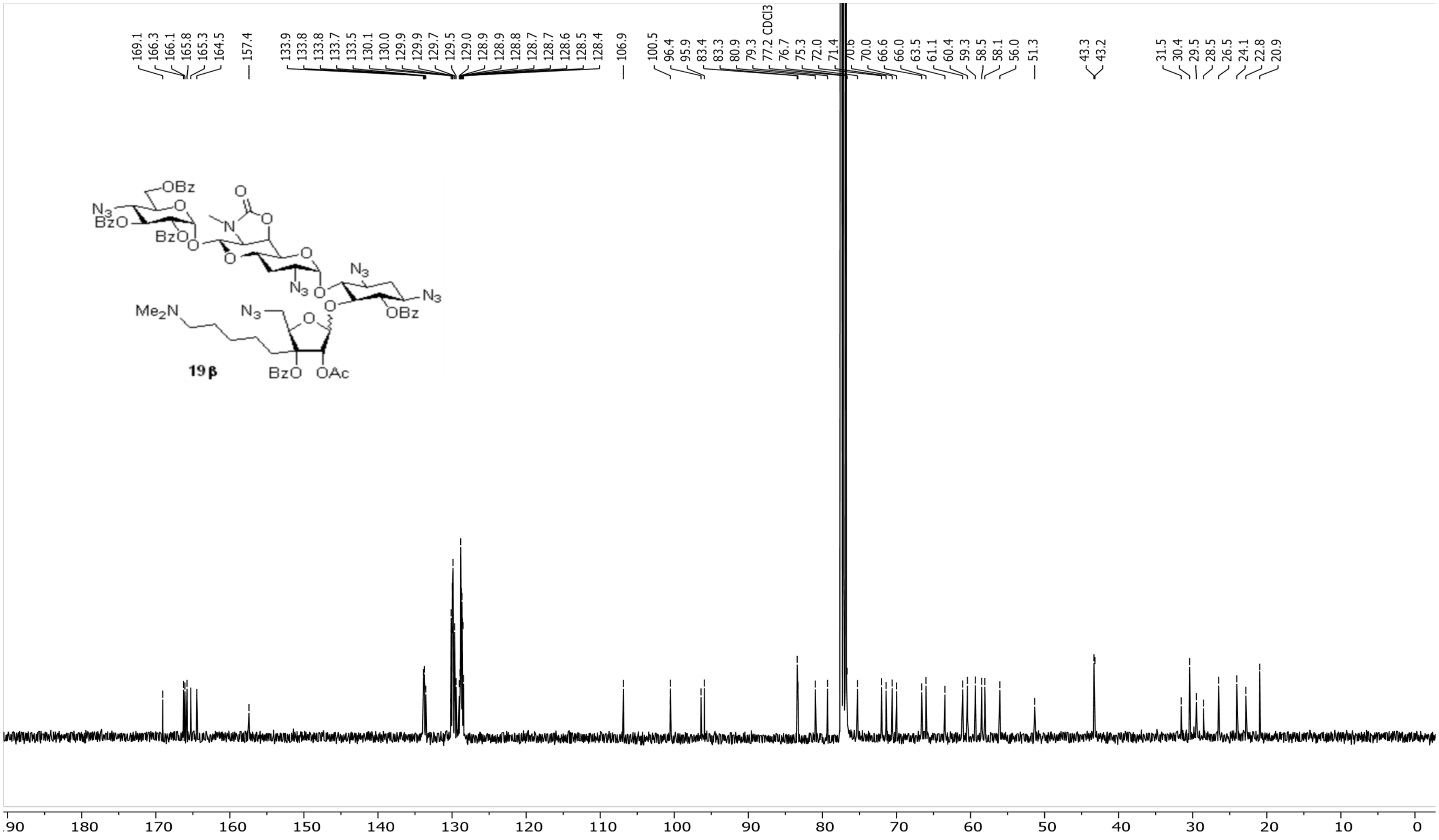

5-*O*-[5‴-Azido-5‴-deoxy-2‴-*O*-acetyl-3-*C*-(5-(dimethylamino)pentyl)-3-*O*-benzoyl-β-D-ribofuranosyl]-6,2″,3″,6″-tetra-*O*-benzoyl-1,3,2′,4″-tetraazido-1,3,2′,4″-tetra(desamino)-6′,7′-oxazolidino-apramycin trifluoroacetate (19β)

[HSQC, 400 MHz, $CDCl_3$]

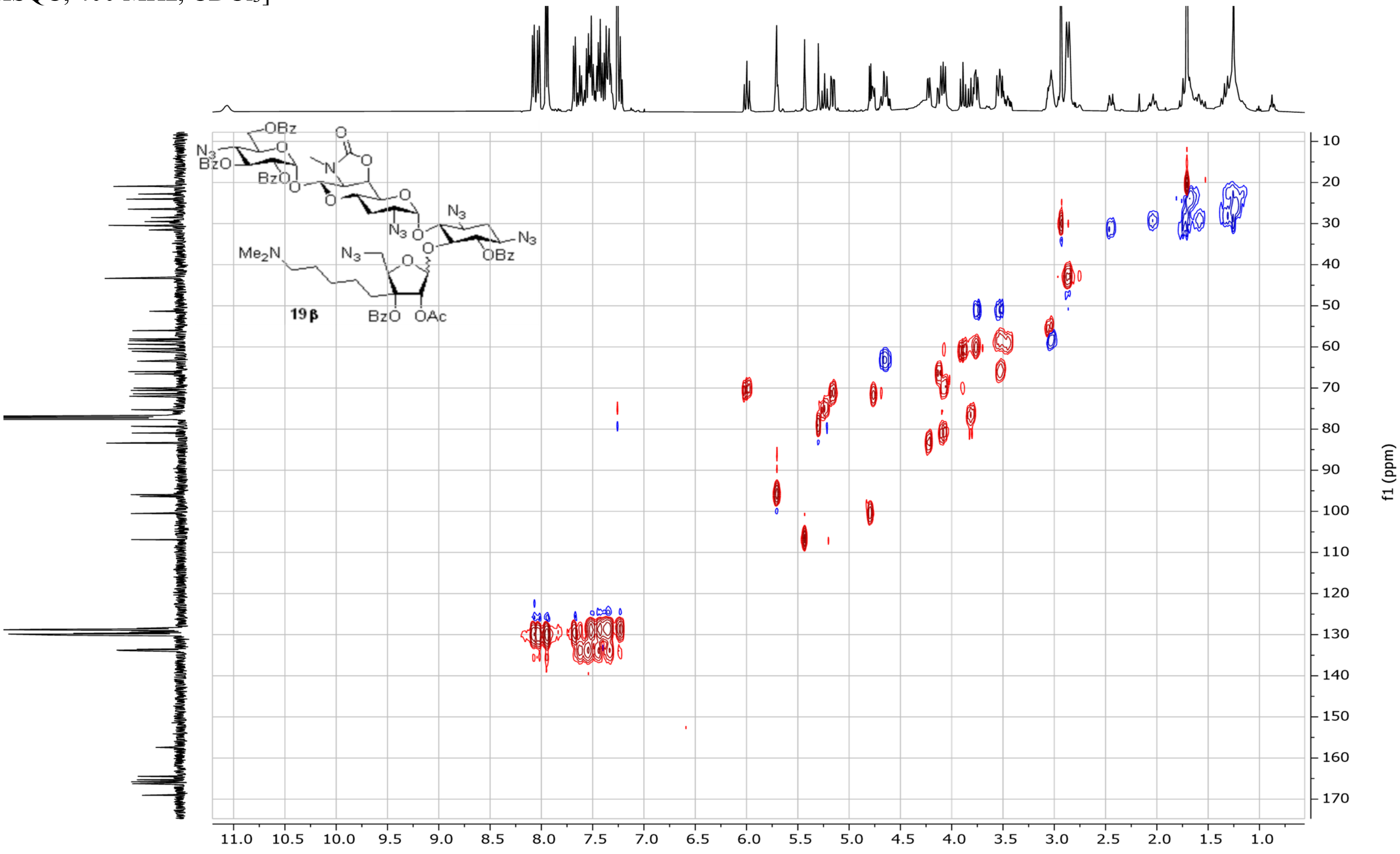

5-*O*-[5‴-Azido-5‴-deoxy-2‴-*O*-acetyl-3-*C*-(5-(dimethylamino)pentyl)-3-*O*-benzoyl-β-D-ribofuranosyl]-6,2″,3″,6″-tetra-*O*-benzoyl-1,3,2′,4″-tetraazido-1,3,2′,4″-tetra(desamino)-6′,7′-oxazolidino-apramycin trifluoroacetate (19β)

[HMBC, 400 MHz, $CDCl_3$]

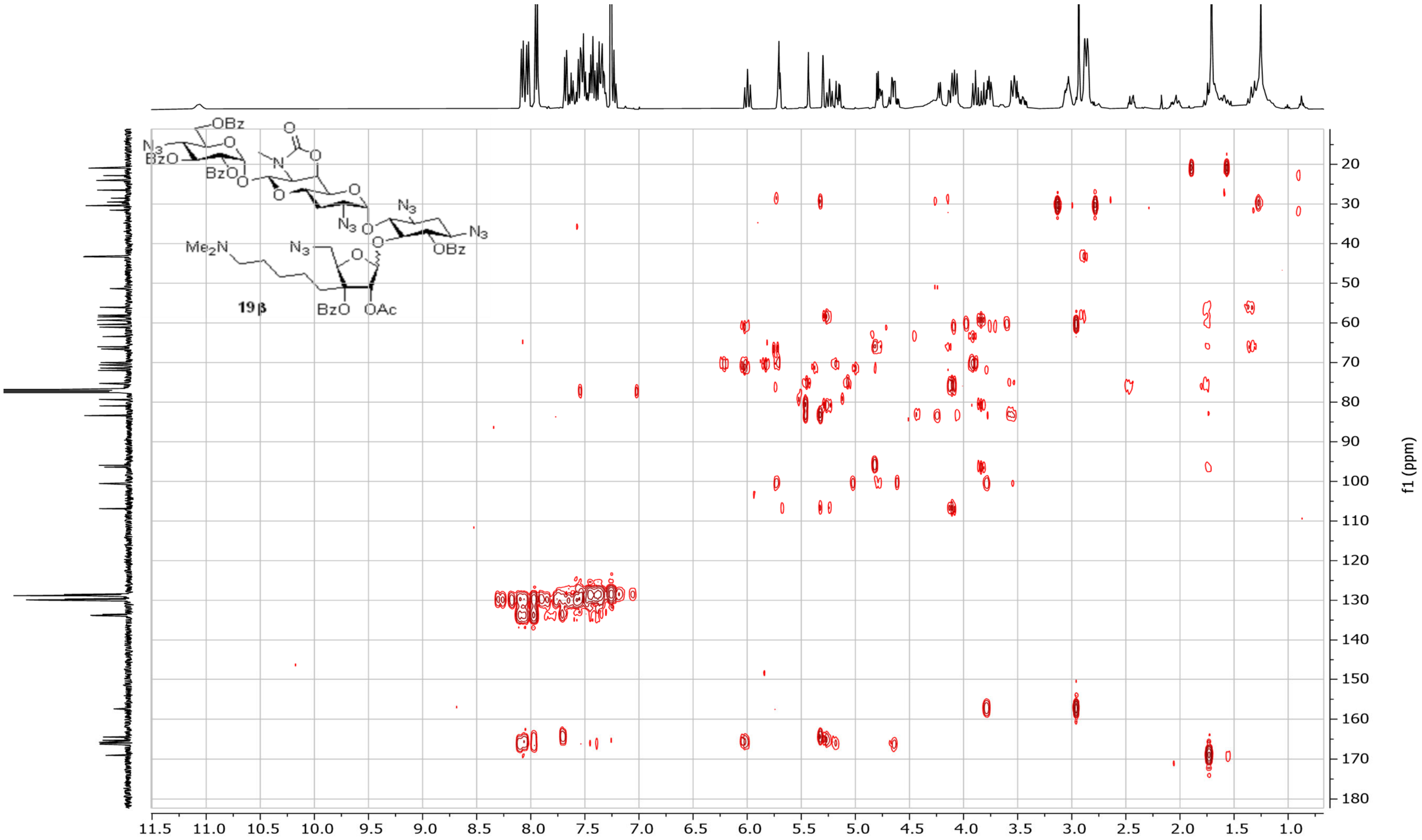

5-*O*-[5‴-Azido-5‴-deoxy-2‴-*O*-acetyl-3-*C*-(5-(dimethylamino)pentyl)-3-*O*-benzoyl-β-D-ribofuranosyl]-6,2″,3″,6″-tetra-*O*-benzoyl-1,3,2′,4″-tetraazido-1,3,2′,4″-tetra(desamino)-6′,7′-oxazolidino-apramycin trifluoroacetate (19β)
[COSY, 400 MHz, $CDCl_3$]

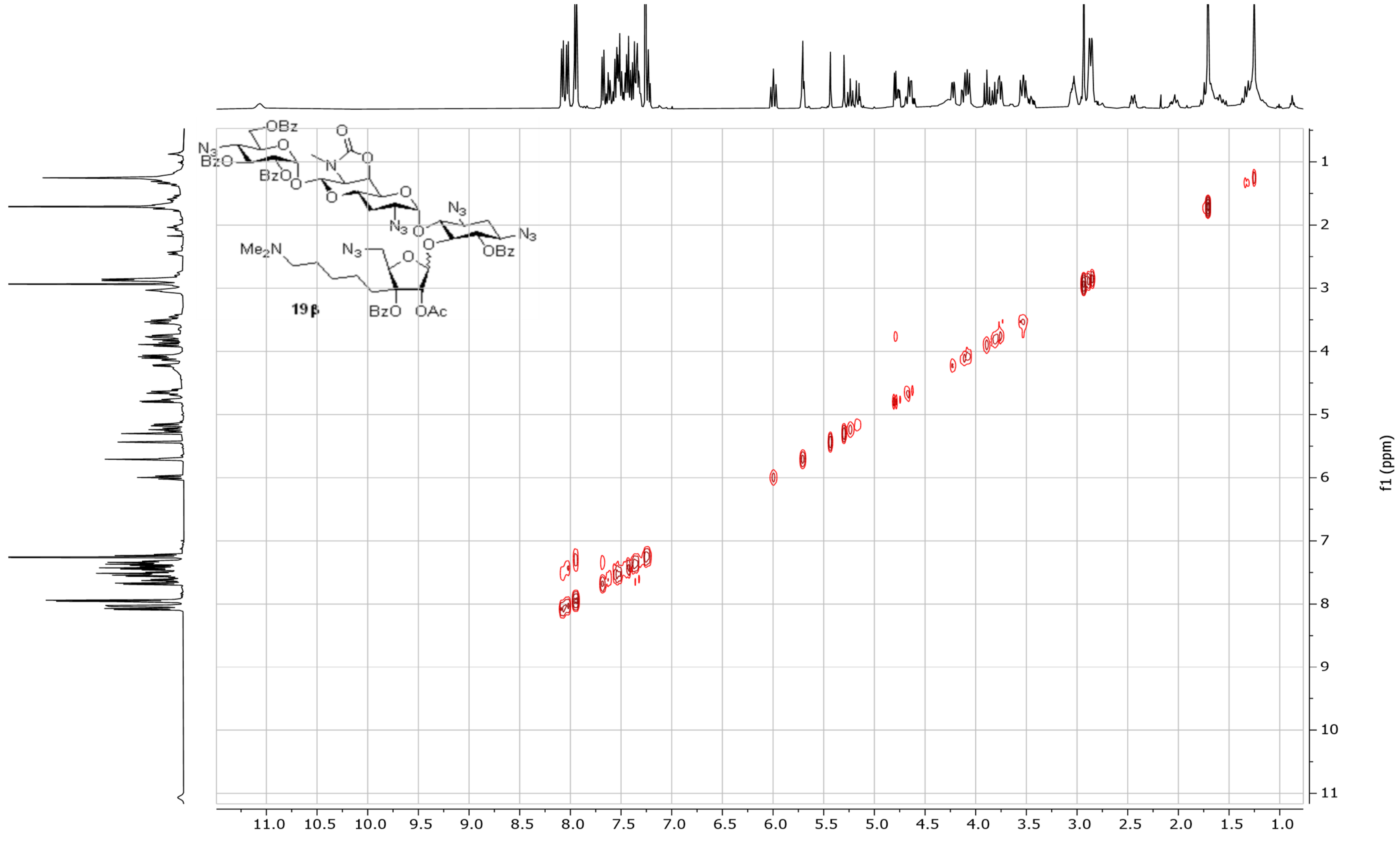

5-*O*-[5′′′-Amino-5′′′-deoxy-3-*C*-(5-(dimethylamino)pentyl)-*β*-D-ribofuranosyl]-apramycin heptaacetate (5)

[^{1}H NMR, 600 MHz, D_2O]

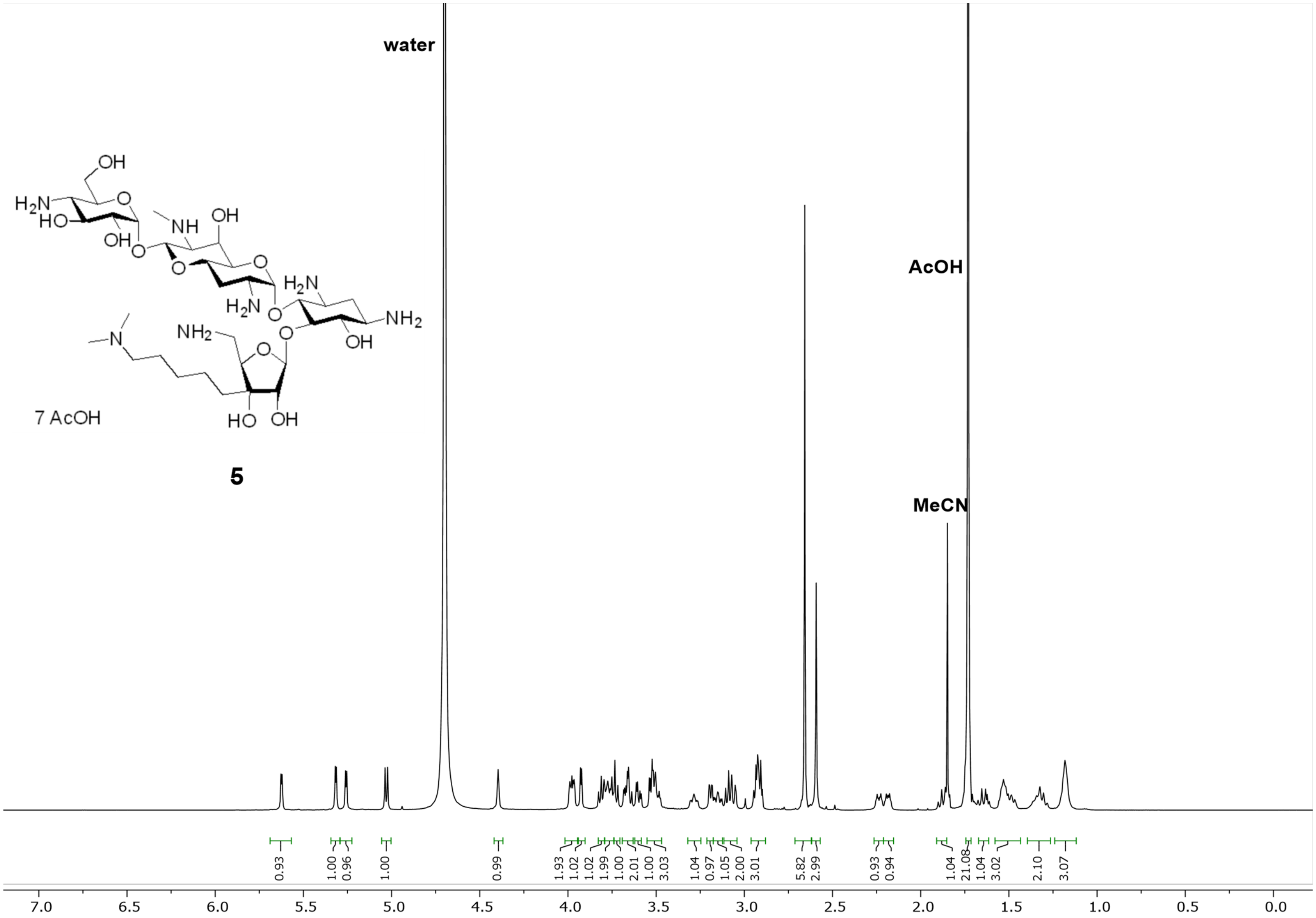

5-*O*-[5‴-Amino-5‴-deoxy-3-*C*-(5-(dimethylamino)pentyl)-*β*-D-ribofuranosyl]-apramycin heptaacetate (5)
[^{13}C NMR, 125 MHz, D_2O]

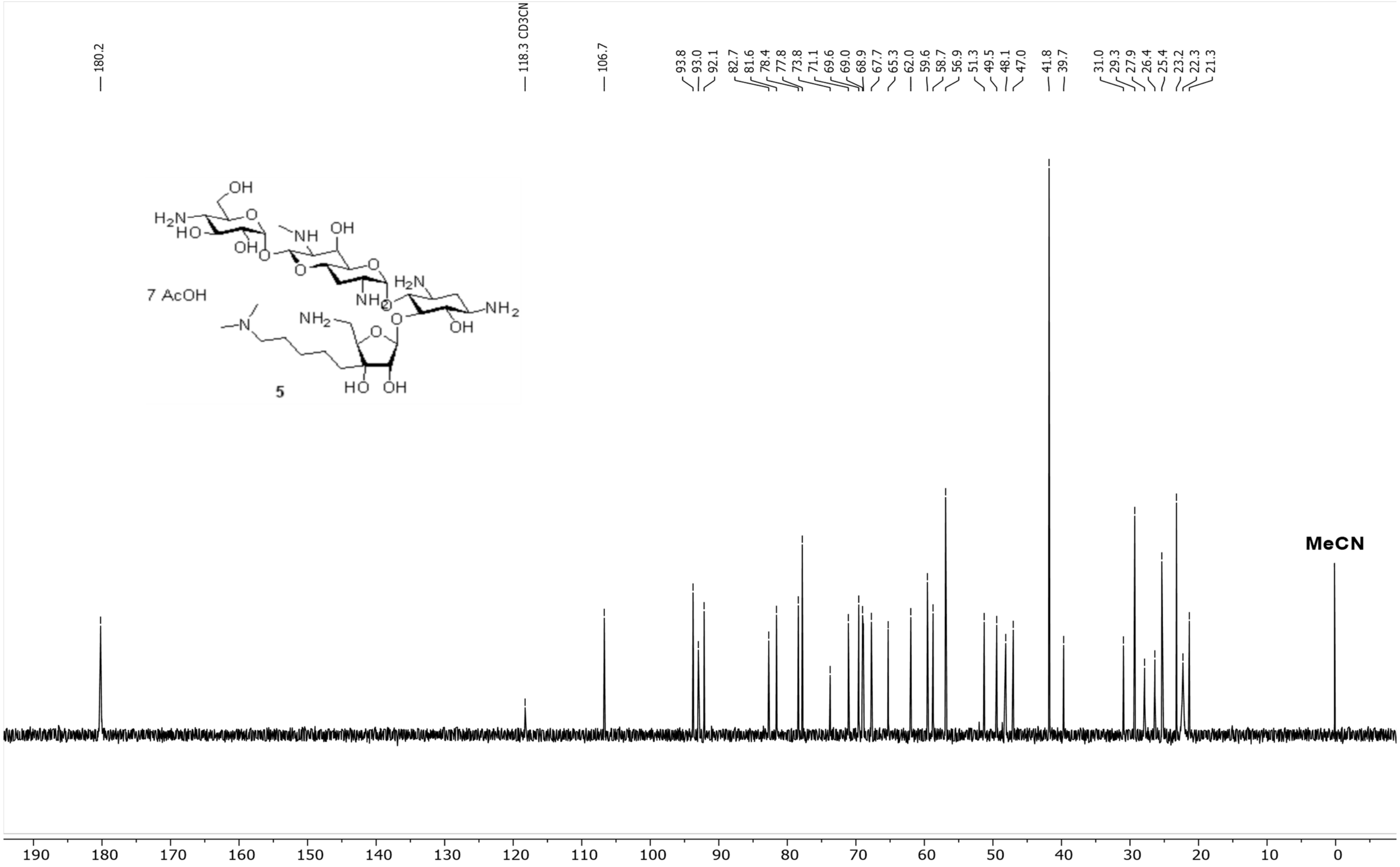

5-*O*-[5‴-Amino-5‴-deoxy-3-*C*-(5-(dimethylamino)pentyl)-*β*-D-ribofuranosyl]-apramycin heptaacetate (5)

[HSQC, 600 MHz, D_2O]

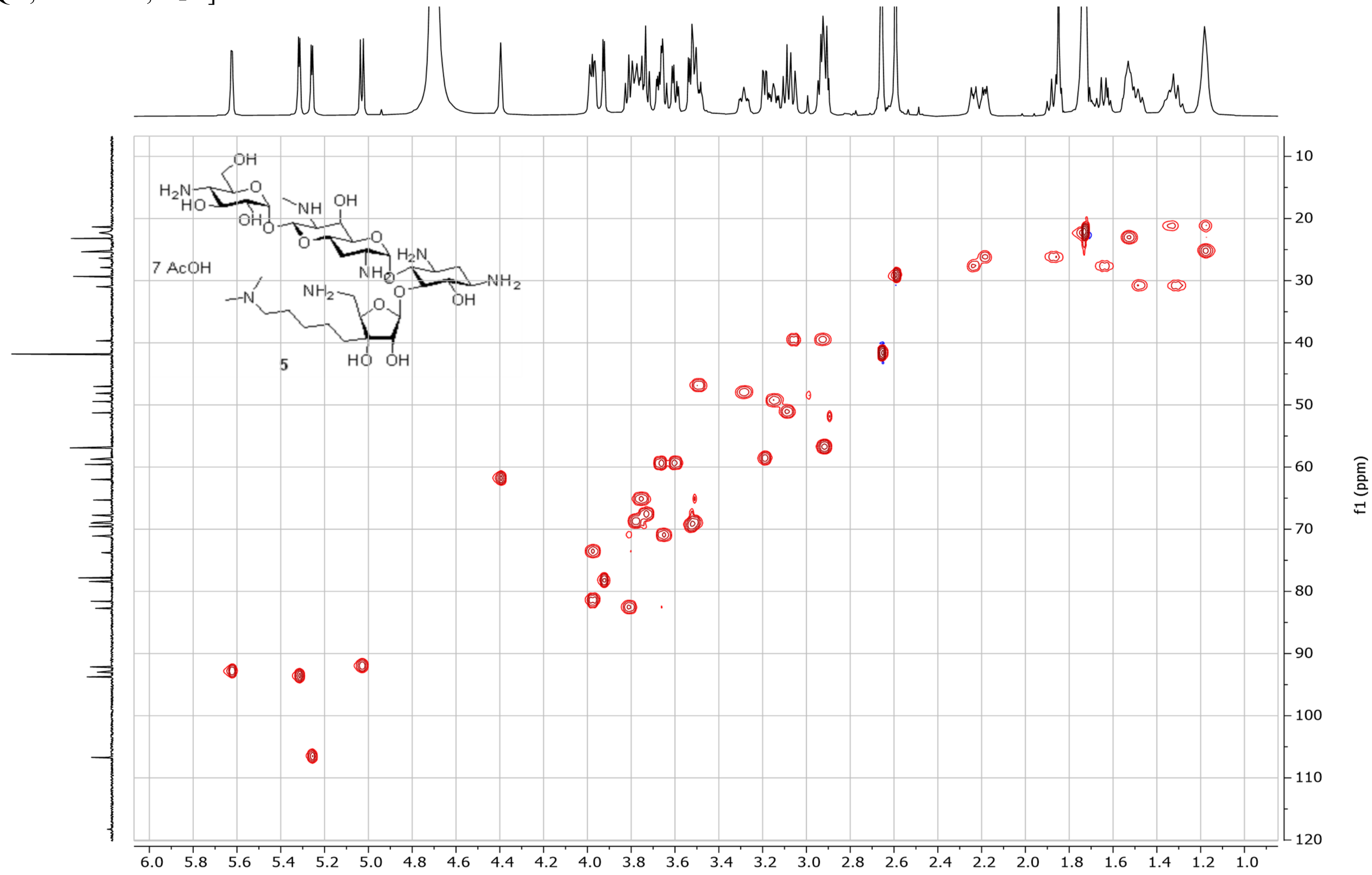

5-*O*-[5‴-Amino-5‴-deoxy-3-*C*-(5-(dimethylamino)pentyl)-*β*-D-ribofuranosyl]-apramycin heptaacetate (5)

[HMBC, 600 MHz, D_2O]

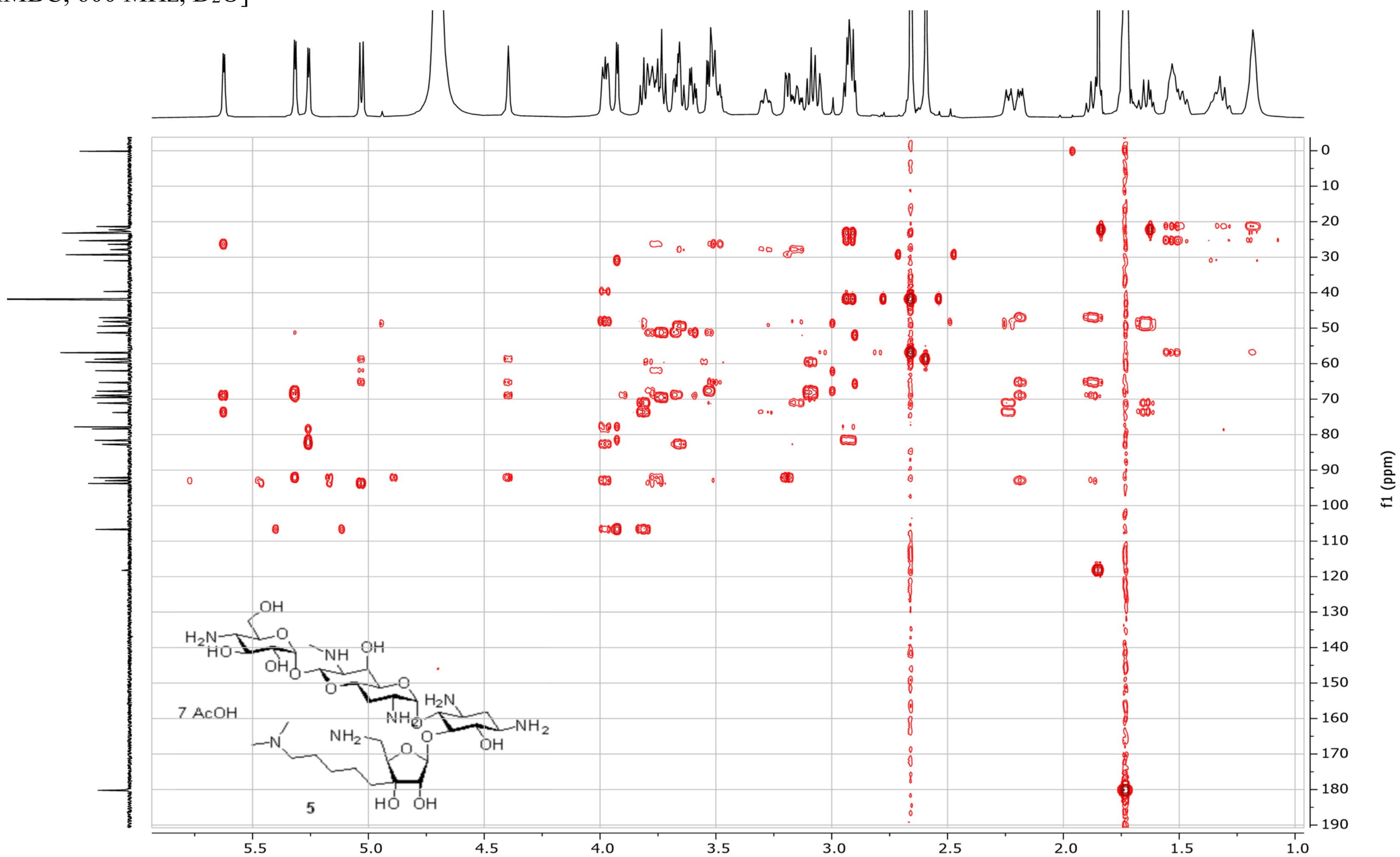

5-*O*-[5‴-Amino-5‴-deoxy-3-*C*-(5-(dimethylamino)pentyl)-*β*-D-ribofuranosyl]-apramycin heptaacetate (5)

[COSY, 600 MHz, D_2O]

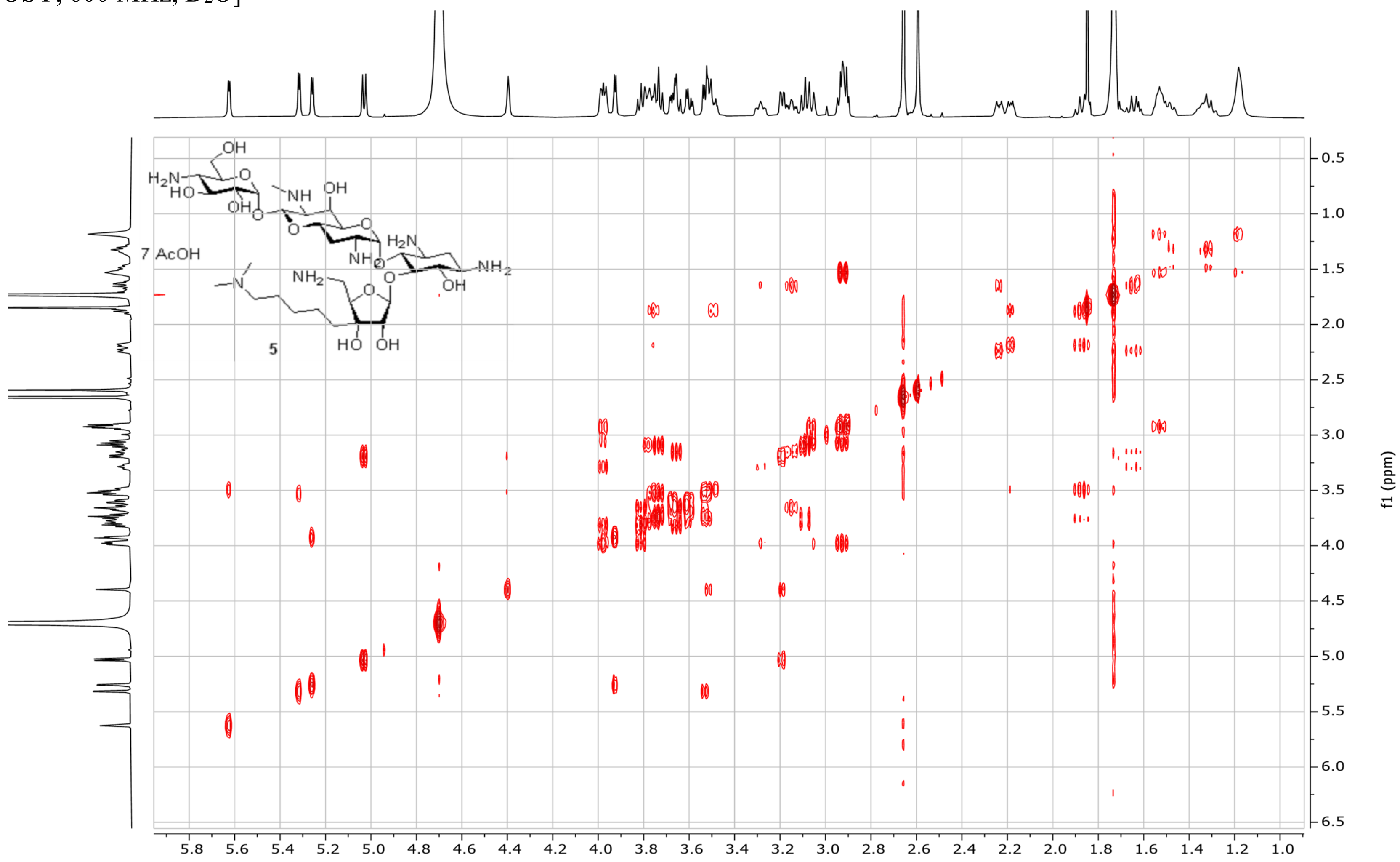

5-*O*-[5′′′-Amino-5′′′-deoxy-3-*C*-(5-(dimethylamino)pentyl)-*β*-D-ribofuranosyl]-apramycin heptaacetate (5)
[NOESY, 600 MHz, D_2O]

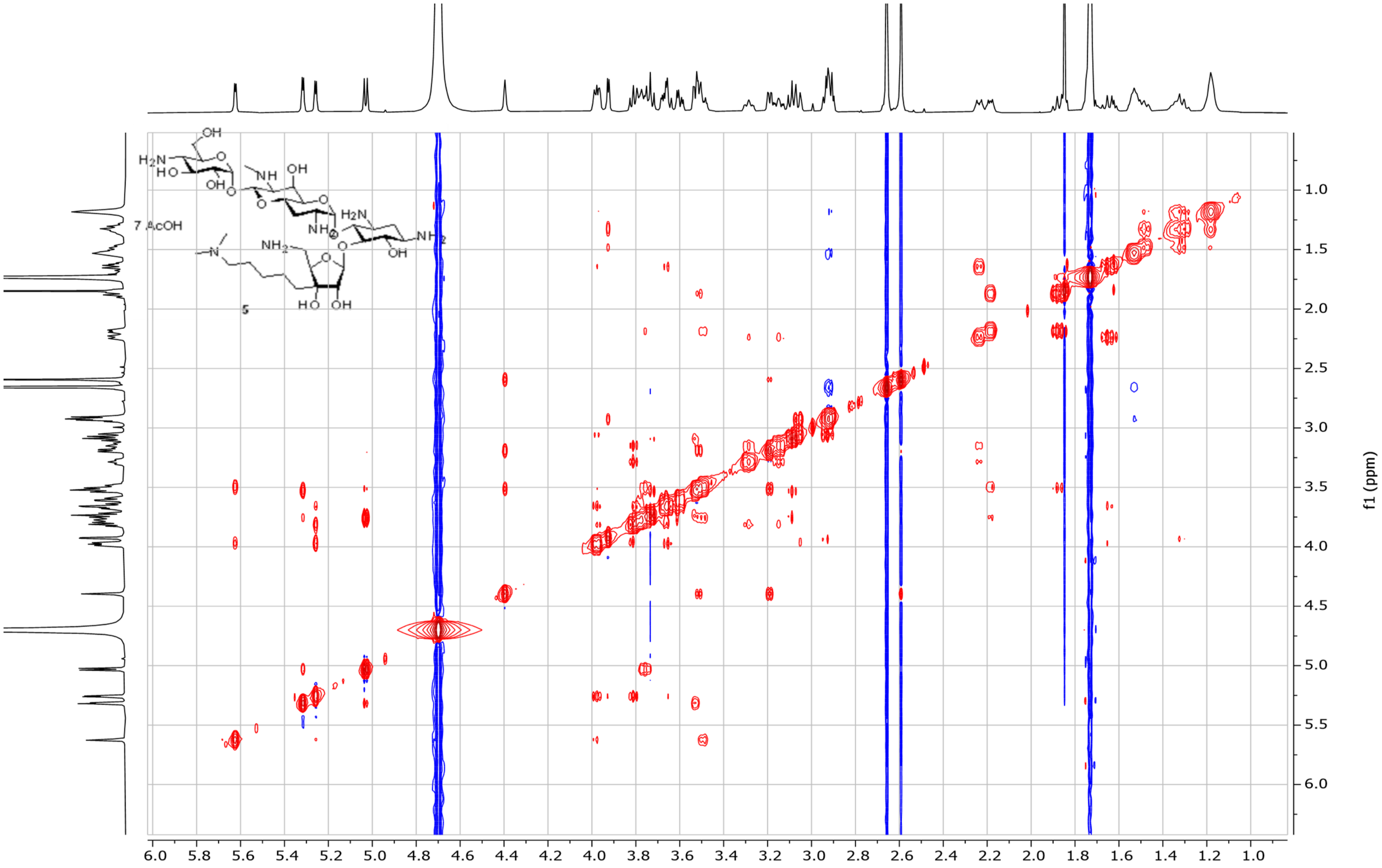

5-*O*-[5′′′-Amino-5′′′-deoxy-3-*C*-(5-(dimethylamino)pentyl)-α-D-ribofuranosyl]-apramycin hexaacetate (6)

[^{1}H NMR, 600 MHz, D_2O]

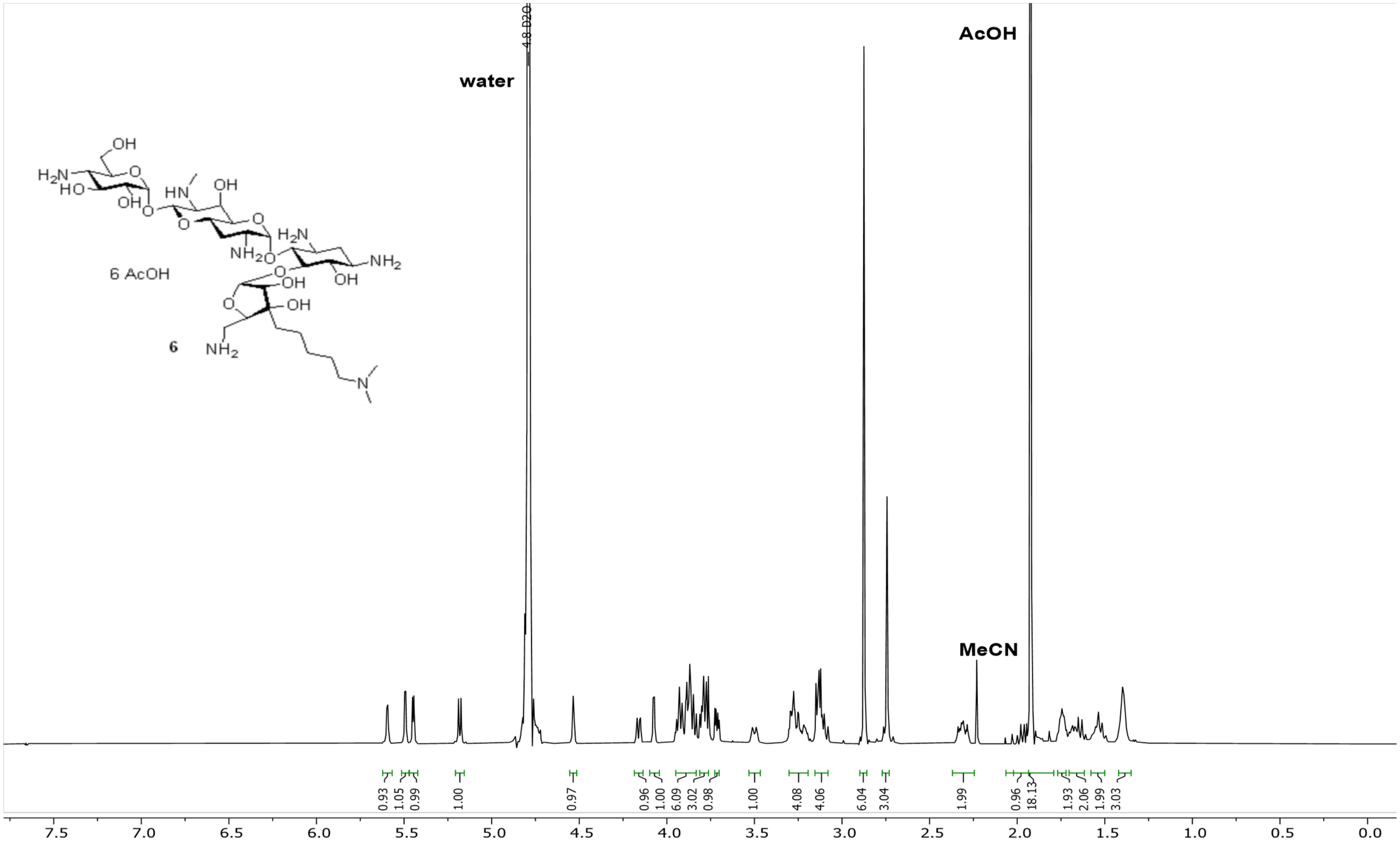

5-*O*-[5′′′-Amino-5′′′-deoxy-3-*C*-(5-(dimethylamino)pentyl)-α-D-ribofuranosyl]-apramycin hexaacetate (6)
[^{13}C NMR, 125 MHz, D_2O]

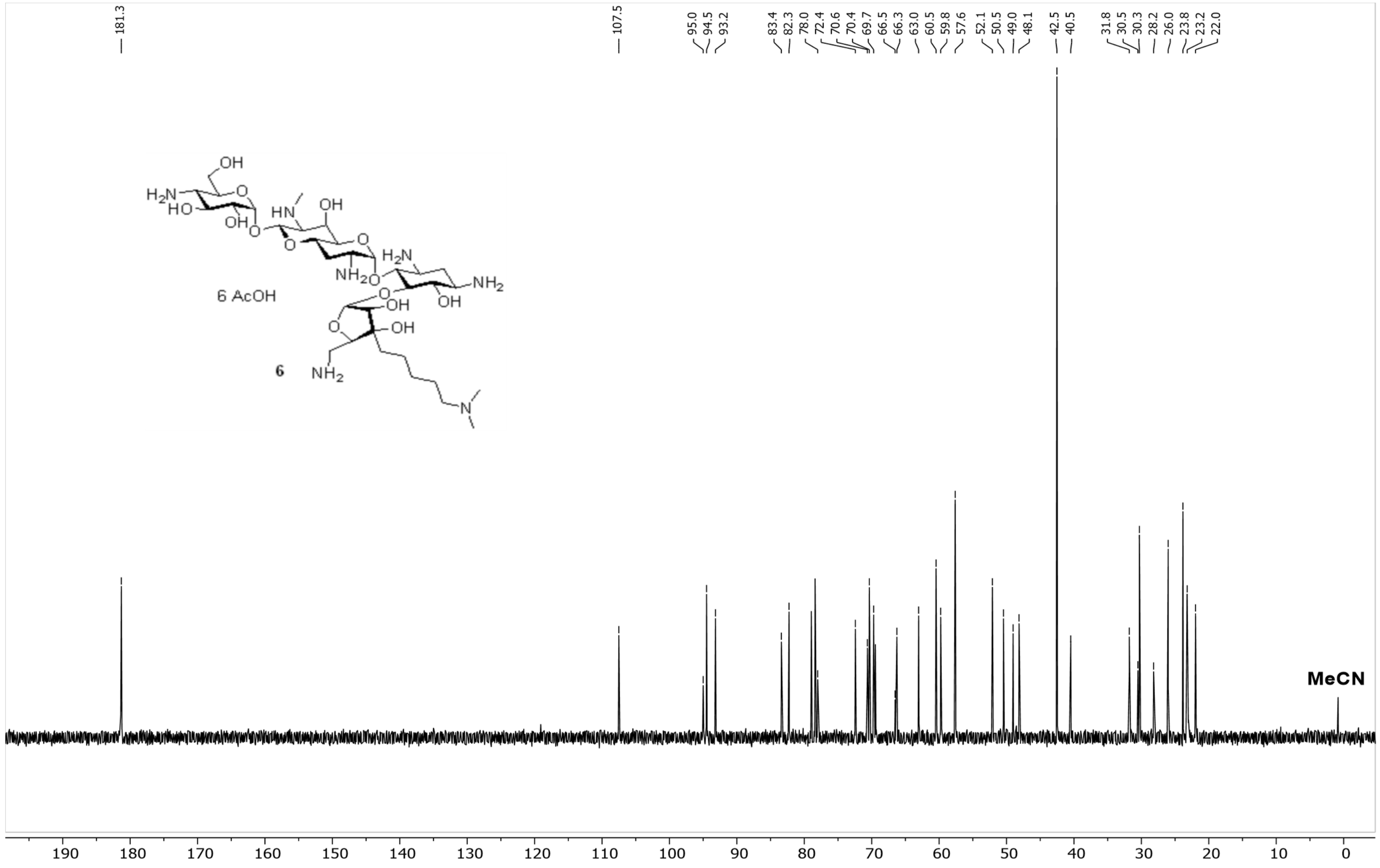

5-*O*-[5′′′-Amino-5′′′-deoxy-3-*C*-(5-(dimethylamino)pentyl)-α-D-ribofuranosyl]-apramycin hexaacetate (6)

[HSQC, 600 MHz, D_2O]

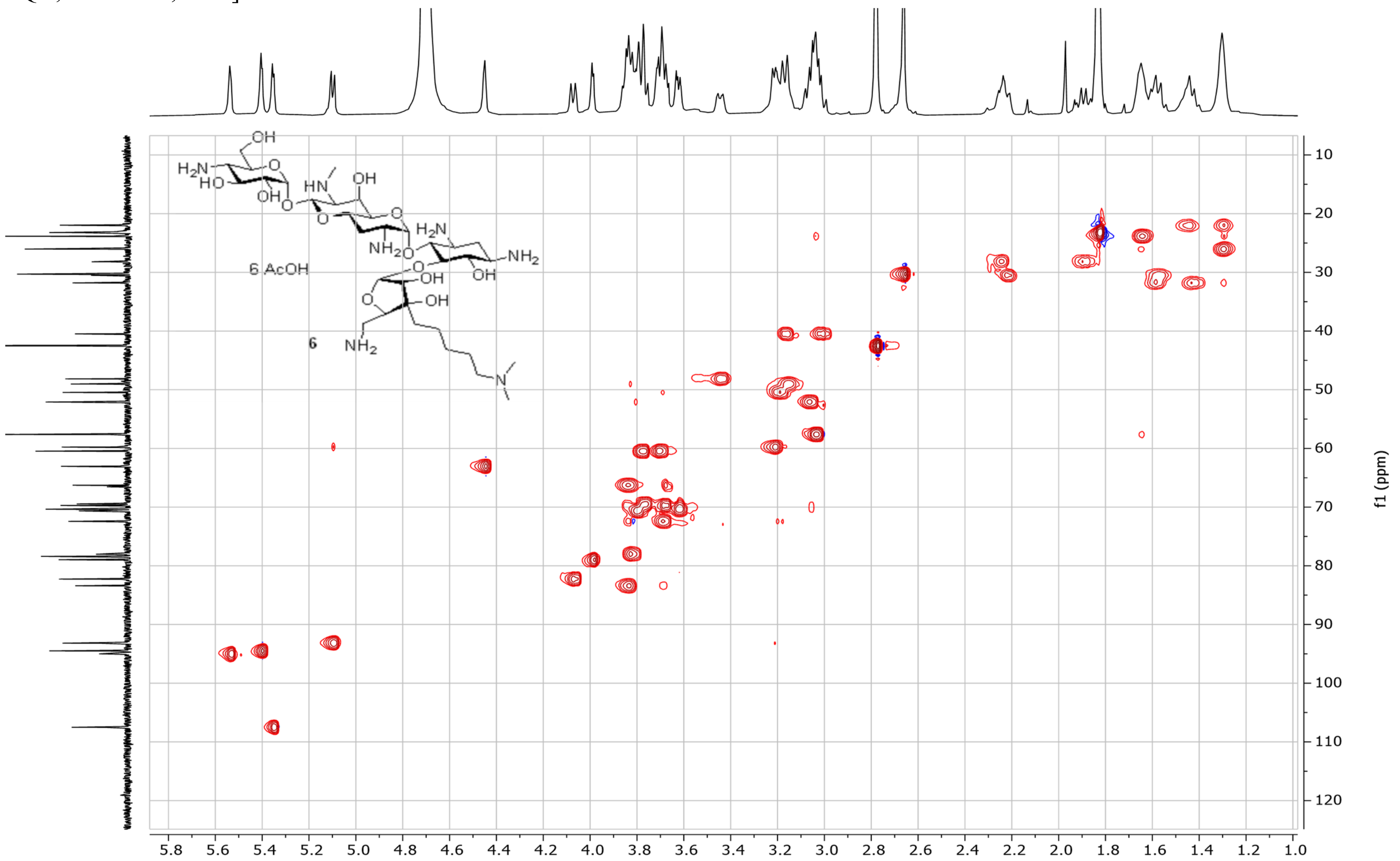

5-*O*-[5‴-Amino-5‴-deoxy-3-*C*-(5-(dimethylamino)pentyl)-α-D-ribofuranosyl]-apramycin hexaacetate (6)

[HMBC, 600 MHz, D_2O]

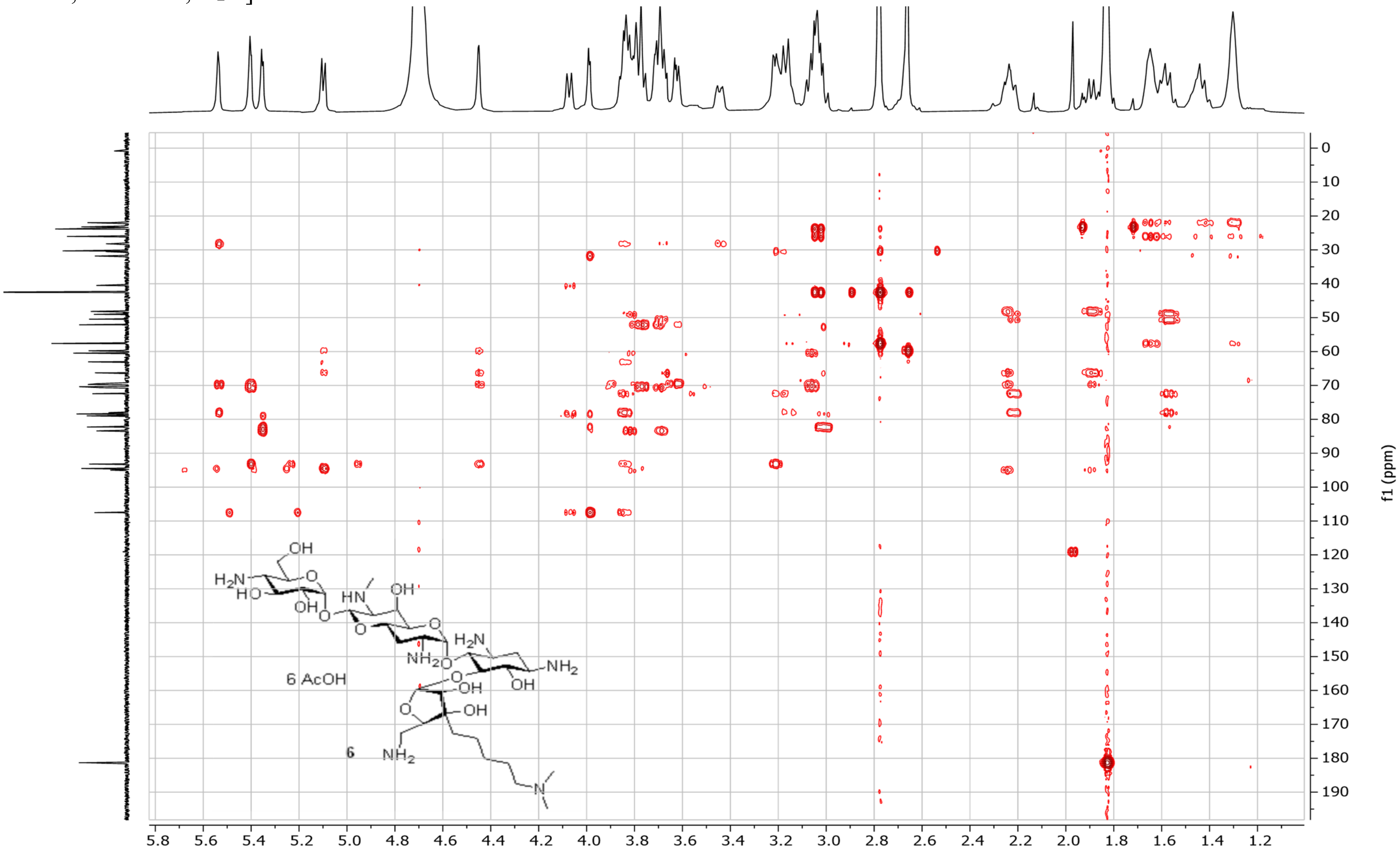

5-*O*-[5‴-Amino-5‴-deoxy-3-*C*-(5-(dimethylamino)pentyl)-α-D-ribofuranosyl]-apramycin hexaacetate (6)

[COSY, 600 MHz, D_2O]

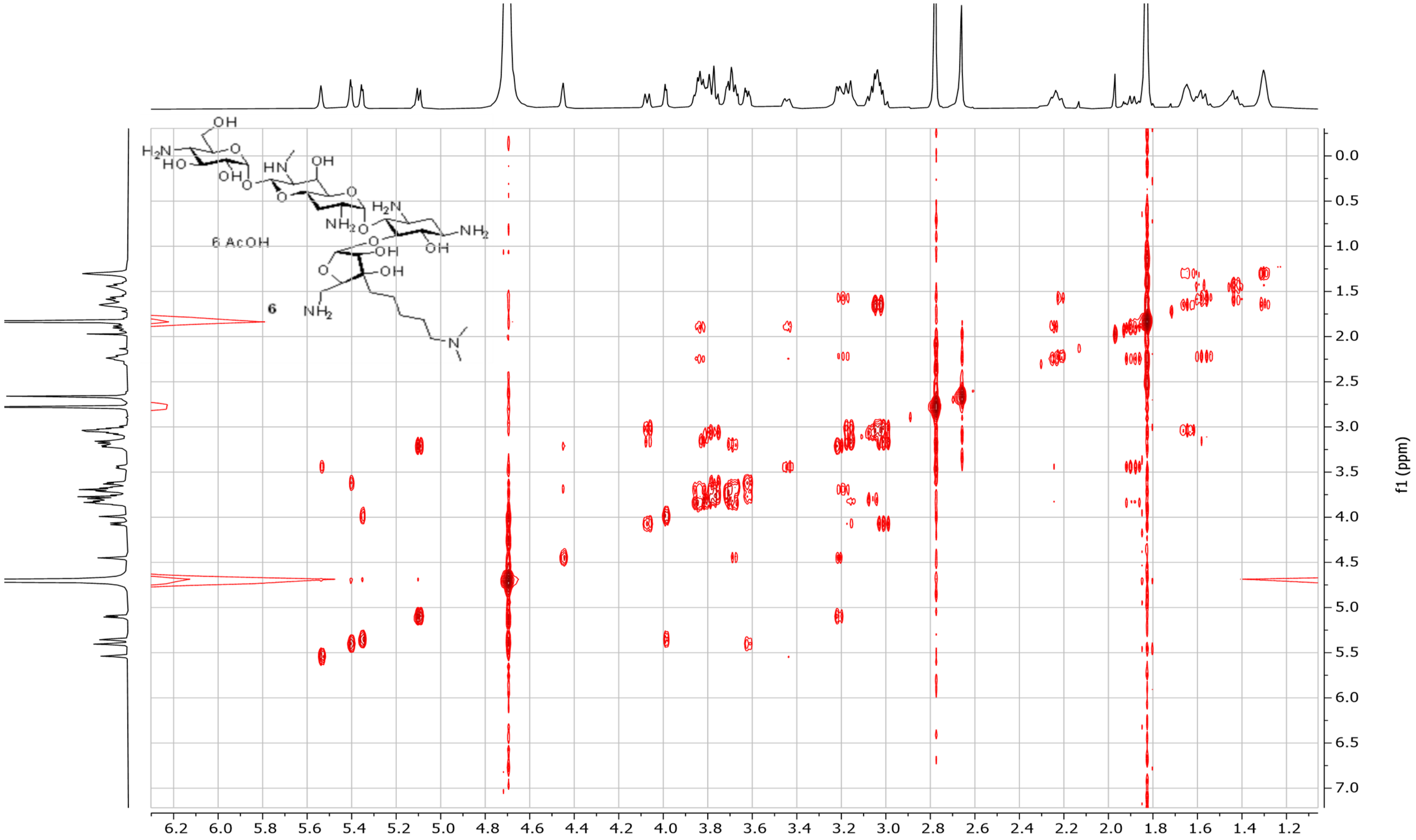

5-*O*-[5′′′-Amino-5′′′-deoxy-3-*C*-(5-(dimethylamino)pentyl)-α-D-ribofuranosyl]-apramycin hexaacetate (6)

[NOESY, 600 MHz, D_2O]

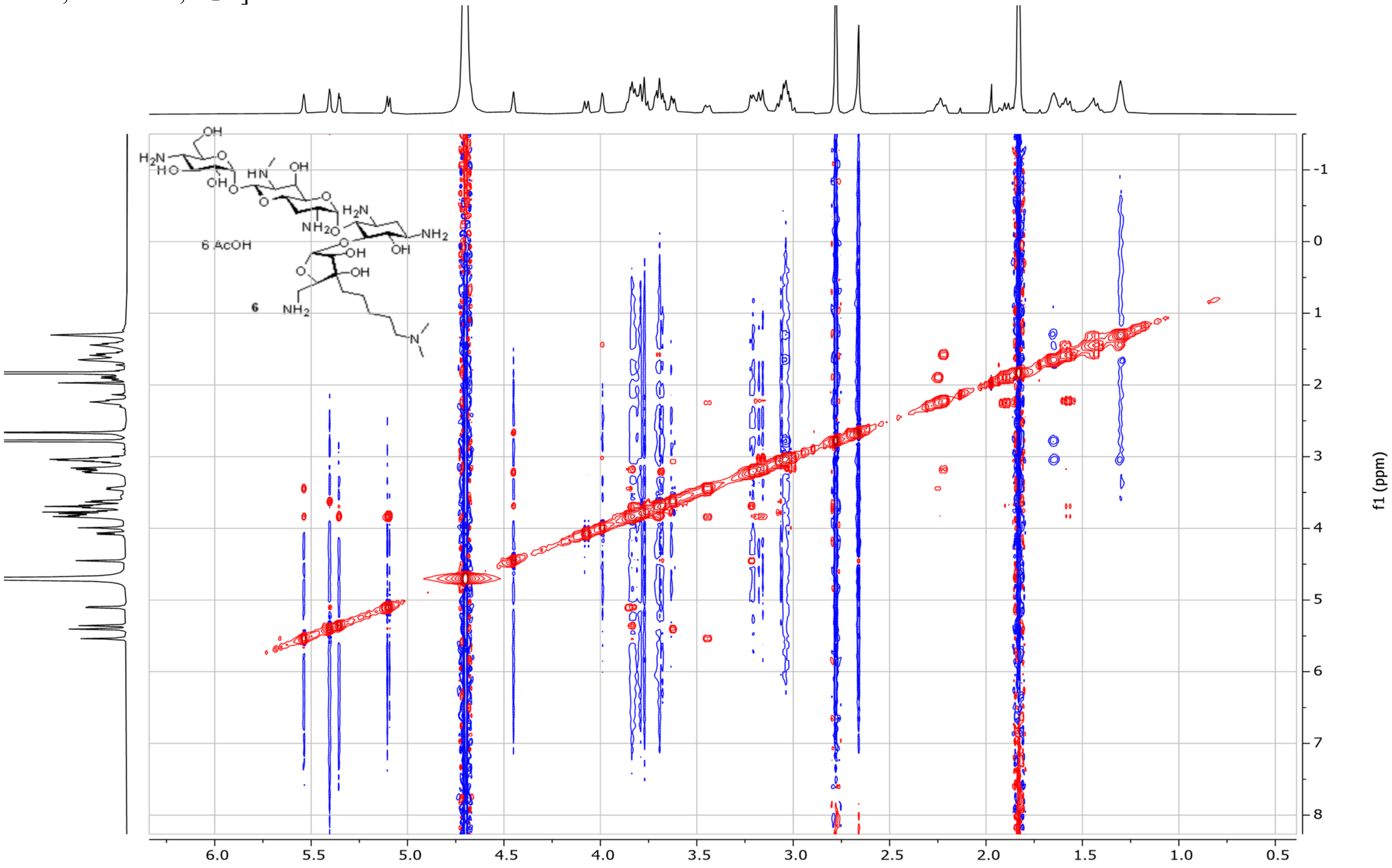

Supplement: Supplementary file 1 [file antibiotics-12-00025-s001.zip › antibiotics-2094535-supplementary.pdf]
